# Making trials more inclusive of people experiencing socioeconomic disadvantage: developing the INCLUDE socioeconomic disadvantage framework

**DOI:** 10.1186/s13063-026-09448-2

**Published:** 2026-01-14

**Authors:** Frances C. Sherratt, Katie Biggs, Heidi R. Green, Liam Bishop, Clara Martins de Barros, Shaun Treweek

**Affiliations:** 1 Department of Public Health, Policy & Systems, University of Liverpool, Liverpool, UK; 2 Sheffield Clinical Trials Research Unit (CTRU), University of Sheffield, Sheffield, UK; 3 Freelance Health Equity and Patient Involvement Specialist, Aberdeenshire, UK; 4 Leeds Institute for Clinical Trials Research (LICTR), School of Medicine, University of Leeds, Leeds, UK; 5 Independent Scholar, Epsom, UK; 6 Aberdeen Centre for Evaluation, University of Aberdeen, Aberdeen, UK; 7 Department of Women’s and Children’s Health, Uppsala University, Uppsala, Sweden

**Keywords:** Socioeconomic disadvantage, Inclusivity, Trials methodology, Underserved groups, Underrepresented groups, Trials, Conduct, Design

## Abstract

**Background:**

People experiencing socioeconomic disadvantage are persistently underrepresented in clinical trials, yet they experience a significantly greater burden of disease than those not experiencing socioeconomic disadvantage. Trials need to be inclusive to ensure that treatments are safe and effective for those who need them most. Resources are needed to support researchers in designing and implementing trials that are inclusive of people experiencing socioeconomic disadvantage. Building on the National Institute for Health and Care Research (NIHR) INCLUDE initiative, we developed the INCLUDE Socioeconomic Disadvantage Framework to support researchers in making trials more inclusive.

**Methods:**

The Framework was developed over five phases: (1) outlining an initial draft of the Framework, (2) refining the initial draft Framework with public contributors, (3) refining the draft Framework with wider contributors, (4) finalising the Framework with all contributors, and (5) launch and application of the Framework.

**Results:**

The Framework entails four key questions: (1) Who should my trial results apply to? (2) Are people from different socioeconomic backgrounds likely to respond to the intervention in different ways? (3) Will my trial intervention and/or comparator make it harder for people from different socioeconomic backgrounds to take part in the trial? (4) Will the way I have planned and designed my trial make it harder for people from different socioeconomic backgrounds to take part? The Framework includes worksheets to support trial teams in considering strategies to address barriers to inclusion. In 2023, the Framework was launched at a webinar with ~300 registrants and is currently available to download from the Trial Forge website: https://www.trialforge.org/trial-diversity/socioeconomic-disadvantage-framework/. Public contributor considerations, collated through project meetings, to make trials more inclusive for people experiencing socioeconomic disadvantage are also appendaged to this article to support trial teams further.

**Conclusion:**

The Framework and public contributor considerations can be used to support researchers to design and conduct more inclusive trials. Future work should include evaluation of such Frameworks, further engagement with funders to increase uptake, and development and evaluation of strategies to improve inclusion.

**Supplementary Information:**

The online version contains supplementary material available at 10.1186/s13063-026-09448-2.

## Background

In the past, researchers have not attracted a sufficiently diverse range of participants to clinical trials [1]. It is essential for clinical trials to be accessible to ensure trial findings are applicable to the target population [2], reduce the perpetuation of health inequalities [3], reduce the financial costs of underrepresentation [4], and enhance public trust in research [5]. Major barriers to the inclusion of underserved groups include suboptimal language and communication; lack of trust; limited access to trials; narrow eligibility criteria; attitudes and beliefs; lack of knowledge around trials, and logistical and practical issues [6].

Major research funders across the world have acknowledged that more work is needed to improve equity, diversity and inclusion in clinical research [1, 7, 8]. Funders, such as the US National Institutes of Health (NIH), the Canadian Institutes of Health Research (CIHR), and the National Institute for Health and Care Research (NIHR) in the UK, have therefore taken steps to improve access to clinical trials for people who are persistently underrepresented. Some efforts have included implementing policies for trialists to comply with when submitting funding applications (e.g. NIH’s Inclusion of Women and Members of Racial and/or Ethnic Minority Groups in Clinical Research) [9] and devising strategies to make research funding more equitable to applicants from less privileged groups (e.g. CIHR’s Strategic Plan 2021–2031) [8]. In the UK, the NIHR initiated a project called ‘Innovations in Clinical Trial Design and Delivery for the Under-served’ (INCLUDE) [10] in 2017. INCLUDE aims to identify barriers and drivers to inclusive research, as well as facilitate opportunities to optimise trial design and conduct [2].

In 2018, Trial Forge (an initiative to improve the efficiency of trials [https://www.trialforge.org]) came together with The Medical Research Council (MRC) Hubs for Trials Methodology Research Recruitment and Retention Working Group (now part of the MRC-NIHR Trial Methodology Research Partnership) to explore opportunities to improve representation of underserved groups in clinical trials, with a focus on ethnicity. They began to develop the INCLUDE Ethnicity Framework in 2019, eventually providing researchers with a tool to improve inclusion of people from marginalised ethnic backgrounds in clinical research [11]. Since then, the INCLUDE Impaired Capacity to Consent Framework has also been developed and launched [12] to support researchers in considering optimal ways to improve the inclusion of adults with impaired capacity to consent in research.

The NIHR identified ‘people experiencing socioeconomic disadvantage’ as another persistently underserved group in research [10]. Although there is contention as to how socioeconomic disadvantage should be defined, common attributes include lower levels of education, social class and income [13]. We shaped our definition of socioeconomic disadvantage as the project progressed, in response to consultation discussions. Closely linked, the Joseph Rowntree Foundation defines ‘poverty’ as ‘when a person’s resources (mainly their material resources) are not sufficient to meet their minimum needs (including social participation)’ [14]. More than 1 in 5 people in the UK (21%) were found to be in poverty in 2022/23 [15], and various hits to living standards that have affected the whole population in England, such as COVID-19 and the cost-of-living crisis, have exacerbated existing health inequalities [16]. This illustrates how wide-reaching and complex the issue of socioeconomic disadvantage is in the UK.

People experiencing socioeconomic disadvantage experience a higher burden of disease and early mortality than patients who are not experiencing socioeconomic disadvantage [17–20], yet they are underrepresented in clinical research and are less likely to be retained in research studies [21–23]. They are persistently underserved in clinical research [21, 22]. As there were no existing resources to support researchers in making trials more inclusive to people experiencing socioeconomic disadvantage, the aim of this project was to develop the INCLUDE Socioeconomic Disadvantage Framework to address this need.

## Developing the INCLUDE Socioeconomic Disadvantage Framework

**Fig. 1:**
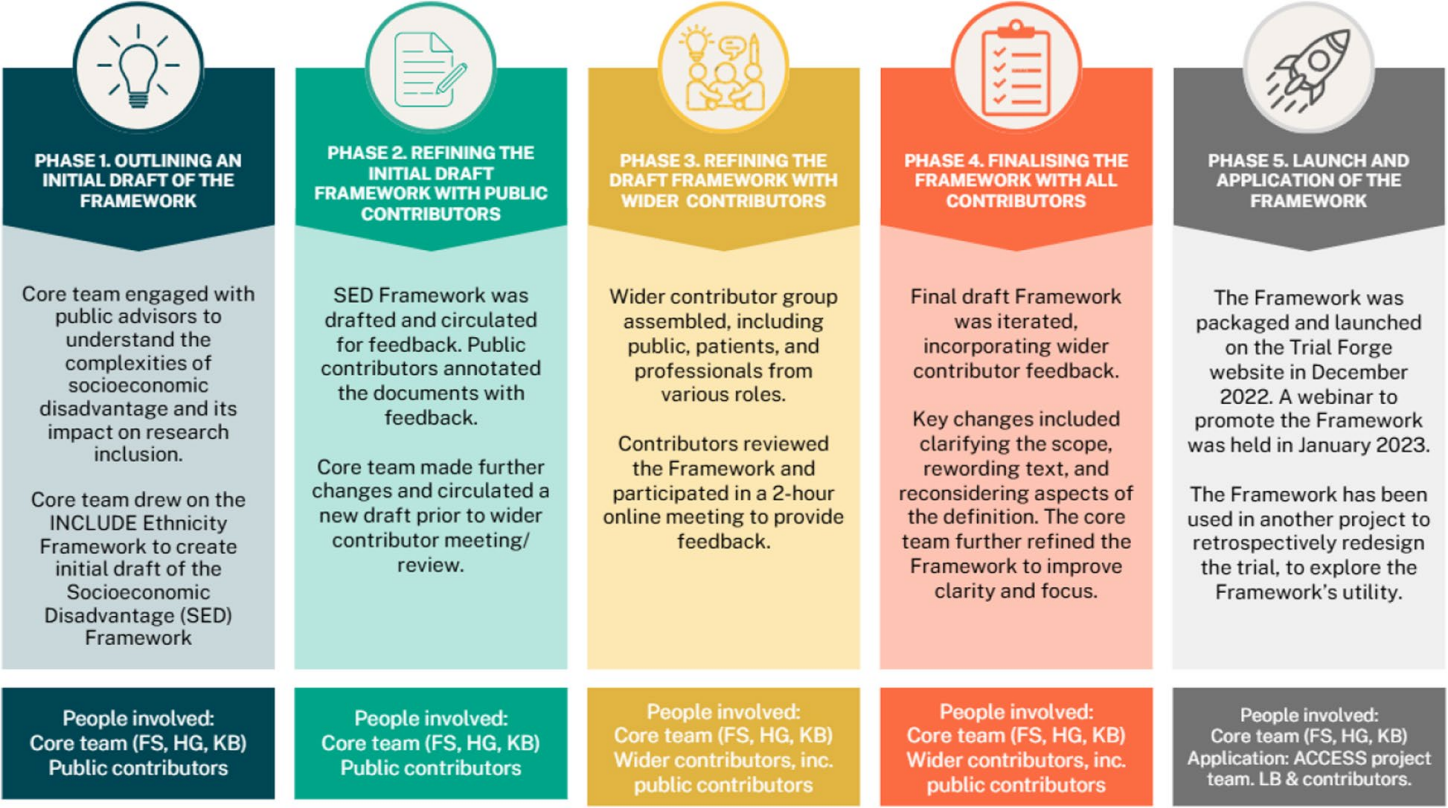
Development phases of the INCLUDE socioeconomic disadvantage framework

Framework development was led by members of the Inclusivity sub-group of the MRC-NIHR Trials Methodology Research Partnership (TMRP) Trial Conduct Working Group in the UK. It was developed in partnership with key contributors from relevant specialities (e.g. research, methodologists and funders) and public contributors who identified themselves as having experienced socioeconomic disadvantage. The Framework development process was guided by the approach and lessons learned from the development of the INCLUDE Ethnicity Framework [11]. Figure 1 illustrates the key five development phases: (1) outlining an initial draft of the Framework; (2) refining the initial draft Framework with public contributors; (3) refining the draft Framework with wider contributors; (4) finalising the Framework with all contributors; and (5) launch and application of the Framework.

### Phase 1: outlining an initial draft of the framework

We began with a core project team of researchers (KB, HG and FS), with extensive experience in trials methodology and conduct. We held several online meetings to discuss the scope of the Framework, to consider the feasibility of adapting the INCLUDE Ethnicity Framework [11], and to define a plan for the Framework development process. The core project team assembled a patient and public contributor group of six people to inform the development of the Framework throughout. We advertised the opportunity to contribute via the NIHR Applied Research Collaboration (ARC) North West Coast mailing list and NIHR’s People in Research website. We emphasised that we were keen to involve public contributors who had experience of socioeconomic disadvantage, stating that we were keen to involve ‘people living in low-income areas or individuals whose main income is from government benefits’. Public contributors received payments to recognise and reward their involvement, in line with the NIHR public contributor payment policy [24]. All meetings with public contributors were online, facilitated by members of the core project team, and entailed members of the core team taking comprehensive notes and/or recording the sessions to ensure that we had accurately captured key points.

In April 2021, we met with public contributors as a full project team for the first time. We discussed the aim of the project, definitions and perceptions of socioeconomic disadvantage, and key barriers to research participant and involvement for those experiencing socioeconomic disadvantage. Members of the core team communicated their interpretations of the meaning of ‘socioeconomic disadvantage’ and we asked public contributors to describe how they would define it, what their experience had been of it, and how they observe it. During the 2-h discussion, public contributors described how socioeconomic disadvantage can be challenging to define and measure, particularly as a person’s socioeconomic status can change throughout the life course. They described the dynamic nature of socioeconomic status as an important distinction compared to many other patient characteristics such as ethnicity, which are generally much more static. Contributors presented a long and varied list of barriers to inclusion for people experiencing socioeconomic disadvantage. KB and FS took notes during the meeting, which were combined following the session.

Following the meeting with public contributors, the core team met again to consider how best to define socioeconomic disadvantage for the Framework documentation (including example indicators) based on the discussions held. We also labelled and categorised the barriers to inclusion that had been identified during the discussion with contributors. We explored how socioeconomic disadvantage had been defined and operationalised in the literature, looking for concepts that aligned with the discussion held with public contributors. We established that there was no consensus on a definition of socioeconomic disadvantage, but the term is often used to refer to people living in less favourable social and economic conditions than most other people in the same society [25]. Although there is a lack of agreement as to how socioeconomic disadvantage should be operationalised, common attributes included education, social class and income [13].

HG reviewed practical ways to understand and communicate how socioeconomic disadvantage can manifest and presented the project team with a concept called ‘The 3 P’s’, informed by the UK Government’s Child Poverty Strategy 2014–2017 [26] and associated consultation work linked to the strategy [27]. The core team and public contributors agreed to adopt this concept in developing the Framework. We grouped indicator examples of socioeconomic disadvantage under one of three headings: (1) Pockets: indicators closely linked with income and economic resource availability; (2) Prospects: indicators closely linked with wellbeing and life chances; (3) Place: indicators closely linked with housing and local environment.

We used the INCLUDE Ethnicity Framework [11] as a template to develop the initial version of the INCLUDE Socioeconomic Disadvantage Framework, ensuring that we incorporated the scope of the new Framework, our definition of socioeconomic disadvantage and illustrations on how it can manifest. In response to contributors’ feedback, we made adaptations to the four key questions and worksheets that were part of the INCLUDE Ethnicity Framework, before presenting it to public contributors.

### Phase 2: refining the initial draft framework with public contributors

In advance of another one-hour meeting with the core project team and public contributors in June 2021, an initial draft Framework was circulated to the group (Additional file 1). Public contributors were encouraged to provide their views on all elements of the Framework. Again, members of the core project team noted considerations and potential adaptations suggested by the contributors. Contributors forwarded annotated paper/electronic draft Framework documents, whereby they highlighted text that needed further clarification, rewording, and suggested additions to text, particularly in relation to example barriers to inclusion and indicators of socioeconomic disadvantage. For example, we added introductory text to contextualise socioeconomic disadvantage in trials and provided examples of characteristics of socioeconomic disadvantage, which were also categorised. The core project team made further changes to the Framework and circulated a new draft of the Framework to public contributors (Additional file 2) in preparation for the third phase of development, which entailed presenting the Framework to wider contributors and obtaining feedback.

### Phase 3: refining the draft framework with wider contributors

A wider group of contributors was assembled to inform the development of the Framework. The group included members of the PPI group, established in Phase 1. We identified wider contributors with a variety of professional backgrounds to join the wider group, including academics with an interest in socioeconomic disadvantage, funders, trials methodologists, clinical trial unit managers, trial coordinators/managers, health professionals, linguists, and ethicists. The core project team populated a spreadsheet with: (1) personal contacts from the various professional domains, and (2) individuals who expressed an interest in taking part after the project was presented at a meeting for the Inclusivity sub-group of the NIHR-MRC Trials Methodology Research Partnership Conduct Working Group in September 2021. Twenty-five of the 36 identified professionals agreed to join the wider group of contributors. Table 1 lists all contributors’ roles and key points linked to their contributions over the project. Ultimately, we achieved representation from all identified specialties, except for those who primarily identified as linguists and ethicists.

**Table 1:** Roles and contribution timelines for those who agreed to join wider group of contributors

		**Apr 2021**	**Jun 2021**	**Jun 2021**	**Sep 2021**	**Oct 2021**	**Nov 2021**	**Apr 2022**
	Role	Meeting	Iteration circulated (Additional file 1)	Meeting	Iteration circulated (Additional file 2)	Iteration circulated (Additional file 3)	Meeting	Iteration circulated (Additional file 4)
1	Public contributor	X	X	X	X	X	X	X
2	Public contributor	X	X	X	X	X	X	X
3	Public contributor	X	X	X	X	X	X	X
4	Public contributor	X	X	X	X	X	X	X
5	Public contributor	X	X	X	X	X	X	X
6	Public contributor	X	X	X	X	X		X
7	Academic					X	X	X
8	Academic					X	X	X
9	Methodologist					X	X	X
10	Linguist/academic					X	X	X
11	Funder					X	X	X
12	Funder					X	X	X
13	PPI expert					X	X	X
14	Clinician					X		X
15	Clinician					X	X	X
16	Methodologist					X	X	X
17	Research nurse					X		X
18	Research nurse					X		X
19	Academic					X	X	X
20	Academic					X	X	X
21	Clinician					X		X
22	Trial manager/PPI Lead					X	X	X
23	CTU Director					X	X	X
24	CTU Director					X	X	X
25	Diversity Expert (Charity)					X	X	X
26	Deputy Lead, CTU					X	X	X
27	PPI Expert					X		X
28	Trial coordinator					X	X	X
29	Methodologist					X		X
30	Research nurse					X		X
31	Clinician					X		X

*PPI* patient public involvement, *CTU* clinical trials unit

In October 2021, all contributors were sent an electronic or written copy of the most recent iteration of the Framework (Additional file 3) in advance of the meeting with the wider group of contributors. Three wider contributors offered written comments on the draft in advance of the meeting in November 2021, which were also integrated when making further changes to the Framework. The online 2-h meeting was held with 22 wider contributors, including 5 public contributors. FS and KB facilitated the group and both took notes. The meeting was also recorded to sense-check and expand the notes taken following the meeting. Following introductions, FS explained to the group that the aim of the meeting was to establish contributors’ views on the draft of the Framework to inform further refinement. FS and KB shared the initial draft of the Framework on screen and facilitated discussions about all components of the Framework, in the order they appeared: (1) our definition of socioeconomic status; (2) key questions; (3) worksheets. We invited contributors to offer their views on each component, as well as identifying areas for improvement or missing components.

### Phase 4. finalising the framework with all contributors

In April 2022, a final draft Framework was developed (Additional file 4) and circulated to the wider group, which integrated notes taken by KB and FS during the meeting held in November 2021 and a video of the meeting. Several key changes that had been made included clarifying the scope of the Framework, combining aspects of sections, rewording sections, and reconsidering elements of the operational definition of socioeconomic disadvantage. Contributors also described how examples of strategies to address identified barriers to inclusion would be useful. Although producing recommended strategies to address barriers to inclusion was outside of the scope of developing the Framework, KB and FS amalgamated and refined notes taken from the meetings with contributors to produce public contributor considerations to making trials more inclusive to people experiencing socioeconomic disadvantage (Additional file 5).

The final draft Framework (Additional file 4) was circulated to all contributors, and three wider contributors responded with remaining comments, mainly acknowledging the value of the work that was done. After integrating final small changes to the draft, we circulated the finalised Framework to all contributors in September 2022.

### Phase 5: launch and application of the framework

The Framework was packaged and launched on the Trial Forge website in December 2022 (see Additional file 6). HG, KB and FS hosted a webinar to launch the Framework, which was held on 24th January 2023, with ~300 registrants.

Guided by the development process of The INCLUDE Ethnicity Framework [11], we applied the INCLUDE Socioeconomic Disadvantage Framework to some real-world research projects to consider how the Framework might be used by trialists. Table 2 provides a breakdown of two projects, how the Frameworks were applied, and researcher reflections in each case: (1) The REEACT-2 trial (ISRCTN55310481) [28], and (2) a proposal named Diabetic Foot Ulcer REsearch PlatFORM (DFU-REFORM).

**Table 2:** Example application of the INCLUDE Socioeconomic Disadvantage Framework with two projects and associated reflections

Project name	The REEACT-2 trial	DFU-REFORM
Project topic	Comparing the clinical effectiveness and cost-effectiveness of telephone-facilitated free-to-use computerised cognitive behavioural therapy (cCBT) with minimally supported cCBT	Design a multi-disciplinary adaptive platform of studies for diabetes-related foot ulcer (DFU) to improve DFU healing, reduce serious outcomes and provide rapid and efficient delivery of research answering questions of importance to the NHS
When was it applied?	September 2022	September 2024
Retrospectively/prospectively applied	Retrospective	Prospective (pre-funding)
Who led the application of the Framework?	KB and ACCESS^b^ research assistant (KH, see acknowledgements)	LB
How was it applied?^a^	1. KB and KH drafted the Framework worksheets with a wider contributor group prior to meeting a group of public contributors. KB and KH produced a document (to present to public contributors) detailing the trial design and recommendations generated from completing the worksheets 2. KB and KH hosted a meeting with public contributors, splitting discussion into topics focused on trial concepts (e.g. target population and recruitment setting) 3. KB incorporated all relevant discussion points raised by public contributors into the drafted Framework	1. Establish the link between socioeconomic status and diabetes and DFUs by conducting a supplementary literature review, combined with knowledge acquired from developing the Accelerator Award 2. LB completed draft Framework to initiate discussion 3. Draft Framework worksheets presented at a management group meeting 4. New iteration of the draft Framework worksheets presented at a second management group meeting to refine 5. Framework worksheets presented at Patient Advisory Group (PAG) meetings. Feedback incorporated into a new iteration(s) 6. PAG attended a further meeting, where they made further suggestions
Reflections on applying the Framework	- Thorough completion would have needed more time - Some discussion points were relevant to multiple Framework sections, resulting in repetition - KB acknowledged that retrospectively applying the Framework is not how it is intended to be used, but this was the first application. There were gaps in knowledge about how elements of the trial (e.g. PPI) had been conducted, which impacted Framework completion	- Incorporated perspectives from management group and patient advisory group (PAG), but both groups required support in doing so - The PAG viewed completing the Framework as an acceptable PPI activity - Time was costed for completion of the Framework, which was beneficial, but it was challenging to incorporate all views and complete in time despite this - LB and colleagues have since started to develop more research proposals, using the Framework and seek to diversify the PAG, which has partly been inspired by completing the Framework

^a^ Additional file 7 provides the example application of the Framework for this project, including completed worksheets

^b^ ACCESS Study [29]

## Discussion

The INCLUDE Socioeconomic Disadvantage Framework has been designed to aid researchers, who are designing clinical trials, to consider barriers to including people experiencing socioeconomic disadvantage in their trial. It can also help researchers to develop strategies to attempt to address such barriers, with the aim of making trials more inclusive.

The Framework is presented as an editable (Microsoft Word) document, prompting researchers to respond to four key questions to consider who they intend to include in their trial, whether such patients might respond to a trial intervention in different ways, whether the trial intervention and/or comparator make it more difficult for such patients to participate in the trial, and whether the way the trial is planned and designed could make it harder for such patients to take part. Worksheets have been created that are linked to key questions 2–4, encouraging researchers to answer the questions and consider the actions needed to address the identified barriers to inclusion. The worksheets refer to the 3 P’s concept described earlier, which is intended to prompt researchers to think about the breadth of possible issues that people experiencing socioeconomic disadvantage may encounter when presented with a research opportunity. The Framework, alongside other INCLUDE Frameworks, contributes to achieving the aims of the NIHR INCLUDE Roadmap [2] to better support researchers in making their research more inclusive.

The Framework can be used as well as the INCLUDE Ethnicity Framework [11] and the INCLUDE Impaired Capacity to Consent Framework [12], depending on the intended target population of the trial being designed. We acknowledge that completing several Frameworks could be time-consuming when developing grant applications and/or setting up trials [30]. The examples of Framework application in this paper show the effort required and difficulty achieving one complete Framework, even with funded time for completion. Although completing individual Frameworks to identify and address barriers to inclusion is a progressive step towards making trials more equitable, having separate Frameworks for individual underserved groups fails to incorporate an intersectional approach. In reality, a person’s multiple social identities (e.g. being a person from an ethnic minority background who is experiencing socioeconomic disadvantage) can intersect to generate combined disadvantages that are greater than the sum of their individual parts [31]. Future work needs to consider how best to develop and refine existing and future resources to support researchers in making trials inclusive, whilst considering if and how underserved groups can intersect in ways that may exacerbate barriers to inclusion [32].

Current research is ongoing to explore opportunities to consider how best to design and implement resources and strategies to support researchers in making trials more inclusive, including exploring trialists’ views and experiences of current resources (including the INCLUDE Frameworks) to promote inclusivity in trials [33] and exploring the use of artificial intelligence (AI) to aid completion of the INCLUDE Frameworks (led by LB). In addition to the INCLUDE Frameworks, the Trial Forge website (https://www.trialforge.org/) is home to additional resources designed to support researchers in making trials inclusive, such as guidance to consider sex and gender, older people, and intersectionality. Where possible, the INCLUDE Socioeconomic Disadvantage Framework should be evaluated further to establish its effectiveness in improving trials’ inclusivity.

A priority for future research in this field is to develop and test practical strategies to overcome barriers to making trials more inclusive to people experiencing socioeconomic disadvantage. In this project, wider contributors repeatedly highlighted the lack of evidence and the need for further guidance despite their willingness to implement strategies to improve inclusion. The lack of evidence to support evidence-informed inclusive trial design decisions remains a significant challenge, and not just for socioeconomic disadvantage. In the absence of evidence-based recommendations, Additional file 5 in this paper offers researchers pointers, informed by public contributors on this project, to make clinical research more accessible to people experiencing socioeconomic disadvantage.

### Strengths and weaknesses

The core team and wider contributor group (including public contributors) were all based in the UK and the Framework was developed in the UK. Early discussions with contributors explored the scope of the Framework and whether it could or should be applied when developing trials outside of the UK. The core team and wider contributor group recognised that barriers to including people experiencing socioeconomic disadvantage in trials may differ between trials conducted in a high-income country, such as the UK, and lower-and middle-income countries. We agreed that although further work would be needed to explore whether the Framework could be effectively applied in multi-country trials or trials conducted in lower- and middle-income countries, the Framework could still be applied outside of the UK as aspects of it may still be relevant. Similarly, we discussed whether the Framework could be applied in the planning of research designs that did not entail a clinical trial. Again, whilst further work is needed to explore the Framework’s application in contexts outside of trials, the core elements of the Framework have relevance to the design of research studies more broadly.

In Table 1, we described the roles and contributions of everyone involved in developing the Framework. We did not collect information on contributors’ sociodemographic characteristics, since this work entailed consultation, rather than research. However, future projects developing resources to improve trials inclusivity may wish to collect information linked to contributors’ sociodemographic characteristics. Providing further context of contributors’ background or positionality may ensure a transparent approach and enhance the quality of the resource being developed [34, 35].

## Conclusions

People experiencing socioeconomic disadvantage are persistently underserved in clinical research. These groups are underrepresented in clinical research despite experiencing a much greater burden of disease compared with those who are not experiencing socioeconomic disadvantage. Clinical trials need to be more inclusive to ensure that treatments and interventions are safe and effective in real-world contexts for these groups. The development of this, and other INCLUDE Frameworks, seeks to improve the representativeness of trial populations by providing researchers with resources to support them in considering barriers to inclusion and identifying strategies to address such barriers.

This report, which details the development of the INCLUDE Socioeconomic Disadvantage Framework, may offer guidance to researchers looking to develop future resources that aim to make research more inclusive. Future research should now focus on (1) developing and evaluating strategies to make research more inclusive for people experiencing socioeconomic disadvantage (including this Framework); (2) increasing uptake of the INCLUDE Frameworks by working closely with funders; and (3) finding more efficient ways to complete the relevant frameworks to maximise impact but minimise potential time burden. It is also crucial to consider how people may belong to intersecting underserved groups and the exacerbating negative effect this can have on research inclusion.

## Supplementary Information

Additional file 1.

Additional file 2.

Additional file 3.

Additional file 4.

Additional file 5.

Additional file 6.

Additional file 7.

## Data Availability

The INCLUDE Socioeconomic Disadvantage Framework and accompanying resources are available online at: https://www.trialforge.org/trial-diversity/socioeconomic-disadvantage-framework/.

## Abbreviations

NIH: National Institutes of Health
CIHR: Canadian Institutes of Health Research
NIHR: National Institute for Health and Care Research
INCLUDE: Innovations in Clinical Trial Design and Delivery for the Under-served
MRC: Medical Research Council
TMRP: Trials Methodology Research Recruitment
PPI: Patient and public involvement
DFU-REFORM: Diabetic Foot Ulcer REsearch PlatFORM
DFU: Diabetes-related foot ulcer
PPIE: Patient and public involvement and engagement
PAG: Patient Advisory Group
cCBT: Computerised cognitive behavioural therapy
REEACT-2: Telephone-supported computerised cognitive-behavioural therapy randomised controlled trial
