# Supplementary material for: Making trials more inclusive of people experiencing socioeconomic disadvantage: developing the INCLUDE socioeconomic disadvantage framework
Source: Trials. 2026 Jan 14;27:123. doi: 10.1186/s13063-026-09448-2 (PMC12888448; doi:10.1186/s13063-026-09448-2)
Supplement: Supplementary file 1 — Additional file 1. [file 13063_2026_9448_MOESM1_ESM.docx]

**Additional file 1 Framework Iteration 1: June 2021**

**Making research more accessible to patients and members of the public experiencing socio-economic disadvantage**

Trial teams need to do everything possible to make their trial relevant to the people to whom the results are intended to apply (often patients) and those expected to apply them (often healthcare professionals). The four questions below are intended to prompt trial teams to think about who should be involved as participants, and how to facilitate their involvement as much as possible. These questions should be considered by trial teams in partnership with patient and public partners, including individuals from, or representing, groups identified in Question 1.

Note that:

- *‘Intervention*’ means the treatment, initiative or service being evaluated.
- ‘*Comparator*’ means the what the intervention is being compared to.
- ‘*Effective*’ means the intervention provides important benefits for people with the disease or condition that is the focus of the trial.

We recommend that trial teams use the worksheets to help them think through their answers to the four Key Questions.

1. **Who should my trial results apply to?**

Which groups in my community could benefit from the intervention if it was found effective, or benefit from not having it if it was found ineffective and/or harmful?

1. **Are the groups identified in Question 1 likely to respond to the treatment in different ways?**

How much the disease or cultural factors mean that some groups in the community respond to, or engage with, the treatment(s) being tested in different ways?

1. **Will my trial intervention and/or comparator make it harder for any of the groups identified in Question 1 to engage with the intervention and/or comparator?**

How might the intervention and/or comparator, including how they are provided, make it harder for some groups in the community to take part in the trial?

1. **Will the way I have planned and designed my trial make it harder for any of the groups identified in Question 1 to consider taking part?**

How might elements of trial design, such as eligibility criteria or the recruitment and consent process, make it harder for some groups in the community to take part?

**Worksheets for thinking through factors that might affect involvement of socioeconomically disadvantaged groups in a trial**

These worksheets are intended to be used by trial teams in partnership with patient and public partners to ensure that socioeconomically disadvantaged group involvement is considered at the trial design stage. Before completing the worksheets, the trial team should have answered Question 1 of the INCLUDE Key Questions about socioeconomically disadvantaged group involvement.

The worksheet may cover issues that some trial teams already think about. The intention is that the worksheet will help to highlight issues consistently across trials for all trial teams, as well as raising some questions that may not be routinely considered at present.

While the worksheet asks trial teams to think about possible differences between socioeconomically disadvantaged groups, it is important to remember that there are also differences *within* socioeconomically disadvantaged groups, especially between generations and between men and women. No group is homogenous.

Rather than attempting to identify people, we encourage trial teams to focus on the accessibility of their research with people experiencing socioeconomic disadvantage in mind.

We encourage trial teams to consider using the 3Ps; Pockets – income and resource availability, Prospects – expectations and life chances, and Places – housing and the local environment, as a starting point when completing the worksheets. See Appendix 1 for more information on our definition of socioeconomic disadvantage, and where the 3Ps have been used elsewhere.

**Worksheet 1**

This worksheet provides some questions to guide your thinking about involvement of people experiencing socioeconomic disadvantage when answering Question 2 of the INCLUDE Key Questions.

| **Disease and cultural factors that might influence the effect of treatment for some socioeconomically disadvantaged groups** | | |
| --- | --- | --- |
| Disease | How might the prevalence of the disease vary between people experiencing socioeconomic disadvantage in the target population? | Response:  Pockets:  Prospects:  Places:  Other factors: |
|  | How might the severity of the disease vary between people experiencing socioeconomic disadvantage? | Response:  Pockets:  Prospects:  Places:  Other factors: |
|  | How might presentation of the disease vary between people experiencing socioeconomic disadvantage (this may include symptoms, type or pattern or rate of disease progression)? | Response:  Pockets:  Prospects:  Places:  Other factors: |
|  | How close is the match between the socioeconomically disadvantaged groups in the target population, and the people living in the areas where the trial is to be run? | Response:  Pockets:  Prospects:  Places:  Other factors: |
|  | Other factors to consider: | |
| Socio-economic | How might perceptions of the disease and social stigma around it vary between people experiencing socioeconomic disadvantage and those who are not? | Response:  Pockets:  Prospects:  Places:  Other factors: |
|  | How might ways of describing the disease vary between people experiencing socioeconomic disadvantage? | Response:  Pockets:  Prospects:  Places:  Other factors: |
|  | How might cultural practices, beliefs and traditions influence the acceptability of, and adherence to, the treatment(s) for people experiencing socioeconomic disadvantage? | Response:  Pockets:  Prospects:  Places:  Other factors: |
|  | How or when might people experiencing socioeconomic disadvantage access healthcare for this disease differently? | Response:  Pockets:  Prospects:  Places:  Other factors: |
|  | Other factors to consider: | |

**Worksheet 2**

This worksheet provides some questions to guide your thinking about involvement of people experiencing socioeconomic disadvantage when answering Question 3 of the INCLUDE Key Questions.

| **Intervention and comparator factors that might affect how some groups engage with the intervention and/or comparator*** | | |
| --- | --- | --- |
| What | How much the intervention(s) and comparator limit participation of people experiencing socioeconomic disadvantage? | Response:  Pockets:  Prospects:  Places:  Other factors: |
|  | How, and in what way, were people experiencing socioeconomic disadvantage involved in selecting or designing the trial intervention/comparator? | Response:  Pockets:  Prospects:  Places:  Other factors: |
|  | Other factors to consider: | |
| Who | How might the person delivering the intervention/comparator limit participation of people experiencing socioeconomic disadvantage? | Response:  Pockets:  Prospects:  Places:  Other factors: |
|  | Other factors to consider: | |
| How | How might the mode of delivery (e.g., telephone, video-call, face-to-face, in groups) limit participation of people experiencing socioeconomic disadvantage? | Response:  Pockets:  Prospects:  Places:  Other factors: |
|  | Other factors to consider: | |
| When | How might where the intervention/comparator is delivered (e.g., hospital, general practice, local library) limit the participation of people experiencing socioeconomic disadvantage? | Response:  Pockets:  Prospects:  Places:  Other factors: |
|  | Other factors to consider: |  |
| When and intensity | How might when the intervention/comparator is delivered (e.g., during working hours) or the intensity (e.g., number of times it is delivered, over what period, time commitment for each session and overall) limit participation of people experiencing socioeconomic disadvantage? | Response:  Pockets:  Prospects:  Places:  Other factors: |
|  | Other factors to consider: | |

*These factors are taken from TIDieR ([http://www.equator-network.org/reporting-guidelines/tidier/](about:blank)).

**Worksheet 3a**

This worksheet provides some questions to guide your thinking about involvement of people experiencing socioeconomic disadvantage when answering Question 4 of the INCLUDE Key Questions.

| **Trial eligibility and participation factors that might affect how some groups engage with the trial** | | |
| --- | --- | --- |
| Eligibility | How might eligibility criteria exclude people experiencing socioeconomic disadvantage for reasons other than their clinical eligibility for the trial (e.g., availability of medical history, language requirements, location, gender, age, discussing pregnancy, internet/mobile telephone access)? | Response:  Pockets:  Prospects:  Places:  Other factors: |
|  | Other factors to consider: | |
| Opportunity to participate | How might the way(s) (and by whom) potential participants are made aware of the trial (e.g., posters in a clinic, written letter from a doctor, asked by a nurse) limit the participation of people experiencing socioeconomic disadvantage? | Response:  Pockets:  Prospects:  Places:  Other factors: |
|  | Other factors to consider: | |
|  | How might the information that tells potential participants about the trial (e.g., participant information leaflet) limit the participation of people experiencing socioeconomic disadvantage? | Response:  Pockets:  Prospects:  Places:  Other factors: |
|  | How might cultural practices, beliefs and traditions change the way that people experiencing socioeconomic disadvantage perceive the information they are given? | Response:  Pockets:  Prospects:  Places:  Other factors: |
|  | Other factors to consider: | |
| Consent procedures | How might the way consent is sought (i.e., where, by whom, written vs verbal, verbal translations/multiple languages, access to interpreters) limit the participation of people experiencing socioeconomic disadvantage? | Response:  Pockets:  Prospects:  Places:  Other factors: |
|  | How might the way people would like to discuss participation with family before providing consent differ for people experiencing socioeconomic disadvantage? | Response:  Pockets:  Prospects:  Places:  Other factors: |
|  | How might the way the research team can check how well consent information is understood differ for people experiencing socioeconomic disadvantage? | Response:  Pockets:  Prospects:  Places:  Other factors: |
|  | Other factors to consider: | |

**Worksheet 3b**

This worksheet provides some questions to guide your thinking about involvement of people experiencing socioeconomic disadvantage when answering Question 4 of the INCLUDE Key Questions.

| **Trial data collection factors that might affect how some groups engage with the trial** | | |
| --- | --- | --- |
| What | How, and in what way, were people experiencing socioeconomic disadvantage involved in selecting the trial outcomes? | Response:  Pockets:  Prospects:  Places:  Other factors: |
|  | How might the trial outcomes themselves, or other data being collected (e.g., a patient’s background information) limit the participation of people experiencing socioeconomic disadvantage? | Response:  Pockets:  Prospects:  Places:  Other factors: |
|  | Other factors to consider: | |
| Who | How might the people who collect data limit the participation of people experiencing socioeconomic disadvantage? | Response:  Pockets:  Prospects:  Places:  Other factors: |
|  | Other factors to consider: | |
| How | How might data collection methods limit the participation of people experiencing socioeconomic disadvantage? | Response:  Pockets:  Prospects:  Places:  Other factors: |
|  | Other factors to consider: | |
| Where | How might where data are collected limit the participation of people experiencing socioeconomic disadvantage? | Response:  Pockets:  Prospects:  Places:  Other factors: |
|  | Other factors to consider: | |

**Worksheet 3c**

This worksheet provides some questions to guide your thinking about involvement of people experiencing socioeconomic disadvantage when answering Question 4 of the INCLUDE Key Questions.

| **Factors that might affect the planned analysis of trial results** | | |
| --- | --- | --- |
| Retention | How might the trial data available for participants differ between socioeconomic groups in the target population? | Response:  Pockets:  Prospects:  Places:  Other factors: |
|  | Other factors to consider: | |
| Benefits | How might the benefits of the trial intervention(s) differ between socioeconomic groups in the target population? | Response:  Pockets:  Prospects:  Places:  Other factors: |
|  | Other factors to consider: | |
| Harms | How might the possible harms of the trial intervention(s) differ between socioeconomic groups in the target population? | Response:  Pockets:  Prospects:  Places:  Other factors: |
|  | Other factors to consider: | |
| Subgroup analyses | How should variation between socioeconomic groups in the target population be explored– should there be planned subgroup analyses? | Response:  Pockets:  Prospects:  Places:  Other factors: |
|  | Other factors to consider: | |
| Interim analyses | How should any interim analysis handle variation between socioeconomic groups in the target population? | Response:  Pockets:  Prospects:  Places:  Other factors: |
|  | Other factors to consider | |
| Stopping triggers | How should any rules to stop the trial early on safety or benefit grounds handle variation between socioeconomic groups in the target population? | Response:  Pockets:  Prospects:  Places:  Other factors: |
|  | Other factors to consider: | |

**Worksheet 3d**

This worksheet provides some questions to guide your thinking about involvement of people experiencing socioeconomic disadvantage when answering Question 4 of the INCLUDE Key Questions.

| **Factors that might affect the planned reporting and dissemination of trial results** | | |
| --- | --- | --- |
| What | How, and in what way, were people experiencing socioeconomic disadvantage involved in planning the reporting and dissemination of the trial results? | Response:  Pockets:  Prospects:  Places:  Other factors: |
|  | Other factors to consider: | |
| How | How might planned reporting and dissemination methods limit engagement with people experiencing socioeconomic disadvantage? | Response:  Pockets:  Prospects:  Places:  Other factors: |
|  | Other factors to consider: | |
| Where | How might where trial results are planned to be reported and disseminated limit engagement of people experiencing socioeconomic disadvantage? | Response:  Pockets:  Prospects:  Places:  Other factors: |
|  | Other factors to consider: | |

**Worksheet for thinking through measures to address factors that might prevent full community involvement**

Use this worksheet to list key factor that might affect the involvement of some socioeconomically disadvantaged groups in the target population of your trial, along with measures to mitigate the effect of those factors and their cost. Add extra rows as needed.

Please remember that there are also differences *within* socioeconomic groups, especially between generations and between men and women. No group is homogenous.

| **Factors that may prevent full community involvement** | **Proposed measures (several options may be needed)** | **Cost of measures** |
| --- | --- | --- |
|  |  |  |
|  |  |  |
|  |  |  |
|  |  |  |
|  |  |  |
|  |  |  |
|  |  |  |
|  |  |  |
|  |  |  |
|  |  |  |
|  |  |  |
|  |  |  |

**Appendix 1**

**Defining socioeconomic disadvantage**

We are aware that the language and terminology used to describe socioeconomic disadvantage can be sensitive. We would welcome any feedback and suggestions that you may have at: [info@trialforge.org](mailto:info@trialforge.org)

As a team, we struggled to find a detailed definition of what being ‘socioeconomically disadvantaged’ involved. In general terms, the phrase refers to people living in less favourable social and economic circumstances than others in the same society, but there are many different factors that can contribute to people finding themselves in this situation, and different ways to interpret the term ‘society’ (e.g., the global society, a country, entire cities or regions, or specific areas within them).

Factors that are known to contribute to socioeconomic disadvantage can be categorised as the ‘3Ps’; Pockets, Prospects and Places, describing income and resource availability, expectations and life chances, and housing and the local environment, respectively. The 3Ps were listed as shorthand for the three overarching target outcomes in the UK government’s Child Poverty Strategy 2014-2017.

We encourage trial teams to think about these 3 factors as a minimum. The 3Ps can play out as insecure or lack of employment, low educational attainment and reduced literacy levels, reliance on state benefits and/or reduced financial security, living in a council-owned property, use of food banks, and homelessness. Socioeconomic disadvantage can be dynamic; situations are not necessarily permanent, and events can change socioeconomic status and the experiences that go along with that quickly, for better or worse.

Research has shown that other aspects of identity that are known to result in societal inequalities intersect with socioeconomic status, which results in people from minority ethnic groups, people experiencing physical and/or learning disabilities, people living with mental ill health, people from the LGBTQIA+ community, and women, being at a higher risk of experience socioeconomic disadvantage. We encourage trial teams to think carefully about where socioeconomic status intersects with these experiences and implement facilitators and alleviate barriers accordingly.

Ultimately, socioeconomic disadvantage is more than low income, and describes the impact of a complex multidimensional problem that encompasses the social injustices and inequalities that contribute to further inequalities for people in our society that are already at their most vulnerable.

It is important to note that socioeconomic disadvantage is *not* something that you can see. It is dangerous to make assumptions about people’s backgrounds or experiences, and rather than attempting to identify people, we encourage trial teams to focus on the accessibility of their research with people experiencing socioeconomic disadvantage in mind. Simplifying processes, building trust, and reducing logistical barriers, will encourage everyone to consider trial participation, improving representation and engagement with people experiencing socioeconomic disadvantage too.

**Appendix 2**

**How (and by who) was the INCLUDE Socioeconomic Framework developed?**

The National Institute for Health Research (NIHR) initiated the INCLUDE initiative in 2017. The Medical Research Council (MRC) Hubs for Trials Methodology Research Recruitment and Retention Working Group was at the same time starting efforts to improve representation within trials, particularly of black, Asian and minority ethnic individuals. The two groups came together in late 2018 to develop a research grant proposal for work on inclusion in trials. That grant originally included what has become the INCLUDE Ethnicity Framework, but the groups decided to develop the tool outside the grant in early 2019. Work on the INCLUDE Ethnicity Framework began in earnest in July 2019, and the complete Framework was launched in October 2020.

In June 2019 the Medical Research Council (MRC) Hubs for Trials Methodology Research became part of the MRC-NIHR Trials Methodology Research Partnership (TMRP). The Trial Conduct TMRP working group established the Inclusivity sub-group, which had its first meeting in July 2020, and based on discussions at this meeting, work began on the INCLUDE Socioeconomic Framework in November 2020.

1. Developing an outline of what was needed

| **Purpose:** | | |
| --- | --- | --- |
| **Participants** | | |
| **Name** | **Affiliation** | **Perspective** |
| Heidi Gardner |  |  |
| Fran Sherratt |  |  |
| Katie Biggs |  |  |
|  |  |  |
|  |  |  |
|  |  |  |

1. Developing an initial draft of the Framework

| **Purpose:** | | |
| --- | --- | --- |
| **Participants** | | |
| **Name** | **Affiliation** | **Perspective** |
| Heidi Gardner |  |  |
| Fran Sherratt |  |  |
| Katie Biggs |  |  |
|  |  |  |
|  |  |  |
|  |  |  |

1. Discussing that draft with a wider stakeholder group

| **Purpose:** | | |
| --- | --- | --- |
| **Participants** | | |
| **Name** | **Affiliation** | **Perspective** |
| Heidi Gardner |  |  |
| Fran Sherratt |  |  |
| Katie Biggs |  |  |
|  |  |  |
|  |  |  |
|  |  |  |

1. Modifying the draft based on feedback from stakeholders

| **Purpose:** | | |
| --- | --- | --- |
| **Participants** | | |
| **Name** | **Affiliation** | **Perspective** |
| Heidi Gardner |  |  |
| Fran Sherratt |  |  |
| Katie Biggs |  |  |
|  |  |  |
|  |  |  |
|  |  |  |

1. Stakeholder feedback on the modified draft

| **Purpose:** | | |
| --- | --- | --- |
| **Participants** | | |
| **Name** | **Affiliation** | **Perspective** |
| Heidi Gardner |  |  |
| Fran Sherratt |  |  |
| Katie Biggs |  |  |
|  |  |  |
|  |  |  |
|  |  |  |

1. Applying the Framework
2. Packaging the Framework, examples, and other materials
