# Supplementary material for: Making trials more inclusive of people experiencing socioeconomic disadvantage: developing the INCLUDE socioeconomic disadvantage framework
Source: Trials. 2026 Jan 14;27:123. doi: 10.1186/s13063-026-09448-2 (PMC12888448; doi:10.1186/s13063-026-09448-2)
Supplement: Supplementary file 2 — Additional file 2. [file 13063_2026_9448_MOESM2_ESM.docx]

**Additional file 2 Framework Iteration 2. September 2021.**

**Making research more accessible to patients and members of the public experiencing socio-economic disadvantage**

**Defining socio-economic disadvantage**

As a team, we struggled to find a detailed definition of what being ‘socioeconomically disadvantaged’ involves. Socioeconomic disadvantage can be dynamic; situations are not necessarily permanent, and events can change socioeconomic status and the experiences that go along with that quickly, for better or worse. We are aware that the language and terminology used to describe socioeconomic disadvantage can be sensitive. We would welcome any feedback and suggestions that you may have at: [info@trialforge.org](mailto:info@trialforge.org)

In general terms, *socioeconomically disadvantaged* refers to people living in less favourable social and economic circumstances than others in the same society, but there are many different factors that can contribute to people finding themselves in this situation, and different ways to interpret the term ‘society’ (e.g., the global society, a country, entire cities or regions, or specific areas within them).

Factors that are known to contribute to socioeconomic disadvantage can be categorised as the ‘3Ps’; Pockets, Prospects, and Places, describing income and resource availability, expectations and life chances, and housing and the local environment, respectively. The 3Ps are adapted from the three overarching target outcomes in the UK government’s Child Poverty Strategy 2014-2017. Table 1 illustrates how example factors associated with socio-economic disadvantage fit within each of the 3Ps. We encourage researchers to consider the 3Ps as a minimum when designing a study.

**Table 1.** The 3Ps and associated example characteristics of socio-economic disadvantage

|  | **Example factors** |
| --- | --- |
| **Pockets**  *Income and resource availability* | - Being unable to work (e.g., as a result of disability, chronic illness, or other reasons) - Insecure employment or being unemployed - Reliance on state benefits and/or reduced financial security - Use of food banks - Less ‘spare’ time/high work or caring responsibilities - Limited or no access to the Internet or computer equipment - Feeling powerless or vulnerable due to limited income or material resources. |
| **Prospects**  *Expectations and life chances* | - Lower educational attainment and literacy levels - Unable to attend higher education - Perceived power hierarchy between doctors and patients, which play into feelings of mistrust of research or healthcare systems - Acceptance of ‘how it is’ - Low self-confidence or belief - Poorer access to accurate and reliable information about health and research, which also plays into feelings of mistrust of research or healthcare systems. |
| **Places**  *Housing and the local environment* | - Living in a council-owned property - People experiencing homelessness - Access to suitable housing - Being part of a traveller community - People in prison - Being an immigrant or refugee - Access community services - Less engagement with NHS services |

Research has shown that other aspects of identity that are known to result in societal inequalities intersect with socioeconomic status, which results in people from minoritised ethnic groups, people experiencing physical and/or learning disabilities, people living with mental ill health, people from the LGBTQIA+ community, and women, being at a higher risk of experience socioeconomic disadvantage. We encourage trial teams to think carefully about where socioeconomic status intersects with these experiences, and work to implement facilitators and alleviate barriers accordingly.

Ultimately, socioeconomic disadvantage is more than low income, it’s the combined outcome of various situations and experiences, and it can change over time. It describes the impact of a complex multidimensional problem that encompasses the social injustices and inequalities that contribute to further inequalities for people in our society that are already at their most vulnerable.

It is important to note that socioeconomic disadvantage is *not* something that you can see. It is dangerous to make assumptions about people’s backgrounds or experiences, and rather than attempting to identify people, we encourage research teams to focus on the accessibility of their research with people experiencing socioeconomic disadvantage in mind. Simplifying processes, building trust, working with patients and public contributors from such backgrounds, and reducing logistical barriers, will encourage everyone to consider trial participation, improving representation and engagement with people experiencing socioeconomic disadvantage too. Where possible, we encourage researchers to work with people with lived experience of socioeconomic disadvantage, involving and embedding individuals within research teams to ensure that trials are designed collaboratively.

**Key questions**

This document is designed to inspire research teams to do everything possible to make their research relevant to the people that the results are likely to impact (often patients) and those expected to apply them (often healthcare professionals). The four questions below are intended to prompt research teams to think about who should be involved as participants, and how to facilitate their involvement as much as possible. These questions should be considered by research teams in partnership with patient and public partners, including individuals from, or representing, groups identified in Question 1.

Note that:

- *‘Intervention*’ means the treatment, initiative or service being evaluated.
- ‘*Comparator*’ means what the intervention is being compared to.
- ‘*Effective*’ means the intervention provides important benefits for people with the disease or condition that is the focus of the research.

We recommend that research teams use the worksheets to help them think through their answers to the four Key Questions.

1. **Who should my trial results apply to?**

Which people could benefit from the intervention if it is found to be effective, or benefit from not having it if it is found to be ineffective and/or harmful? Are there any groups routinely omitted from research in your population or disproportionately affected by the condition or disease?

1. **Are the people identified in Question 1 likely to respond to the treatment in different ways?**

Could socio-economic factors influence the way the people identified in question one might respond to, or engage with, the treatment(s) being tested? How might income and resource availability, expectations and life chances, and housing and the local environment affect how people will respond to, or engage with their condition or treatment?

1. **Will my trial intervention and/or comparator make it harder for any of the people identified in Question 1 to engage with the intervention and/or comparator?**

How might the intervention and/or comparator, including how they are delivered, make it harder for some people in the community to take part in the trial? How might income and resource availability, expectations and life chances, and housing and the local environment make it harder for some people to access or accept the intervention or comparator?

1. **Will the way I have planned and designed my research make it harder for any of the people identified in Question 1 to consider taking part?**

How might elements of research design, such as eligibility criteria or the recruitment and consent process, make it harder for some people in the community to take part?

**Worksheets for thinking through factors that might affect research participation of people experiencing socio-economic disadvantage**

The four worksheets are intended to be used by research teams in partnership with patient and public partners to ensure that people experiencing socio-economic disadvantage are considered at the research design stage.

The worksheets may cover issues that some research teams already think about. The intention is that the worksheets will help to highlight issues consistently across research for all clinical research teams, as well as raising some questions that may not be routinely considered at present.

Rather than attempting to identify people, we encourage trial teams to focus on the accessibility of their research with people experiencing socioeconomic disadvantage in mind.

We encourage research teams to consider using the 3Ps; Pockets – income and resource availability, Prospects – expectations and life chances, and Places – housing and the local environment, as a starting point when completing the worksheets. See Table 1 for more information on our definition of socioeconomic disadvantage, and where the 3Ps have been used elsewhere.

**Before completing the worksheets,** **you should have answered Question 1 of the INCLUDE Key Questions about engaging people experiencing socio-economic disadvantage.**

**Worksheet 1**

**Before completing this worksheet,** **you should have answered Question 1 of the INCLUDE Key Questions about engaging people experiencing socio-economic disadvantage.**

This worksheet includes questions to guide your thinking about involvement of people experiencing socioeconomic disadvantage when answering Question 2 of the INCLUDE Key Questions.

Factors that are known to contribute to socioeconomic disadvantage can be categorised as the ‘3Ps’; Pockets, Prospects, and Places, describing income and resource availability, expectations and life chances, and housing and the local environment, respectively. For examples, see Table 1.

| **Socio-economic factors that might influence the effect of treatment for some groups** | | |
| --- | --- | --- |
| Health condition | How might the prevalence of the health condition vary between people experiencing socioeconomic disadvantage in the target population? | Response:  Pockets:  Prospects:  Places:  Other factors: |
|  | How might the severity of the health condition vary between people experiencing socioeconomic disadvantage? | Response:  Pockets:  Prospects:  Places:  Other factors: |
|  | How might presentation of the health condition vary between people experiencing socioeconomic disadvantage (this may include symptoms, type or pattern or rate of disease progression)? | Response:  Pockets:  Prospects:  Places:  Other factors: |
|  | How close is the match between the socioeconomically disadvantaged groups in the target population, and the people living in the areas where the trial is to be run? | Response:  Pockets:  Prospects:  Places:  Other factors: |
|  | Other factors to consider: | |
| Cultural factors | How might perceptions of the health condition and social stigma around it vary between people experiencing socioeconomic disadvantage and those who are not? | Response:  Pockets:  Prospects:  Places:  Other factors: |
|  | How might ways of describing the disease vary between people experiencing socioeconomic disadvantage? | Response:  Pockets:  Prospects:  Places:  Other factors: |
|  | How might participant income and resource availability, expectations, and local environment influence the acceptability of, and adherence to, the treatment(s) for people experiencing socioeconomic disadvantage? | Response:  Pockets:  Prospects:  Places:  Other factors: |
|  | How or when might people experiencing socioeconomic disadvantage access healthcare for this disease differently to those who are not? | Response:  Pockets:  Prospects:  Places:  Other factors: |
|  | Other factors to consider: | |

**Worksheet 2**

This worksheet provides some questions to guide your thinking about involvement of people experiencing socioeconomic disadvantage when answering Question 3 of the INCLUDE Key Questions.

**Before completing this worksheet,** **you should have answered Question 1 of the INCLUDE Key Questions about engaging people experiencing socio-economic disadvantage.**

Factors that are known to contribute to socioeconomic disadvantage can be categorised as the ‘3Ps’; Pockets, Prospects, and Places, describing income and resource availability, expectations and life chances, and housing and the local environment, respectively. For examples, see Table 1.

| **Intervention and comparator factors that might affect how some groups engage with the intervention and/or comparator*** | | |
| --- | --- | --- |
| What | How much the intervention(s) and comparator limit participation of people experiencing socioeconomic disadvantage? | Response:  Pockets:  Prospects:  Places:  Other factors: |
|  | How, and in what way, were people experiencing socioeconomic disadvantage involved in selecting or designing the trial intervention/comparator? | Response:  Pockets:  Prospects:  Places:  Other factors: |
|  | Other factors to consider: | |
| Who | How might the person delivering the intervention/comparator limit participation of people experiencing socioeconomic disadvantage? | Response:  Pockets:  Prospects:  Places:  Other factors: |
|  | Other factors to consider: | |
| How | How might the mode of delivery (e.g. telephone, video-call, face-to-face, in groups) limit participation of people experiencing socioeconomic disadvantage? | Response:  Pockets:  Prospects:  Places:  Other factors: |
|  | Other factors to consider: | |
| When | How might where the intervention/comparator is delivered (e.g. hospital, general practice, local library) limit the participation of people experiencing socioeconomic disadvantage? | Response:  Pockets:  Prospects:  Places:  Other factors: |
|  | Other factors to consider: |  |
| When and intensity | How might when the intervention/comparator is delivered (e.g. during working hours) or the intensity (e.g. number of times it is delivered, over what period, time commitment for each session and overall) limit participation of people experiencing socioeconomic disadvantage? | Response:  Pockets:  Prospects:  Places:  Other factors: |
|  | Other factors to consider: | |

*These factors are taken from TIDieR ([http://www.equator-network.org/reporting-guidelines/tidier/](about:blank)).

**Worksheet 3a**

This worksheet provides some questions to guide your thinking about involvement of people experiencing socioeconomic disadvantage when answering Question 4 of the INCLUDE Key Questions.

**Before completing this worksheet,** **you should have answered Question 1 of the INCLUDE Key Questions about engaging people experiencing socio-economic disadvantage.**

Factors that are known to contribute to socioeconomic disadvantage can be categorised as the ‘3Ps’; Pockets, Prospects, and Places, describing income and resource availability, expectations and life chances, and housing and the local environment, respectively. For examples, see Table 1.

| **Trial eligibility and participation factors that might affect how some groups engage with the trial** | | |
| --- | --- | --- |
| Eligibility | How might eligibility criteria exclude people experiencing socioeconomic disadvantage for reasons other than their clinical eligibility for the trial (e.g. availability of medical history, language requirements, location, gender, age, discussing pregnancy, internet/mobile telephone access)? | Response:  Pockets:  Prospects:  Places:  Other factors: |
|  | Other factors to consider: | |
| Opportunity to participate | How might the way(s) (and by whom) potential participants are made aware of the trial (e.g. posters in a clinic, written letter from a doctor, who approaches the patient about the trial) limit the participation of people experiencing socioeconomic disadvantage? | Response:  Pockets:  Prospects:  Places:  Other factors: |
|  | Other factors to consider: | |
|  | How might the mode and format of the information that tells potential participants about the trial (e.g. participant information leaflet, online, video) limit the participation of people experiencing socioeconomic disadvantage? | Response:  Pockets:  Prospects:  Places:  Other factors: |
|  | How might cultural practices, beliefs and traditions change the way that people experiencing socioeconomic disadvantage perceive the information they are given? | Response:  Pockets:  Prospects:  Places:  Other factors: |
|  | Other factors to consider: | |
| Consent procedures | How might the way consent is sought (i.e. where, by whom, written vs verbal, verbal translations/multiple languages, access to interpreters) limit the participation of people experiencing socioeconomic disadvantage? | Response:  Pockets:  Prospects:  Places:  Other factors: |
|  | How might the way people would discuss participation with family, friends or others outside of the research team before providing consent differ for people experiencing socioeconomic disadvantage? | Response:  Pockets:  Prospects:  Places:  Other factors: |
|  | How might the way the research team can check how well consent information is understood differ for people experiencing socioeconomic disadvantage? | Response:  Pockets:  Prospects:  Places:  Other factors: |
|  | Other factors to consider: | |

**Worksheet 3b**

This worksheet provides some questions to guide your thinking about involvement of people experiencing socioeconomic disadvantage when answering Question 4 of the INCLUDE Key Questions.

**Before completing this worksheet,** **you should have answered Question 1 of the INCLUDE Key Questions about engaging people experiencing socio-economic disadvantage.**

Factors that are known to contribute to socioeconomic disadvantage can be categorised as the ‘3Ps’; Pockets, Prospects, and Places, describing income and resource availability, expectations and life chances, and housing and the local environment, respectively. For examples, see Table 1.

| **Trial data collection factors that might affect how some groups engage with the trial** | | |
| --- | --- | --- |
| What | How, and in what way, were people experiencing socioeconomic disadvantage involved in selecting the trial outcomes? | Response:  Pockets:  Prospects:  Places:  Other factors: |
|  | How might the trial outcomes themselves, or other data being collected (e.g. a patient’s background information) limit the participation of people experiencing socioeconomic disadvantage? | Response:  Pockets:  Prospects:  Places:  Other factors: |
|  | Other factors to consider: | |
| Who | How might the people who collect data limit the participation of people experiencing socioeconomic disadvantage (e.g. role, power dynamics, relationship)? | Response:  Pockets:  Prospects:  Places:  Other factors: |
|  | Other factors to consider: | |
| How | How might data collection methods (e.g. ) limit the participation of people experiencing socioeconomic disadvantage? | Response:  Pockets:  Prospects:  Places:  Other factors: |
|  | Other factors to consider: | |
| Where | How might the location where trial data are collected limit participation of people experiencing socioeconomic disadvantage (e.g. poor transport links, don’t drive, feeling uncomfortable in setting)? | Response:  Pockets:  Prospects:  Places:  Other factors: |
|  | Other factors to consider: | |

**Worksheet 3c**

This worksheet provides some questions to guide your thinking about involvement of people experiencing socioeconomic disadvantage when answering Question 4 of the INCLUDE Key Questions.

**Before completing this worksheet,** **you should have answered Question 1 of the INCLUDE Key Questions about engaging people experiencing socio-economic disadvantage.**

Factors that are known to contribute to socioeconomic disadvantage can be categorised as the ‘3Ps’; Pockets, Prospects, and Places, describing income and resource availability, expectations and life chances, and housing and the local environment, respectively. For examples, see Table 1.

| **Factors that might affect the planned analysis of trial results** | | |
| --- | --- | --- |
| Retention | How might the accuracy and completeness of trial data collected differ between socioeconomic groups in the target population? | Response:  Pockets:  Prospects:  Places:  Other factors: |
|  | Other factors to consider: | |
| Benefits | How might the benefits of the trial intervention(s) differ between socioeconomic groups in the target population? | Response:  Pockets:  Prospects:  Places:  Other factors: |
|  | Other factors to consider: | |
| Harms | How might the possible harms of the trial intervention(s) differ between socioeconomic groups in the target population? | Response:  Pockets:  Prospects:  Places:  Other factors: |
|  | Other factors to consider: | |
| Subgroup analyses | How should variation between socioeconomic groups in the target population be explored– should there be planned subgroup analyses? | Response:  Pockets:  Prospects:  Places:  Other factors: |
|  | Other factors to consider: | |
| Interim analyses | How should any interim analysis handle variation between socioeconomic groups in the target population? | Response:  Pockets:  Prospects:  Places:  Other factors: |
|  | Other factors to consider | |
| Stopping triggers | How should any rules to stop the trial early on safety or benefit grounds handle variation between socioeconomic groups in the target population? | Response:  Pockets:  Prospects:  Places:  Other factors: |
|  | Other factors to consider: | |

**Worksheet 3d**

This worksheet provides some questions to guide your thinking about involvement of people experiencing socioeconomic disadvantage when answering Question 4 of the INCLUDE Key Questions.

**Before completing this worksheet,** **you should have answered Question 1 of the INCLUDE Key Questions about engaging people experiencing socio-economic disadvantage.**

Factors that are known to contribute to socioeconomic disadvantage can be categorised as the ‘3Ps’; Pockets, Prospects, and Places, describing income and resource availability, expectations and life chances, and housing and the local environment, respectively. For examples, see Table 1.

| **Factors that might affect the planned reporting and dissemination of trial results** | | |
| --- | --- | --- |
| What | How, and in what way, were people experiencing socioeconomic disadvantage involved in planning the reporting and dissemination of the trial results? | Response:  Pockets:  Prospects:  Places:  Other factors: |
|  | Other factors to consider: | |
| How | How might planned reporting and dissemination methods limit engagement with people experiencing socioeconomic disadvantage? | Response:  Pockets:  Prospects:  Places:  Other factors: |
|  | Other factors to consider: | |
| Where | How might your results dissemination strategy limit engagement of people experiencing socioeconomic disadvantage? | Response:  Pockets:  Prospects:  Places:  Other factors: |
|  | Other factors to consider: | |

**Worksheet X: Measures to address the identified factors that might prevent full community research participation**

Use this worksheet to summarise the key factors you have identified that could prevent people experiencing socio-economic disadvantage from fully participating in the research, along with measures to mitigate the effect of those factors and their cost. Add extra rows as needed.

Factors that are known to contribute to socioeconomic disadvantage can be categorised as the ‘3Ps’; Pockets, Prospects, and Places, describing income and resource availability, expectations and life chances, and housing and the local environment, respectively. For examples, see Table 1.

| **Factors that may prevent full community participation** | **Proposed measures (several options may be needed)** | **Cost of measures** |
| --- | --- | --- |
|  |  |  |
|  |  |  |
|  |  |  |
|  |  |  |
|  |  |  |
|  |  |  |
|  |  |  |
|  |  |  |
|  |  |  |
|  |  |  |
|  |  |  |

**Appendix 1**

**How (and by who) was the INCLUDE Socioeconomic Framework developed?**

The National Institute for Health Research (NIHR) initiated the INCLUDE initiative in 2017. The Medical Research Council (MRC) Hubs for Trials Methodology Research Recruitment and Retention Working Group was at the same time starting efforts to improve representation within trials, particularly of black, Asian and minority ethnic individuals.

The two groups came together in late 2018 to develop a research grant proposal for work on inclusion in trials. That grant originally included what has become the INCLUDE Ethnicity Framework, but the groups decided to develop the tool outside the grant in early 2019. Work on the INCLUDE Ethnicity Framework began in earnest in July 2019, and the complete Framework was launched in October 2020.

In June 2019 the Medical Research Council (MRC) Hubs for Trials Methodology Research became part of the MRC-NIHR Trials Methodology Research Partnership (TMRP). The Trial Conduct TMRP working group established the Inclusivity sub-group, which had its first meeting in July 2020, and based on discussions at this meeting, work began on the INCLUDE Socioeconomic Framework in November 2020.

1. Developing an outline of what was needed

| **Purpose:** | | |
| --- | --- | --- |
| **Participants** | | |
| **Name** | **Affiliation** | **Perspective** |
| Heidi Gardner |  |  |
| Fran Sherratt |  |  |
| Katie Biggs |  |  |
|  |  |  |
|  |  |  |
|  |  |  |

1. Developing an initial draft of the Framework

| **Purpose:** | | |
| --- | --- | --- |
| **Participants** | | |
| **Name** | **Affiliation** | **Perspective** |
| Heidi Gardner |  |  |
| Fran Sherratt |  |  |
| Katie Biggs |  |  |
|  |  |  |
|  |  |  |
|  |  |  |

1. Discussing that draft with a wider stakeholder group

| **Purpose:** | | |
| --- | --- | --- |
| **Participants** | | |
| **Name** | **Affiliation** | **Perspective** |
| Heidi Gardner |  |  |
| Fran Sherratt |  |  |
| Katie Biggs |  |  |
|  |  |  |
|  |  |  |
|  |  |  |

1. Modifying the draft based on feedback from stakeholders

| **Purpose:** | | |
| --- | --- | --- |
| **Participants** | | |
| **Name** | **Affiliation** | **Perspective** |
| Heidi Gardner |  |  |
| Fran Sherratt |  |  |
| Katie Biggs |  |  |
|  |  |  |
|  |  |  |
|  |  |  |

1. Stakeholder feedback on the modified draft

| **Purpose:** | | |
| --- | --- | --- |
| **Participants** | | |
| **Name** | **Affiliation** | **Perspective** |
| Heidi Gardner |  |  |
| Fran Sherratt |  |  |
| Katie Biggs |  |  |
|  |  |  |
|  |  |  |
|  |  |  |

1. Applying the Framework
2. Packaging the Framework, examples, and other materials
