# Supplementary material for: Making trials more inclusive of people experiencing socioeconomic disadvantage: developing the INCLUDE socioeconomic disadvantage framework
Source: Trials. 2026 Jan 14;27:123. doi: 10.1186/s13063-026-09448-2 (PMC12888448; doi:10.1186/s13063-026-09448-2)
Supplement: Supplementary file 3 — Additional file 3. [file 13063_2026_9448_MOESM3_ESM.docx]

Additional file 3. Framework Iteration 3. October 2021

Making clinical research more accessible to individuals experiencing socio-economic disadvantage

**Defining socio-economic disadvantage**

As a team, we struggled to find a detailed definition of what being ‘socioeconomically disadvantaged’ involves. Socioeconomic disadvantage can be dynamic; situations are not necessarily permanent, and events can change socioeconomic status and the experiences that go along with that quickly, for better or worse. We are aware that the language and terminology used to describe socioeconomic disadvantage can be sensitive. We would welcome any feedback and suggestions that you may have at: [info@trialforge.org](mailto:info@trialforge.org)

In general terms, *socioeconomically disadvantaged* refers to people living in less favourable social and economic circumstances than others in the same society, but there are many different factors that can contribute to people finding themselves in this situation, and

different ways to interpret the term ‘society’ (e.g., the global society, a country, entire cities or regions, or specific areas within them).

Factors that are known to contribute to socioeconomic disadvantage can be categorised as the ‘3Ps’; Pockets, Prospects, and Places describing income and resource availability, expectations and life chances, and housing and the local environment, respectively. The 3Ps are adapted from the three overarching target outcomes in the UK government’s Child Poverty Strategy 2014-2017. Table 1 illustrates how example factors associated with socio- economic disadvantage fit within each of the 3Ps. We encourage researchers to consider the 3Ps as a minimum when designing a study.

Research has shown that other aspects of identity that are known to result in societal inequalities intersect with socioeconomic status, which results in people from minoritised ethnic groups, people experiencing physical and/or learning disabilities, people living with mental ill-health, people from the LGBTQIA+ community, and women, being at a higher risk of experiencing socioeconomic disadvantage. We encourage trial teams to think carefully about where socioeconomic status intersects with these experiences, and work to implement facilitators and alleviate barriers accordingly.

Ultimately, socioeconomic disadvantage is more than low income, it’s the combined outcome of various situations and experiences, and it can change over time. It describes the impact of a complex multidimensional problem that encompasses the social injustices and inequalities that contribute to further inequalities for people in our society that are already at their most vulnerable.

It is important to note that socioeconomic disadvantage is *not* something that you can see. It is dangerous to make assumptions about people’s backgrounds or experiences, and rather than attempting to identify people, we encourage research teams to focus on the accessibility of their research with people experiencing socioeconomic disadvantage in mind. Simplifying processes, building trust, working with patients and public contributors from such backgrounds, and reducing logistical barriers, will encourage everyone to consider trial participation, improving representation and engagement with people

experiencing socioeconomic disadvantage too. Where possible, we encourage researchers to work with people with lived experiences of socioeconomic disadvantage, involving and embedding individuals within research teams throughout the research lifecycle to ensure that trials are designed collaboratively.

**Table 1.** The 3Ps and associated example characteristics of socio-economic disadvantage

|  | **Example factors** |
| --- | --- |
| **Pockets** *Income and resource availability* | - Being unable to work (e.g., as a result of disability, chronic illness, or other reasons) - Insecure employment or being unemployed - Reliance on state benefits and/or reduced financial security - Use of food banks - Less ‘spare’ time/high work or caring responsibilities - Limited or no access to the Internet, computers or mobile devices. - Feeling powerless or vulnerable due to limited income or material resources. - Covert situations within relationships (e.g. financial abuse) |
| **Prospects** *Expectations and life chances* | - Lower educational attainment and literacy levels - Unable to attend higher education - Perceived power hierarchy between doctors and patients, which play into feelings of mistrust of research or healthcare systems - Acceptance of ‘how it is’ - Low self-confidence, belief or motivation - Poorer access to accurate and reliable information about health and research, which also plays into feelings of mistrust of research or healthcare systems. |
| **Places**  *Housing and the local environment* | - Living in unacceptable or poor housing conditions - People experiencing homelessness - Being part of a traveller community - People in prison - Being an immigrant or refugee - Limited mobility and access to transport systems - Access to community services - Less engagement with NHS services |

**INCLUDE Key Questions**

This document is designed to inspire research teams to do everything possible to make their research relevant to the people that the results are likely to impact (often patients) and those expected to apply them (often healthcare professionals). The four questions below are intended to prompt research teams to think about who should be involved as participants, and how to facilitate their involvement as much as possible. These questions should be considered by research teams in partnership with patient and public partners, including individuals from, or representing, groups identified in Question 1.

Note that:

- *‘Intervention*’ means the treatment, initiative or service being evaluated.
- ‘*Comparator*’ means what the intervention is being compared to.
- ‘*Effective*’ means the intervention provides important benefits for people with the

disease or condition that is the focus of the research.

**We recommend that research teams use the worksheets to help them think through their answers to the four Key Questions…**

**Q1. Who should my trial results apply to?**

Which people could benefit from the intervention if it is found to be effective, or benefit from not having it if it is found to be ineffective and/or harmful? Are there any groups routinely omitted from research in your population or disproportionately affected by the condition or disease?

**Q2. Are the people identified in Question 1 likely to respond to the treatment in different ways?**

Could socio-economic factors influence the way the people identified in question one might respond to, or engage with, the treatment(s) being tested? How might income and resource availability, expectations and life chances, and housing and the local environment affect how people will respond to, or engage with their condition or treatment?

**Q3. Will my trial intervention and/or comparator make it harder for any of the people identified in Question 1 to engage with the intervention and/or comparator?**

How might the intervention and/or comparator, including how they are delivered, make it harder for some people in the community to take part in the trial? How might income and resource availability, expectations and life chances, and housing and the local environment make it harder for some people to access or accept the intervention or comparator?

**Q4. Will the way I have planned and designed my research make it harder for any of the people identified in Question 1 to consider taking part?**

How might elements of research design, such as eligibility criteria or the recruitment and consent process, make it harder for some people in the community to take part?

**WORKSHEETS**

The four worksheets are intended to be used by research teams in partnership with patient and public partners to ensure that people experiencing socio-economic disadvantage are considered at the research design stage.

The worksheets may cover issues that some research teams already think about. The intention is that the worksheets will help to highlight issues consistently across research for all clinical research teams, as well as raising some questions that may not be routinely considered at present.

Rather than attempting to identify people, we encourage trial teams to focus on the accessibility of their research with people experiencing socioeconomic disadvantage in mind.

We encourage research teams to consider using the 3Ps; Pockets – income and resource availability, Prospects – expectations and life chances, and Places – housing and the local environment, as a starting point when completing the worksheets. See Table 1 for more information on our definition of socioeconomic disadvantage, and where the 3Ps have been used elsewhere.

**Before completing the worksheets, you should have answered Question 1 of the INCLUDE Key Questions…**

**Q1) Who should my trial results apply to?**

Which people could benefit from the intervention if it is found to be effective, or benefit from not having it if it is found to be ineffective and/or harmful? Are there any groups routinely omitted from research in your population or disproportionately affected by the condition or disease?

**WORKSHEET A: Are the people identified in Question 1 likely to respond to the treatment in different ways? (i.e. Q2 of the INCLUDE Key Questions)**

This worksheet includes questions to guide your thinking about the participation of people experiencing socioeconomic disadvantage when answering Question 2 of the INCLUDE Key Questions.

Factors that are known to contribute to socioeconomic disadvantage can be categorised as the ‘3Ps’; Pockets (income and resource availability), Prospects (expectations and life chances), and Places (housing and local environment). Please see Table 1 for examples.

|  | **Socio-economic factors that might influence the effectiveness of treatment for some groups** | | | |
| --- | --- | --- | --- | --- |
|  | **Pockets** | **Prospects** | **Places** | **Other(s)** |
| **Health condition** |  |  |  |  |
| How might the prevalence of the health condition vary between people experiencing socioeconomic disadvantage in the target  population? |  |  |  |  |
| How might the severity of the health condition vary between people experiencing socioeconomic  disadvantage? |  |  |  |  |
| How might the presentation of the health condition vary between people experiencing  socioeconomic |  |  |  |  |

| disadvantage (this may include symptoms, type or  pattern or rate of disease progression)? |  |  |  |  |
| --- | --- | --- | --- | --- |
| **Culture** |  |  |  |  |
| How might perceptions of the health condition and social stigma around it vary between people experiencing socioeconomic  disadvantage and those who are not? |  |  |  |  |
| How might ways of describing the disease vary between people experiencing socioeconomic  disadvantage? |  |  |  |  |
| How might the  participants’ circumstances influence the acceptability of, and adherence to, the treatment(s) for people experiencing socioeconomic  disadvantage? |  |  |  |  |
| How or when might  people experiencing |  |  |  |  |

| socioeconomic disadvantage access healthcare for this disease  differently to those who are not? |  |  |  |  |
| --- | --- | --- | --- | --- |

**WORKSHEET B: Will my trial intervention and/or comparator make it harder for any of the people identified in Question 1 to engage with the intervention and/or comparator? (i.e. Q3 of the INCLUDE Key Questions)**

This worksheet provides some questions to guide your thinking about participation of people experiencing socioeconomic disadvantage when answering Question 3 of the INCLUDE Key Questions.

Factors that are known to contribute to socioeconomic disadvantage can be categorised as the ‘3Ps’; Pockets (income and resource availability), Prospects (expectations and life chances), and Places (housing and local environment). Please see Table 1 for examples.

|  | **Intervention and comparator factors that might affect how participants from some socio-economic groups engage with the intervention and/or comparator*** | | | |
| --- | --- | --- | --- | --- |
|  | **Pockets** | **Prospects** | **Places** | **Other(s)** |
| **Who** |  |  |  |  |
| How might the person delivering the intervention/comparator limit participation of people experiencing socioeconomic  disadvantage? |  |  |  |  |
| **What** |  |  |  |  |
| How might the design/delivery of the intervention/comparator limit participation of people experiencing socioeconomic  disadvantage? |  |  |  |  |
| How, and in what ways, were people experiencing socioeconomic disadvantage  involved in selecting or |  |  |  |  |

| designing the trial intervention/comparator? |  |  |  |  |
| --- | --- | --- | --- | --- |
| **When** |  |  |  |  |
| How might when the intervention/comparator is delivered (e.g. during working hours) and/or the frequency/intensity it is delivered (e.g. number of times it is delivered, over what period, time commitment for each session and overall) limit participation of people experiencing socioeconomic  disadvantage? |  |  |  |  |
| **Where** |  |  |  |  |
| How might where the intervention/comparator is delivered (e.g. hospital, general practice, local community venues) limit the participation of people  experiencing socioeconomic disadvantage? |  |  |  |  |
| **How** |  |  |  |  |
| How might the mode of delivery (e.g. telephone, video-call, face-to-face, in  groups) limit the |  |  |  |  |

| participation of people  experiencing socioeconomic disadvantage? |  |  |  |  |
| --- | --- | --- | --- | --- |

*These factors are taken from TIDieR ([http://www.equator-network.org/reporting-guidelines/tidier/).](http://www.equator-network.org/reporting-guidelines/tidier/))

**WORKSHEET C: Will the way I have planned and designed my research make it harder for any of the people identified in Question 1 to consider taking part? (i.e. Q4 of the INCLUDE Key Questions)**

This four-part worksheet provides some questions to guide your thinking about participation of people experiencing socioeconomic disadvantage when answering Question 4 of the INCLUDE Key Questions. Worksheet C is divided into four sub-worksheets:

C.1: Trial eligibility and information C.2: Data collection

C.3: Data analysis

C.4: Reporting and dissemination

Factors that are known to contribute to socioeconomic disadvantage can be categorised as the ‘3Ps’; Pockets (income and resource availability), Prospects (expectations and life chances), and Places (housing and local environment). Please see Table 1 for examples.

**WORKSHEET C.1: Trial eligibility and accessibility factors that might affect how some groups engage with the trial**

|  | **Pockets** | **Prospects** | **Places** | **Other(s)** |
| --- | --- | --- | --- | --- |
| **Eligibility** |  |  |  |  |
| How might eligibility criteria exclude people experiencing socioeconomic disadvantage for reasons other than their clinical eligibility for the trial (e.g. availability of medical history, language requirements, location, gender, age, discussing pregnancy, internet/mobile  telephone access)? |  |  |  |  |
| **Trial information** |  |  |  |  |

| How might the way(s) (and by whom) potential participants are made aware of the trial (e.g. posters in a clinic, letterheaded paper in brown envelope that has negative associations for participant [e.g. debt agency], documents written in plain English, who approaches the participant about the trial) limit the participation of people  experiencing socioeconomic disadvantage? |  |  |  |  |
| --- | --- | --- | --- | --- |
| How might the mode and format of the information that tells potential participants about the trial (e.g. participant information leaflet, online, video) limit the participation of people experiencing socioeconomic  disadvantage? |  |  |  |  |
| How might potential participants’ cultural practices, beliefs and  traditions change the way that people experiencing socioeconomic disadvantage |  |  |  |  |

| perceive the information they are given? |  |  |  |  |
| --- | --- | --- | --- | --- |

**WORKSHEET C.2: Trial data collection factors that might affect how some groups engage with the trial**

|  | **Pockets** | **Prospects** | **Places** | **Other(s)** |
| --- | --- | --- | --- | --- |
| **Who** |  |  |  |  |
| How might the people who collect data limit the participation of people experiencing socioeconomic disadvantage (e.g. role, power dynamics,  relationship)? |  |  |  |  |
| **What** |  |  |  |  |
| How, and in what way, were people experiencing socioeconomic disadvantage involved in selecting the trial  outcomes? |  |  |  |  |
| How might the trial outcomes themselves, or other data being collected (e.g. participant’s background information) limit the participation of people experiencing socioeconomic  disadvantage? |  |  |  |  |
| **When** |  |  |  |  |

| How might when the data is collected (e.g. appointments conflicting with work or childcare responsibilities) and/or the frequency/duration it is collected (e.g. number of follow up appointments, length of questionnaires) limit participation of people  experiencing socioeconomic disadvantage? |  |  |  |  |
| --- | --- | --- | --- | --- |
| **Where** |  |  |  |  |
| How might the location where trial data are collected limit participation of people experiencing socioeconomic disadvantage (e.g. no/limited disposable income for additional public transport, don’t drive, poor transport links, feeling uncomfortable  in setting)? |  |  |  |  |
| **How?** |  |  |  |  |
| How might data collection methods (e.g. questionnaire format and length, additional clinical tests, mode of collection [e.g. option for  various modes such as app, |  |  |  |  |

| via telephone, paper questionnaire], type of remuneration and impact on government benefits i.e. voucher/cash) limit the participation of people  experiencing socioeconomic disadvantage? |  |  |  |  |
| --- | --- | --- | --- | --- |

**WORKSHEET C.3: Factors that might affect the analysis of trial results**

|  | **Pockets** | **Prospects** | **Places** | **Other(s)** |
| --- | --- | --- | --- | --- |
| **Representativeness** |  |  |  |  |
| How close is the match between the socioeconomically disadvantaged groups in the target population, and the  people living in the areas where the trial is to be run? |  |  |  |  |
| **Retention** |  |  |  |  |
| How might the accuracy and completeness of trial data collected differ between socio-economic groups in the target population (i.e. participants from the most  socio-economically disadvantaged backgrounds |  |  |  |  |

| compared to participants from the least)? |  |  |  |  |
| --- | --- | --- | --- | --- |
| **Intervention benefits** |  |  |  |  |
| How might the benefits of the trial intervention(s) differ between socioeconomic backgrounds in the target  population? |  |  |  |  |
| **Intervention harms** |  |  |  |  |
| How might the possible harms of the trial intervention(s) differ between socioeconomic groups in the target  population? |  |  |  |  |
| **Subgroup analyses** |  |  |  |  |
| How should variation between socioeconomic groups in the target population be explored– should there be planned  subgroup analyses? |  |  |  |  |
| **Interim analyses** |  |  |  |  |
| How should any interim analysis handle variation between socioeconomic groups in the target  population? |  |  |  |  |
| **Stopping triggers** |  |  |  |  |

| How should any rules to stop the trial early on intervention safety or intervention benefit grounds handle variation between socioeconomic  groups in the target population? |  |  |  |  |
| --- | --- | --- | --- | --- |

**WORKSHEET C.4: Factors that might affect the reporting and dissemination of trial results**

|  | **Pockets** | **Prospects** | **Places** | **Other(s)** |
| --- | --- | --- | --- | --- |
| **What** |  |  |  |  |
| How, and in what way, were people experiencing socioeconomic disadvantage involved in planning the reporting and dissemination  of the trial results? |  |  |  |  |
| **How** |  |  |  |  |
| How might your reporting and dissemination strategy limit engagement of people experiencing socioeconomic  disadvantage? |  |  |  |  |

**WORKSHEET D: What measures can I put in place to address the identified factors that might prevent optimal participations of the people identified in Q1?**

Use this worksheet to summarise the key factors you have identified that could prevent people experiencing socio-economic disadvantage from fully participating in the research, along with measures to mitigate the effect of those factors and their cost. Add extra rows as needed.

Factors that are known to contribute to socioeconomic disadvantage can be categorised as the ‘3Ps’; Pockets (income and resource availability), Prospects (expectations and life chances), and Places (housing and local environment). Please see Table 1 for examples.

| **Factors that may prevent full community participation** | **Proposed measures (several options may be needed)** | **Cost of measures** |
| --- | --- | --- |
|  |  |  |
|  |  |  |
|  |  |  |
|  |  |  |
|  |  |  |
|  |  |  |
|  |  |  |
|  |  |  |
|  |  |  |
|  |  |  |
|  |  |  |

**Appendix 1**

**How (and by who) was the INCLUDE Socioeconomic Framework developed?**

The National Institute for Health Research (NIHR) initiated the INCLUDE initiative in 2017. The Medical Research Council (MRC) Hubs for Trials Methodology Research Recruitment and Retention Working Group were concurrently starting efforts to improve representation within trials, particularly of black, Asian and minority ethnic individuals.

The two groups came together in late 2018 to develop a research grant proposal for work on inclusion in trials. Work on the INCLUDE Ethnicity Framework began in earnest in July 2019, and the complete Framework was launched in October 2020. In June 2019 the Medical Research Council (MRC) Hubs for Trials Methodology Research became part of the MRC-NIHR Trials Methodology Research Partnership (TMRP).

The Trial Conduct TMRP working group established the Inclusivity sub-group, which had its first meeting in July 2020, and based on discussions at this meeting, work began on the INCLUDE Socioeconomic Framework in November 2020. In January 2021, a grant application co-led by Frances Sherratt, Heidi Gardener, and Katie Biggs was awarded from The

University of Liverpool Early Career and Returners’ Fund to fund public contributors’ time on the project. A public contributors’ group with six members was assembled in February 2021.

**Phases of developing the NIHR INCLUDE Socio-economic Disadvantage Framework**

| **Phase 1: Developing an outline of what was needed** | | |
| --- | --- | --- |
| **Participants** | | |
| **Name** | **Affiliation** | **Perspective** |
| Heidi Gardner |  |  |
| Fran Sherratt | University of Liverpool | Research Fellow |
| Katie Biggs |  |  |
| Bola Aina |  |  |
| Carolyn Cooper |  |  |
| Clara Barros |  |  |
| John Roberts |  |  |
| Philip Bell |  |  |
|  |  |  |
|  |  |  |

| **Phase 2: Developing an initial draft of the Framework** | | |
| --- | --- | --- |
| **Participants** | | |
| **Name** | **Affiliation** | **Perspective** |
| Heidi Gardner |  |  |
| Fran Sherratt | University of Liverpool | Research Fellow |
| Katie Biggs |  |  |
| Bola Aina |  |  |

| Carolyn Cooper |  |  |
| --- | --- | --- |
| Clara Barros |  |  |
| John Roberts |  |  |
| Philip Bell |  |  |

| **Phase 3: Discussing that draft with a wider stakeholder group** | | |
| --- | --- | --- |
| **Participants** | | |
| **Name** | **Affiliation** | **Perspective** |
| Heidi Gardner |  |  |
| Fran Sherratt | University of Liverpool | Research Fellow |
| Katie Biggs |  |  |
| Bola Aina |  |  |
| Carolyn Cooper |  |  |
| Clara Barros |  |  |
| John Roberts |  |  |
| Philip Bell |  |  |

| **Phase 4: Modifying the draft based on feedback from stakeholders** | | |
| --- | --- | --- |
| **Participants** | | |
| **Name** | **Affiliation** | **Perspective** |
| Heidi Gardner |  |  |
| Fran Sherratt | University of Liverpool | Research Fellow |
| Katie Biggs |  |  |
|  |  |  |
|  |  |  |
|  |  |  |

| **Phase 5: Stakeholder feedback on the modified draft** | | |
| --- | --- | --- |
| **Participants** | | |
| **Name** | **Affiliation** | **Perspective** |
| Heidi Gardner |  |  |
| Fran Sherratt | University of Liverpool | Research Fellow |
| Katie Biggs |  |  |
|  |  |  |
|  |  |  |
|  |  |  |

**Phase 6: Applying the Framework**

**Phase 7: Packaging the Framework, examples, and other materials**
