# Supplementary material for: Making trials more inclusive of people experiencing socioeconomic disadvantage: developing the INCLUDE socioeconomic disadvantage framework
Source: Trials. 2026 Jan 14;27:123. doi: 10.1186/s13063-026-09448-2 (PMC12888448; doi:10.1186/s13063-026-09448-2)
Supplement: Supplementary file 4 — Additional file 4. [file 13063_2026_9448_MOESM4_ESM.docx]

**Additional file 4 Framework Iteration 4. April 2022.**

**Making clinical research more accessible to individuals**

**experiencing socio-economic disadvantage**

**Scope of framework**

This framework has been designed to aid researchers, who are designing clinical trials, to consider barriers to including patients from socio-economically disadvantaged backgrounds in their trial. The framework can also help researchers to develop strategies to attempt to address such barriers, in order to improve the design and conduct of clinical research. Although this framework was developed with UK-based clinical trials in mind, aspects may also be relevant to different types of research and research conducted in populations outside of the UK.

**What is socio-economic disadvantage?**

There is no consensus on a nominal definition of socio-economic status, nor does a widely accepted measure of socio-economic status exist (1). There are many different domains of socio-economic status, but it is typically measured as a combination of income, employment and education (2). In general terms, being socioeconomically disadvantaged includes living in less favourable social and economic circumstances than the majority of others in the same society (3).

Socioeconomic disadvantage is complex, multidimensional and can be dynamic; one’s socio-economic status is not necessarily the same throughout the life course, and events can change socioeconomic status over time. We are aware that the language and terminology used to describe socioeconomic disadvantage can be sensitive. We would welcome any feedback and suggestions that you may have at: [info@trialforge.org](mailto:info@trialforge.org)

**Applying ‘The 3 Ps’**

It is important to note that socioeconomic disadvantage is *not* something that you can see. It is dangerous to make assumptions about people’s backgrounds or experiences. Rather than attempting to identify people, we encourage researchers to identify the potential barriers to clinical trial participation for those from socio-economically disadvantaged backgrounds and develop and implement solutions.

Simplifying clinical trial processes, building trusting relationships, working with patients and public contributors from socio-economically disadvantaged backgrounds, and reducing logistical barriers (e.g. compensation, Internet access, trial information in alternative formats etc.) will contribute to making clinical trials more accessible and increasing representation of people experiencing socio-economic disadvantage in trials.

Indexes of deprivation, which link deprivation domains to postcodes (e.g. English Indices of Deprivation 2019 (4)), have become increasingly sophisticated. However, such indexes remain contentious due to conceptual and practical issues (5). With this in mind we have chosen to develop the present framework, using categories adapted from the UK government’s Child Poverty Strategy 2014-2017 (6) in addition to drawing on associated consultation work (7).

We adapted ‘The 3 Ps’ from this previous work, which entailed three overarching target outcomes to address poverty, and developed the present framework with input from public contributors who described themselves as being from low-income backgrounds or in receipt of government benefits. The 3Ps are a practical way of understanding socioeconomic disadvantage, providing us with a way of questioning our own activities in terms of trial design, so that we can effectively highlight the specific areas that make a trial inaccessible or unacceptable to people experiencing socioeconomic disadvantage. The 3Ps are not intended to be used as a definitive list of the experiences that socioeconomic disadvantage brings, rather, they are a nudge to ensure that trial teams think broadly when completing the worksheets that follow.

Indicator examples (not exhaustive) of socio-economic disadvantage are grouped under one of three headings as below (see also Table 1):

1. Pockets – Indicators closely linked with income and economic resource availability
2. Prospects – Indicators closely linked with wellbeing and life chances
3. Place – Indicators closely linked with housing and local environment

**Table 1.** The 3Ps and associated example indicators of socio-economic disadvantage

| **Pockets** *(Income and economic resource availability)* | **Prospects** *(Expectations and life chances)* | **Place** *(Housing and the local environment)* |
| --- | --- | --- |
| Benefits (e.g. uptake, adequacy, sanctions)  (Un)employment  Childcare  Food poverty / use of food banks  Limited/no access to technological resources  Feeling powerless/ vulnerable due to financial circumstances  Covert situations within relationships (e.g. financial abuse) | Mental health  Household type (e.g. lone parent)  Educational attainment  Literacy  Health literacy  Co-morbidities  Low self-confidence or motivation, which plays into perceived power disparities and mistrust  No/limited access to sources of reliable health/trial information  Acceptance of ‘how it is’ | Housing  Being homeless  Being part of a traveller community  Being in prison  Being an immigrant or refugee  No/limited access to transport systems  No/limited access to community services  Less engagement with healthcare services |

We encourage researchers to consider the 3Ps as a minimum when designing a study. Research has shown that other aspects of identity linked to social inequalities intersect with socioeconomic status, which results in people from minority ethnic groups, people experiencing physical and/or learning disabilities, people living with mental ill-health, people from the LGBTQIA+ community, and women, being at a higher risk of experiencing socioeconomic disadvantage. We encourage trial teams to think carefully about whether and how socioeconomic status intersects with these experiences, and work to implement facilitators and alleviate barriers across groups.

**INCLUDE Key Questions**

This document is designed to encourage trial teams to do everything possible to make their trials relevant to the people that the results are likely to impact (often patients) and those expected to apply them (often healthcare professionals). The four questions below are intended to prompt trial teams to think about who should be involved as participants, and how to facilitate their involvement as much as possible. To identify the barriers and potential solutions to participation, we recommend that trial teams work through the questions below in partnership with patient and public contributors with experience of socioeconomic disadvantage. This is an exercise that should be completed **as a team**, with representation across all of the stakeholders that will ultimately be tasked with designing, delivering, and reporting on the trial.

Note that:

- *‘Intervention*’ means the treatment, initiative or service being evaluated.
- ‘*Comparator*’ means what the intervention is being compared to.
- ‘*Effective*’ means the intervention provides important benefits for people with the disease or condition that is the focus of the trial.

**We recommend that trial teams use the worksheets to help them think through their answers to the four Key Questions…**

**Q1. Who should my trial results apply to?**

Which people could benefit from the intervention if it is found to be effective, or benefit from not having it if it is found to be ineffective and/or harmful? Are people from socio-economically disadvantaged backgrounds routinely omitted from trials in your population or disproportionately affected by the condition or disease?

**Q2. Are people from different socio-economic backgrounds likely to respond to the intervention in different ways?**

Could socio-economic factors influence the way the people identified in question one might respond to, or engage with, the intervention(s) being tested? How might the 3 Ps (i.e. ‘Pockets’ [income and resource availability], ‘Prospects’ [expectations and life chances], and ‘Place’ [housing and the local environment]) affect how people will respond to, or engage with the intervention or the way that their health may change as a result of the intervention?

**Q3. Will my trial intervention and/or comparator make it harder for people from different socio-economic backgrounds to engage with the trial?**

How might the intervention and/or comparator, including how they are delivered, make it harder for some people in the community to take part in the trial? How might the 3 Ps (i.e. ‘Pockets’ [income and resource availability], ‘Prospects’ [expectations and life chances], and ‘Place’ [housing and the local environment]) make it harder for some people to access or accept the intervention or comparator?

**Q4. Will the way I have planned and designed my trial make it harder for people from different socio-economic backgrounds to consider taking part?**

How might elements of trial design, such as patient and public involvement, eligibility criteria, or the recruitment and consent process, make it harder for some people in the community to take part?

**WORKSHEETS**

The four worksheets are intended to be used by trial teams in partnership with patient and public contributors to ensure that people experiencing socio-economic disadvantage are considered at the trial design stage.

The worksheets may cover issues that some trial teams already think about. The intention is that the worksheets will help to highlight issues consistently across trials for all trial teams, as well as raising some questions that may not be routinely considered at present.

Identifying people that may be experiencing socioeconomic disadvantage can be harmful. Instead of trying to make judgements about people’s social and economic circumstances, we encourage trials teams to focus on improving the overall accessibility of their trial in general. This will improve how people access trials across the spectrum of socio-economic status, whilst minimising the risk of arbitrarily and harmfully labelling people.

We encourage trial teams to consider using the 3Ps; Pockets – income and resource availability, Prospects – expectations and life chances, and Places – housing and the local environment, as a starting point when completing the worksheets. See Table 1 for more information on our definition of socioeconomic disadvantage, and where the 3Ps have been used elsewhere.

**Before completing the worksheets,** **you should have answered Question 1 of the INCLUDE Key Questions…**

**Q1. Who should my trial results apply to?**

Which people could benefit from the intervention if it is found to be effective, or benefit from not having it if it is found to be ineffective and/or harmful? Are people from socio-economically disadvantaged backgrounds routinely omitted from trials in your population or disproportionately affected by the condition or disease? It might be useful to consider the incidence of the condition in the general population and speak to healthcare providers about the populations they see in practice.

Once you have identified the people that your trial results could and/or should apply to, we encourage you to seek out opportunities to engage and work alongside people from these groups. If you can include these voices within your team as paid patient and public contributors, you will find the process of completing the worksheets to be a more valuable experience. Drawing on their lived experiences will not only allow you to ensure that you keep existing facilitators in place, it will also highlight barriers throughout the lifecycle of your trial that you should aim to alleviate.

**WORKSHEET A Are people from different socio-economic backgrounds likely to respond to the intervention in different ways? (i.e. Q2 of the INCLUDE Key Questions)**

This worksheet includes questions to guide your thinking about the participation of people experiencing socioeconomic disadvantage when answering Question 2 of the INCLUDE Key Questions.

Could socio-economic factors influence the way the people identified in question one might respond to, or engage with, the intervention(s) being tested? How might the 3 Ps (i.e. ‘Pockets’ [income and resource availability], ‘Prospects’ [expectations and life chances], and ‘Place’ [housing and the local environment]) affect how people will respond to, or engage with their condition or intervention?

|  | **Socio-economic factors that might influence the effectiveness of the intervention for some groups** | | | |
| --- | --- | --- | --- | --- |
|  | **Pockets** | **Prospects** | **Places** | **Other(s)** |
| **Health condition** |  |  |  |  |
| How might the prevalence of the health condition vary between people experiencing socioeconomic disadvantage in the target population? |  |  |  |  |
| How might the severity of the health condition vary between people experiencing socioeconomic disadvantage? |  |  |  |  |
| How might the presentation of the health condition vary between people experiencing socioeconomic disadvantage (this may include symptoms, type or pattern or rate of disease progression)? |  |  |  |  |
| **Culture** |  |  |  |  |
| How might perceptions of the health condition and social stigma around it vary between people experiencing socioeconomic disadvantage and those who are not? |  |  |  |  |
| How might ways of describing the disease vary between people experiencing socioeconomic disadvantage? |  |  |  |  |
| How might the participants’ circumstances influence the acceptability of, and adherence to, the intervention(s) for people experiencing socioeconomic disadvantage? |  |  |  |  |
| How or when might people experiencing socioeconomic disadvantage access healthcare for this disease differently to those who are not? |  |  |  |  |

**WORKSHEET B: Will my trial intervention and/or comparator make it harder for people from different socio-economic backgrounds to engage with the intervention and/or comparator? (i.e. Q3 of the INCLUDE Key Questions)**

How might the intervention and/or comparator, including how they are delivered, make it harder for some people in the community to take part in the trial? How might the 3 Ps (i.e. ‘Pockets’ [income and resource availability], ‘Prospects’ [expectations and life chances], and ‘Place’ [housing and the local environment]) make it harder for some people to access or accept the intervention or comparator?

This worksheet provides some questions to guide your thinking about participation of people experiencing socioeconomic disadvantage when answering Question 3 of the INCLUDE Key Questions.

|  | **Intervention and comparator factors that might affect how participants from some socio-economic groups engage with the intervention and/or comparator*** | | | |
| --- | --- | --- | --- | --- |
|  | **Pockets** | **Prospects** | **Places** | **Other(s)** |
| **Who** |  |  |  |  |
| How might the person delivering the intervention/comparator limit participation of people experiencing socioeconomic disadvantage? |  |  |  |  |
| **What** |  |  |  |  |
| How might the design/delivery of the intervention/comparator limit participation of people experiencing socioeconomic disadvantage? |  |  |  |  |
| How, and in what ways, were people experiencing socioeconomic disadvantage involved in selecting or designing the trial intervention/comparator? |  |  |  |  |
| **When** |  |  |  |  |
| How might when the intervention/comparator is delivered (e.g. during working hours) and/or the frequency/intensity it is delivered (e.g. number of times it is delivered, over what period, time commitment for each session and overall) limit participation of people experiencing socioeconomic disadvantage? |  |  |  |  |
| **Where** |  |  |  |  |
| How might where the intervention/comparator is delivered (e.g. hospital, general practice, local community venues) limit the participation of people experiencing socioeconomic disadvantage? |  |  |  |  |
| **How** |  |  |  |  |
| How might the mode of delivery (e.g. telephone, video-call, face-to-face, in groups) limit the participation of people experiencing socioeconomic disadvantage? |  |  |  |  |

*These factors are taken from TIDieR ([http://www.equator-network.org/reporting-guidelines/tidier/](about:blank)).

**WORKSHEET C: Will the way I have planned and designed my trial make it harder for people from different socio-economic backgrounds to consider taking part? (i.e. Q4 of the INCLUDE Key Questions)**

How might elements of trial design, such as patient and public involvement, eligibility criteria, or the recruitment and consent process, make it harder for some people in the community to take part? Factors that are known to contribute to socioeconomic disadvantage can be categorised as the ‘3Ps’; Pockets (income and resource availability), Prospects (expectations and life chances), and Places (housing and local environment). Please see Table 1 for examples.

This four-part worksheet provides some questions to guide your thinking about participation of people experiencing socioeconomic disadvantage when answering Question 4 of the INCLUDE Key Questions. Worksheet C is divided into four sub-worksheets:

C.1: Trial eligibility and information

C.2: Data collection

C.3: Data analysis

C.4: Reporting and dissemination

**WORKSHEET C.1: Trial eligibility and accessibility factors that might affect how some groups engage with the trial**

|  | **Pockets** | **Prospects** | **Places** | **Other(s)** |
| --- | --- | --- | --- | --- |
| **Eligibility** |  |  |  |  |
| How might eligibility criteria exclude people experiencing socioeconomic disadvantage for reasons other than their clinical eligibility for the trial (e.g. availability of medical history, language requirements, location, gender, age, discussing pregnancy, internet/mobile telephone access)? |  |  |  |  |
| **Trial information** |  |  |  |  |
| How might the way(s) (and by whom) potential participants are made aware of the trial (e.g. posters in a clinic, letterheaded paper in brown envelope that has negative associations for participant [e.g. debt agency], documents written in plain English, who approaches the participant about the trial) limit the participation of people experiencing socioeconomic disadvantage? |  |  |  |  |
| How might the mode and format of the information that tells potential participants about the trial (e.g. participant information leaflet, online, video) limit the participation of people experiencing socioeconomic disadvantage? |  |  |  |  |
| How might potential participants’ cultural practices, beliefs and traditions change the way that people experiencing socioeconomic disadvantage perceive the information they are given? |  |  |  |  |

**WORKSHEET C.2: Trial data collection factors that might affect how some groups engage with the trial**

|  | **Pockets** | **Prospects** | **Places** | **Other(s)** |
| --- | --- | --- | --- | --- |
| **Who** |  |  |  |  |
| How might the people who collect data limit the participation of people experiencing socioeconomic disadvantage (e.g. role, power dynamics, relationship)? |  |  |  |  |
| **What** |  |  |  |  |
| How, and in what way, were people experiencing socioeconomic disadvantage involved in selecting the trial outcomes? |  |  |  |  |
| How might the trial outcomes themselves, or other data being collected (e.g. participant’s background information) limit the participation of people experiencing socioeconomic disadvantage? |  |  |  |  |
| **When** |  |  |  |  |
| How might when the data is collected (e.g. appointments conflicting with work or childcare responsibilities) and/or the frequency/duration it is collected (e.g. number of follow up appointments, length of questionnaires) limit participation of people experiencing socioeconomic disadvantage? |  |  |  |  |
| **Where** |  |  |  |  |
| How might the location where trial data are collected limit participation of people experiencing socioeconomic disadvantage (e.g. no/limited disposable income for additional public transport, don’t drive, poor transport links, feeling uncomfortable in setting)? |  |  |  |  |
| **How?** |  |  |  |  |
| How might data collection methods (e.g. questionnaire format and length, additional clinical tests, mode of collection [e.g. option for various modes such as app, via telephone, paper questionnaire], type of remuneration and impact on government benefits i.e. voucher/cash) limit the participation of people experiencing socioeconomic disadvantage? |  |  |  |  |

**WORKSHEET C.3: Factors that might affect the analysis of trial results**

|  | **Pockets** | **Prospects** | **Places** | **Other(s)** |
| --- | --- | --- | --- | --- |
| **Representativeness** |  |  |  |  |
| How well might trial participants represent your target population, when considering your approach to recruitment? |  |  |  |  |
| **Retention** |  |  |  |  |
| How might the accuracy and completeness of trial data collected differ between socio-economic groups in the target population (i.e. participants from the most socio-economically disadvantaged backgrounds compared to participants from the least)? |  |  |  |  |
| **Intervention benefits** |  |  |  |  |
| How might the benefits of the trial intervention(s) differ between socio-economic groups in the target population? |  |  |  |  |
| **Intervention harms** |  |  |  |  |
| How might the possible harms of the trial intervention(s) differ between socioeconomic groups in the target population? |  |  |  |  |
| **Subgroup analyses** |  |  |  |  |
| How should variation between socioeconomic groups in the target population be explored– should there be planned subgroup analyses? |  |  |  |  |
| **Interim analyses** |  |  |  |  |
| How should any interim analysis handle variation between socioeconomic groups in the target population? |  |  |  |  |
| **Stopping triggers** |  |  |  |  |
| How should any rules to stop the trial early on intervention safety or intervention benefit grounds handle variation between socioeconomic groups in the target population? |  |  |  |  |

**WORKSHEET C.4: Factors that might affect the reporting and dissemination of trial results**

|  | **Pockets** | **Prospects** | **Places** | **Other(s)** |
| --- | --- | --- | --- | --- |
| **Planning reporting** |  |  |  |  |
| How, and in what way, are people experiencing socioeconomic disadvantage involved in planning the reporting and dissemination of the trial results? |  |  |  |  |
| **Dissemination engagement** |  |  |  |  |
| How, and in what way, might your reporting and dissemination of trial results limit engagement of people experiencing socioeconomic disadvantage? Think about both the mode (i.e., how) and content (i.e., what) by which you are disseminating your results. |  |  |  |  |

**WORKSHEET D: What measures can I put in place to address the identified factors that might prevent optimal participations of the people from socio-economically disadvantaged backgrounds?**

Use this worksheet to summarise the key factors you have identified that could prevent people experiencing socio-economic disadvantage from fully participating in the trial, along with measures to mitigate the effect of those factors and their cost. Add extra rows as needed.

| **Factors that may prevent full community participation** | **Proposed measures (several options may be needed)** | **Cost of measures** |
| --- | --- | --- |
|  |  |  |
|  |  |  |
|  |  |  |
|  |  |  |
|  |  |  |
|  |  |  |
|  |  |  |
|  |  |  |
|  |  |  |
|  |  |  |
|  |  |  |

**Appendix 1**

**Framework background and next steps**

The National Institute for Health Research (NIHR) initiated the INCLUDE initiative in 2017. The Medical Research Council (MRC) Hubs for Trials Methodology Research Recruitment and Retention Working Group were concurrently starting efforts to improve representation within trials, particularly minority ethnic individuals.

The two groups came together in late 2018 to develop a research grant proposal for work on inclusion in trials. Work on the INCLUDE Ethnicity Framework began in earnest in July 2019, and the complete Framework was launched in October 2020.

In June 2019 the Medical Research Council (MRC) Hubs for Trials Methodology Research became part of the MRC-NIHR Trials Methodology Research Partnership (TMRP). The Trial Conduct TMRP working group established the Inclusivity sub-group, which had its first meeting in July 2020, and based on discussions at this meeting, work began on the INCLUDE Socioeconomic Framework in November 2020. In January 2021, a grant application co-led by Frances Sherratt, Heidi Gardner, and Katie Biggs was awarded from The University of Liverpool Early Career and Returners’ Fund to fund public contributors’ time on the project. A public contributors’ group with six members was assembled in February 2021.

In the future, we would like to evaluate this framework to consider how useful and effective it is at supporting researchers to identify and work to address barriers to making clinical trials inclusive for patients from socio-economically disadvantaged backgrounds. We are aware of work currently ongoing to evaluate the INCLUDE Ethnicity Framework, which we expect to build on with our evaluation. Findings from the evaluation will be used to inform the development of current and future frameworks designed to improve inclusivity in research. Furthermore, members of MRC-NIHR Trials Methodology Research Partnership Inclusivity working group are currently in the process of appointing a PhD student that will work on a project exploring the possibility of combining several recently developed frameworks for different under-served groups; their primary focus will be on combining Frameworks covering ethnicity, socio-economic disadvantage, and people without capacity to give consent, this may grow to include additional under-served groups as new frameworks are developed over the coming years. Doing so will help to address the intersectionality of many under-served characteristics, as well as ensuring trial teams are able to efficiently use their time to design inclusive trials in advance of submitting funding applications.

**Phases of developing the NIHR INCLUDE Socio-economic Disadvantage Framework**

| **Phase 1: Developing an outline of what was needed** | | |
| --- | --- | --- |
| **Participants** | | |
| **Name** | **Affiliation** | **Perspective** |
| Heidi Gardner | University of Aberdeen | Research Fellow, first academic in the family, working/middle-class background. |
| Fran Sherratt | University of Liverpool | Research Fellow |
| Katie Biggs | University of Sheffield | Research Fellow |
|  |  |  |
|  |  |  |
|  |  |  |
|  |  |  |
|  |  |  |
|  |  |  |
|  |  |  |

| **Phase 2: Developing an initial draft of the Framework** | | |
| --- | --- | --- |
| **Participants** | | |
| **Name** | **Affiliation** | **Perspective** |
| Heidi Gardner | University of Aberdeen | Research Fellow |
| Fran Sherratt | University of Liverpool | Research Fellow |
| Katie Biggs | University of Sheffield | Research Fellow |
|  |  |  |
|  |  |  |
|  |  |  |
|  |  |  |
|  |  |  |

| **Phase 3: Discussing that draft with a wider stakeholder group** | | |
| --- | --- | --- |
| **Participants** | | |
| **Name** | **Affiliation** | **Perspective** |
| Heidi Gardner | University of Aberdeen | Research Fellow |
| Fran Sherratt | University of Liverpool | Research Fellow |
| Katie Biggs | University of Sheffield | Research Fellow |
|  |  |  |
|  |  |  |
|  |  |  |
|  |  |  |
|  |  |  |

| **Phase 4: Modifying the draft based on feedback from stakeholders** | | |
| --- | --- | --- |
| **Participants** | | |
| **Name** | **Affiliation** | **Perspective** |
| Heidi Gardner | University of Aberdeen | Research Fellow |
| Fran Sherratt | University of Liverpool | Research Fellow |
| Katie Biggs | University of Sheffield | Research Fellow |
|  |  |  |
|  |  |  |
|  |  |  |

| **Phase 5: Stakeholder feedback on the modified draft** | | |
| --- | --- | --- |
| **Participants** | | |
| **Name** | **Affiliation** | **Perspective** |
| Heidi Gardner | University of Aberdeen | Research Fellow |
| Fran Sherratt | University of Liverpool | Research Fellow |
| Katie Biggs | University of Sheffield | Research Fellow |
|  |  |  |
|  |  |  |
|  |  |  |

**Phase 6: Applying the Framework**

**Phase 7: Packaging the Framework, examples, and other materials**

**References**

1. Oakes JM, Rossi PH. The measurement of SES in health research: current practice and steps toward a new approach. Soc Sci Med. 2003;56(4):769-84.

2. Raina SK. Use of socioeconomic status scales in medicine and public health. J Family Med Prim Care. 2015;4(1):156-.

3. Scottish Government. Consultation on the socio-economic duty: Analysis of responses. 2017.

4. Ministry of Housing CLG. English Indices of Deprivation 2019 2019 [Available from: <https://www.gov.uk/government/statistics/english-indices-of-deprivation-2019>.

5. Clelland D, Hill C. Deprivation, policy and rurality: The limitations and applications of area-based deprivation indices in Scotland. Local Economy. 2019;34(1):33-50.

6. Government S. Child Poverty Strategy for Scotland - Our Approach - 2014-2017 2014 [Available from: <https://www.gov.scot/publications/child-poverty-strategy-scotland-approach-2014-2017/>.

7. Scottish Government. Consultation on a Child Poverty Bill for Scotland: Analysis of Responses. 2016.
