# Supplementary material for: Making trials more inclusive of people experiencing socioeconomic disadvantage: developing the INCLUDE socioeconomic disadvantage framework
Source: Trials. 2026 Jan 14;27:123. doi: 10.1186/s13063-026-09448-2 (PMC12888448; doi:10.1186/s13063-026-09448-2)
Supplement: Supplementary file 5 — Additional file 5. [file 13063_2026_9448_MOESM5_ESM.docx]

**Additional file 5 Public contributor considerations.**

**Public contributor considerations to support researchers in making trials more accessible to people experiencing socioeconomic disadvantage**

Public contributors described how socioeconomic disadvantage is an extremely complex construct to measure. They suggested that there could be unique challenges in making clinical research more inclusive to patients from socioeconomically disadvantaged backgrounds, compared with some other underserved groups, such as patients from different ethnic minority groups. They posited that socioeconomic status is a multidimensional and dynamic concept, with many describing their own experiences of how life events had altered their socioeconomic identities across the life course.

Contributors agreed that it would be inappropriate and dangerous for health professionals to judge and categorise individual patients by socioeconomic status to improve the inclusivity of research. One contributor illustrated this point by saying that health professionals might ask, “I know this patient has diabetes but how do I know if they are from a socioeconomically disadvantaged background?” They proposed that rather than trying to categorise patients, trialists should be focusing their efforts on providing the same to everyone but make resources and processes simpler and show patients that they care. They spoke about the stigma associated with socioeconomic disadvantage, which can make it a taboo subject. It was suggested that this might hamper efforts to make research more inclusive. Public contributors suggested that it would be better to focus on making people feel more valued for their contribution and reframing socioeconomic status, which would encompass viewing socioeconomic disadvantage as an attribute because patients from such groups can offer a valuable perspective and provide representation for a significant proportion of the population.

The next few sections will focus on some of the key barriers to including patients from socioeconomically disadvantaged backgrounds in health research and suggested strategies to address them. Similar to the INCLUDE Socioeconomic Disadvantage Framework, we have grouped barriers to inclusion and associated strategies under one of three headings: (1) Pockets – Barriers and strategies linked with income and economic resource availability; (2) Prospects – Barriers and strategies linked with wellbeing and life chances, and; (3) Place – Barriers and strategies linked with housing and local environment.

We acknowledge that the below is not an exhaustive list of all opportunities to improve inclusivity of research for patients from socioecomically disadvantaged groups and that also, many of the factors interlink. Researchers should consider a holistic approach to addressing any identified barriers to making research inclusive, but perhaps most importantly, include patients and the public from socioeconomically disadvantaged backgrounds as co-partners in health research from conception to dissemination as a crucial step to make research more accessible and inclusive.

***Pockets: Barriers to inclusion and strategies linked with income and economic resource availability***

One of the key barriers to inclusion that public contributors identified was the financial expense that participants might incur when participating in a clinical study. The main expense discussed was travel. Contributors explained that any additional travel expense that is incurred as a result of participating in research should be reimbursed. Ideally, travel arrangements should be organised and paid for in advance to avoid participants having to use their own money. One contributor suggested that although public transport might be available and free, this might not be ideal for all patients, such as those who are vulnerable to infection.

Another key barrier was childcare. Contributors described how parents or carers, especially single parents, might find it difficult to attend research appointments without childcare support. Contributors suggested that financial support for childcare would enable some patients to participate in research who might not otherwise. Consideration should also be given to patients with children of school age to ensure that research appointments can be made inside of school hours if needed.

Patients from socioeconomically disadvantaged backgrounds might also have limited access to technological resources that would support participation. Contributors described how digital, remote trials could promote inclusion overall, but that patients who might not have access to computers, mobile devices, or the Internet, could be excluded. Some suggestions to address this included offering alternative formats to participate and providing financial support for resources, such as mobile data usage.

Contributors suggested that patients from socioeconomic disadvantaged groups might find it more difficult to get time off work for research appointments. They suggested that trials teams should provide participants with a letter for their employer to support a request for time off work to participate. For patients in receipt of government benefits, trial teams need to communicate any payments that are offered as part of the research, be clear about these, and highlight any impact that it might have on their benefits. Contributors suggested that offering the choice of vouchers or cash might help to avoid any impact on participants’ benefits.

***Prospects: Barriers and strategies linked with wellbeing and life chances***

Contributors highlighted how socioeconomic disadvantage often overlaps with several other underserved groups, such as physical or learning disabilities, low health literacy, and language barriers. They described the consent process as overwhelming and proposed that it could be improved by ensuring consent materials are concise, offered in different formats (e.g. videos, information sheets etc.), modes (e.g. e-consent, face-to-face etc.) and languages, and key pieces of information highlighted. Interpreters should be accessible to those who might need them. Patient-facing materials should avoid acronyms and jargon, use more descriptive terms and plain English, and be visually appealing. One contributor also suggested being sensitive to how information is sent to participants and described how use of letterheaded paper in a brown envelope could evoke negative associations (e.g. debt agency).

Contributors described how patients from socioeconomically disadvantaged groups might be more vulnerable to low self esteem and mental health conditions, such as anxiety. Contributors spoke about how some patients worry that doctors might make assumptions about their intelligence, level of education, or make judgements about them if they are receiving government benefits. These are some of the factors that were suggested to contribute to patients feeling out-of-place, powerless, and excluded in patient-doctor research consultations. Contributors described how many patients might be wary or untrusting of research. Although contributors felt that social media could be a useful platform to improve research awareness as it might be a main source of information for many patients, they also suggested that some patients might be vulnerable conspiracy theories and inaccurate information about research.

Contributors proposed that health professionals and trials teams could be doing more to empower and motivate patients, develop trust, and balance power in the patient-doctor relationship. Health professionals clearly communicating how participating benefits to both the patient and the research team and the trial team providing the participant with regular updates on how their contribution has been valuable will contribute to achieving this. Patients may also feel more empowered by having fewer health professionals in consultations, being accompanied by a friend or family member, and depending on the context, involving community champions or advocates.

***Place: Barriers and strategies linked with housing and local environment***

In addition to the barriers already identified around travel and childcare expenses, patients from socioeconomically disadvantaged backgrounds might also have no or limited access to travel or childcare. This further supports why, where possible, participants should be offered a choice of consultation environment (e.g. home, clinic etc.), mode (e.g. face-to-face, telephone, video call etc.), and times. Contributors suggested that research teams should widen approaches to advertising research opportunities, and consider platforms, such as social media and local advertising in community venues. Some contributors reiterated that making assumptions about where a person lives should be avoided and that asking for someone’s address could exclude them. They emphasised the importance of speaking to public contributors from socioeconomically diverse backgrounds to check finer research study details, as opposed to only having involvement at the design stage.
