# Supplementary material for: Making trials more inclusive of people experiencing socioeconomic disadvantage: developing the INCLUDE socioeconomic disadvantage framework
Source: Trials. 2026 Jan 14;27:123. doi: 10.1186/s13063-026-09448-2 (PMC12888448; doi:10.1186/s13063-026-09448-2)
Supplement: Supplementary file 6 — Additional file 6. [file 13063_2026_9448_MOESM6_ESM.docx]

**
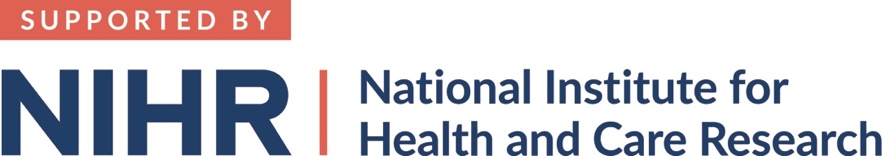

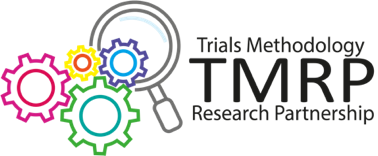

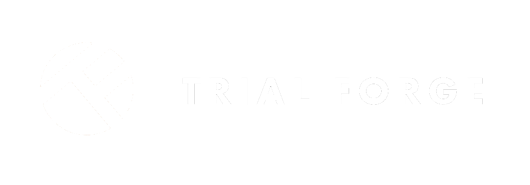

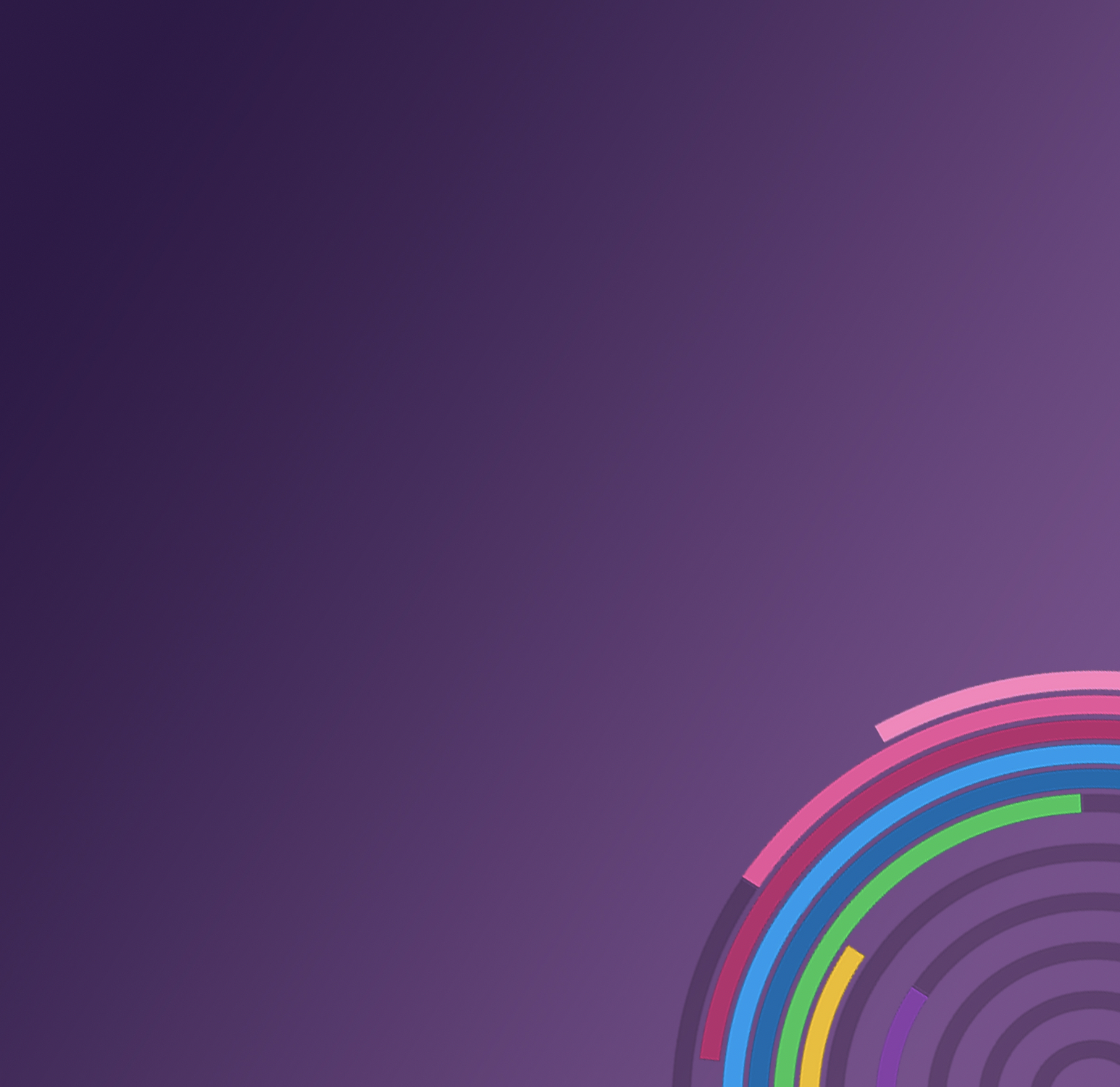
**

Additional file 6 Finalised Framework

THE NIHR INCLUDE SOCIOECONOMIC DISADVANTAGE FRAMEWORK

NIHR INCLUDE Socioeconomic Disadvantage Framework:

Making clinical research more accessible to individuals experiencing socioeconomic disadvantage

Scope of framework

This framework has been designed to aid researchers, who are designing clinical trials, to consider barriers to including patients from socioeconomically disadvantaged backgrounds in their trial. The framework can also help researchers to develop strategies to attempt to address such barriers in order to improve the design and conduct of clinical research. Although this framework was developed with UK-based clinical trials in mind, aspects may also be relevant to different types of research and research conducted in populations outside of the UK. Whilst this framework focuses on socioeconomic disadvantage, the list of underserved groups in clinical research is extensive (1) and researchers need to be aware of this when identifying barriers to research and developing to strategies to address barriers.

What is socioeconomic disadvantage?

There is no consensus on a definition of socioeconomic status, nor does a widely accepted measure of socioeconomic status exist (2). There are many different domains of socioeconomic status, but it is typically measured as a combination of income, employment and education (3). In general terms, being socioeconomically disadvantaged includes living in less favourable social and economic circumstances than the majority of others in the same society (4).

Socioeconomic disadvantage is complex, multidimensional and can be dynamic; one’s socioeconomic status is not necessarily the same throughout the life course, and events can change socioeconomic status over time. We are aware that the language and terminology used to describe socioeconomic disadvantage can be sensitive. We would welcome any feedback and suggestions for improvement that you may have at: [info@trialforge.org](mailto:info@trialforge.org)

Applying ‘The 3 Ps’

It is important to note that socioeconomic disadvantage is *not* something that you can always see, and we therefore can’t make assumptions about people’s backgrounds or experiences. Rather than attempting to identify people, we encourage researchers to identify the potential barriers to clinical trial participation for those from socioeconomically disadvantaged backgrounds and develop and implement solutions.

Simplifying clinical trial processes, building trusting relationships, working with patients and public contributors from socioeconomically disadvantaged backgrounds, and reducing logistical barriers (e.g. through compensation, Internet access, trial information in alternative formats etc.) will contribute to making clinical trials more accessible and is likely to increase representation of people experiencing socioeconomic disadvantage in trials.

Indexes of socioeconomic disadvantage, which link disadvantage domains to postcodes (e.g. English Indices of Deprivation 2019 (5)), have become increasingly sophisticated. However, such indexes remain contentious due to conceptual and practical issues, with particular concerns about their applicability to rural areas (6). With this in mind we have chosen to develop the NIHR INCLUDE Socioeconomic Disadvantage Framework using categories adapted from the UK Government’s Child Poverty Strategy 2014-2017 (7), in addition to drawing on associated consultation work linked to the strategy (8).

‘The 3 Ps’ are a practical way of understanding socioeconomic disadvantage, providing us with a way of questioning our own activities in terms of trial design, so that we can effectively highlight the specific areas that make a trial inaccessible or unacceptable to people experiencing socioeconomic disadvantage. The 3Ps are not intended to be used as a definitive list of the experiences that socioeconomic disadvantage brings, rather, they are a nudge to ensure that trial teams think broadly when completing the worksheets that follow. We adapted ‘The 3 Ps’ from the Child Poverty Strategy work, which entailed three overarching target outcomes to address poverty, and developed the present framework with input from public contributors who described themselves as being from low-income backgrounds or in receipt of government benefits.

Indicator examples (not exhaustive) of socioeconomic disadvantage are grouped under one of three headings as below (see also Table 1):

1. Pockets – Indicators closely linked with income and economic resource availability
2. Prospects – Indicators closely linked with wellbeing and life chances
3. Place – Indicators closely linked with housing and local environment

**Table 1.** The 3Ps and associated example indicators of socioeconomic disadvantage

| **Pockets** *(Income and economic resource availability)* | **Prospects** *(Expectations and life chances)* | **Place** *(Housing and the local environment)* |
| --- | --- | --- |
| Benefits (e.g. uptake, adequacy, sanctions)  Unemployment  Low income  Childcare  Food poverty / use of food banks  Limited/no access to technological resources  Feeling powerless/ vulnerable due to financial circumstances  Covert situations within relationships (e.g. financial abuse) | Mental health  Household type (e.g. lone parent)  Educational attainment  Literacy  Health literacy  Co-morbidities  Low self-confidence or motivation, which plays into perceived power disparities and mistrust  No/limited access to sources of reliable health/trial information  Acceptance of ‘how it is’  Intersectionality with other underserved and/or vulnerable groups | Housing  Being homeless  Being part of a traveller community  Being in prison  Being an immigrant or refugee  No/limited access to transport systems  No/limited access to community services  Local labour market  Local services (e.g. access to childcare)  Healthcare access and engagement |

We encourage researchers to consider the 3Ps as a minimum when designing a study. Research has shown that other aspects of identity linked to social inequalities intersect with socioeconomic status, which results in people from minority ethnic groups, people experiencing physical and/or learning disabilities, people living with mental ill-health, people from the LGBTQIA+ community, and women, being at a higher risk of experiencing socioeconomic disadvantage. Furthermore, where someone lives can influence inequalities and how they are experienced. We encourage trial teams to think carefully about whether and how socioeconomic status intersects with these experiences, and work to implement facilitators and alleviate barriers across groups.

INCLUDE Key Questions

This NIHR INCLUDE Socioeconomic Disadvantage Framework is designed to encourage trial teams to do everything possible to make their trials relevant to the people that the results are likely to impact (often patients) and those expected to apply them (often healthcare professionals). The four questions below are intended to prompt trial teams to think about who should be involved as participants, and how to facilitate their involvement as much as possible. To identify the barriers and potential solutions to participation, we recommend that trial teams work through the questions below in partnership with patient and public contributors with experience of socioeconomic disadvantage. This is an exercise that should be completed **as a team**, with representation across all of the stakeholders that will ultimately be tasked with designing, delivering, and reporting on the trial.

Note that:

- *‘Intervention*’ means the treatment, initiative or service being evaluated.
- ‘*Comparator*’ means what the intervention is being compared to.
- ‘*Effective*’ means the intervention provides important benefits for people with the disease or condition that is the focus of the trial.

We recommend that trial teams think through the answers to the four Key Questions (below), with the aid of the provided worksheets:

| **Q1. Who should my trial results apply to?** |  |
| --- | --- |
| Which people could benefit from the intervention if it is found to be effective, or benefit from not having it if it is found to be ineffective and/or harmful? Are people from socioeconomically disadvantaged backgrounds routinely omitted from trials in your population or disproportionately affected by the condition or disease? | |
| **Q2. Are people from different socioeconomic backgrounds likely to respond to the intervention in different ways?** | |
| Could socioeconomic factors influence the way the people identified in Question 1 might respond to, or engage with, the intervention(s) being tested? How might the 3 Ps (see Table 1) affect how people will respond to, or engage with the intervention or the way that their health may change as a result of the intervention? | |
| **Q3. Will my trial intervention and/or comparator make it harder for people from different socioeconomic backgrounds to take part in the trial?** | |
| How might the intervention and/or comparator, including how they are delivered, make it harder for some people in the community to take part in the trial? How might the 3 Ps (see Table 1) make it harder for some people to access or accept the intervention or comparator? | |
| **Q4. Will the way I have planned and designed my trial make it harder for people from different socioeconomic backgrounds to take part?** | |
| How might elements of trial design, such as patient and public involvement (i.e. whether it is representative of the patient population), eligibility criteria, or the recruitment and consent process, make it harder for some people in the community to take part? | |

WORKSHEETS

The four worksheets are intended to be used by trial teams, including relevant stakeholders such as patient and public contributors, to ensure that people experiencing socioeconomic disadvantage are considered at the trial design stage.

The worksheets may cover issues that some trial teams already think about. The intention is that the worksheets will help to highlight issues consistently across trials for all trial teams, as well as raising some questions that may not be routinely considered at present.

Identifying people that may be experiencing socioeconomic disadvantage can be harmful, as the presence of implicit biases among health professionals can influence the quality of clinical care patients receive (9). Instead of trying to make judgements about people’s social and economic circumstances, we encourage trials teams to focus on improving the overall accessibility of their trial in general. This will improve how people access trials across the spectrum of socioeconomic status, whilst minimising the risk of arbitrarily and harmfully labelling people.

**We encourage trial teams to consider using the 3Ps; Pockets – income and resource availability, Prospects – expectations and life chances, and Places – housing and the local environment, as a starting point when completing the worksheets. See Table 1 for more information on our definition of socioeconomic disadvantage, and where the 3Ps have been used elsewhere.**

Before completing the worksheets, you should have answered Question 1 of the INCLUDE Key Questions…

| **Q1. Who should my trial results apply to?** |  |
| --- | --- |
| Which people could benefit from the intervention if it is found to be effective, or benefit from not having it if it is found to be ineffective and/or harmful? Are people from socioeconomically disadvantaged backgrounds routinely omitted from trials in your population or disproportionately affected by the condition or disease? It might be useful to consider the incidence of the condition in the general population and speak to healthcare providers about the populations they see in practice.  Once you have identified the people that your trial results could and/or should apply to, we encourage you to seek out opportunities to engage and work alongside people from these groups. If you can include these voices within your team as paid patient and public contributors, you will find the process of completing the worksheets to be a more valuable experience. Drawing on their lived experiences will not only allow you to ensure that you keep existing facilitators in place, it will also highlight barriers throughout the lifecycle of your trial that you should aim to alleviate. | |

**Worksheet A: Are people from different socioeconomic backgrounds likely to respond to the intervention in different ways? (i.e. Q2 of the INCLUDE Key Questions)**

This worksheet includes questions to guide your thinking about the participation of people experiencing socioeconomic disadvantage when answering Question 2 of the INCLUDE Key Questions.

Could socioeconomic factors influence the way the people identified in question one might respond to, or engage with, the intervention(s) being tested? How might the 3 Ps (i.e. ‘Pockets’ [income and resource availability], ‘Prospects’ [expectations and life chances], and ‘Place’ [housing and the local environment]) affect how people will respond to, or engage with their condition or intervention?

|  | **Socioeconomic factors that might influence the effectiveness of the intervention for some groups** | | | |  |
| --- | --- | --- | --- | --- | --- |
|  | **Pockets** | **Prospects** | **Places** | **Other(s)** | |
| **Health condition** |  |  |  |  | |
| How might the prevalence of the health condition vary between people experiencing socioeconomic disadvantage and those who are not? |  |  |  |  | |
| How might the severity of the health condition vary between people experiencing socioeconomic disadvantage and those who are not? |  |  |  |  | |
| How might the presentation of the health condition vary between people experiencing socioeconomic disadvantage and those who are not? (this may include symptoms, type or pattern or rate of disease progression)? |  |  |  |  | |
| **Perspectives** |  |  |  |  | |
| How might perceptions of the health condition and social stigma around it vary between people experiencing socioeconomic disadvantage and those who are not? |  |  |  |  | |
| How might ways of describing the disease vary between people experiencing socioeconomic disadvantage and those who are not? |  |  |  |  | |
| How might participants’ acceptability of, and adherence to, the intervention(s) vary between people experiencing socioeconomic disadvantage and those who are not? |  |  |  |  | |
| How or when might healthcare access vary between people experiencing socioeconomic disadvantage and those who are not? |  |  |  |  | |

**Worksheet B: Will my trial intervention and/or comparator make it harder for people from different socioeconomic backgrounds to take part in the trial? (i.e. Q3 of the INCLUDE Key Questions)**

How might the intervention and/or comparator, including how they are delivered, make it harder for some people in the community to take part in the trial? How might the 3 Ps (i.e. ‘Pockets’ [income and resource availability], ‘Prospects’ [expectations and life chances], and ‘Place’ [housing and the local environment]) make it harder for some people to access or accept the intervention or comparator?

This worksheet provides some questions to guide your thinking about participation of people experiencing socioeconomic disadvantage when answering Question 3 of the INCLUDE Key Questions.

|  | **Intervention and comparator factors that might affect how participants from some socioeconomic groups engage with the intervention and/or comparator*** | | | |  |
| --- | --- | --- | --- | --- | --- |
|  | **Pockets** | **Prospects** | **Places** | **Other(s)** | |
| **Who** |  |  |  |  | |
| How might the person delivering the intervention/comparator limit participation of people experiencing socioeconomic disadvantage compared to those who are not? |  |  |  |  | |
| **What** |  |  |  |  | |
| How might the design/delivery of the intervention/comparator limit participation of people experiencing socioeconomic disadvantage compared to those who are not? |  |  |  |  | |
| How, and in what ways, were people experiencing socioeconomic disadvantage involved in selecting or designing the trial intervention/comparator compared with those who are not? |  |  |  |  | |
| **When** |  |  |  |  | |
| How might when the intervention/comparator is delivered (e.g. during working hours) and/or the frequency/intensity it is delivered (e.g. number of times it is delivered, over what period, time commitment for each session and overall) limit participation of people experiencing socioeconomic disadvantage compared with those who are not? |  |  |  |  | |
| **Where** |  |  |  |  | |
| How might where the intervention/comparator is delivered (e.g. hospital, general practice, local community venues) limit the participation of people experiencing socioeconomic disadvantage compared with those who are not? |  |  |  |  | |
| **How** |  |  |  |  | |
| How might the mode of delivery (e.g. telephone, video-call, face-to-face, in groups) limit the participation of people experiencing socioeconomic disadvantage compared with those who are not? |  |  |  |  | |

*These factors are taken from TIDieR ([http://www.equator-network.org/reporting-guidelines/tidier/](about:blank)).

**Worksheet C: Will the way I have planned and designed my trial make it harder for people from different socioeconomic backgrounds to take part in the trial? (i.e. Q4 of the INCLUDE Key Questions)**

How might elements of trial design, such as patient and public involvement, eligibility criteria, or the recruitment and consent process, make it harder for some people in the community to take part? Factors that are known to contribute to socioeconomic disadvantage can be categorised as the ‘3Ps’; Pockets (income and resource availability), Prospects (expectations and life chances), and Places (housing and local environment). Please see Table 1 for examples.

This four-part worksheet provides some questions to guide your thinking about participation of people experiencing socioeconomic disadvantage when answering Question 4 of the INCLUDE Key Questions. Worksheet C is divided into four sub-worksheets:

C.1: Trial eligibility and information

C.2: Data collection

C.3: Data analysis

C.4: Reporting and dissemination

**Worksheet C.1: Trial eligibility and accessibility factors that might affect how some groups engage with the trial**

|  | **Pockets** | **Prospects** | **Places** | **Other(s)** |
| --- | --- | --- | --- | --- |
| **Eligibility** |  |  |  |  |
| How might eligibility criteria exclude people experiencing socioeconomic disadvantage for reasons other than their clinical eligibility for the trial (e.g. availability of medical history, language requirements, location, gender, age, discussing pregnancy, internet/mobile telephone access) compared with those who are not? |  |  |  |  |
| **Trial information** |  |  |  |  |
| How might the way(s) (and by whom) potential participants are made aware of the trial (e.g. posters in a clinic, letterheaded paper in brown envelope that has negative associations for participant [e.g. debt agency], documents written in plain English, who approaches the participant about the trial) limit the participation of people experiencing socioeconomic disadvantage compared to those who are not? |  |  |  |  |
| How might the mode and format of the information that tells potential participants about the trial (e.g. participant information leaflet, online, video) limit the participation of people experiencing socioeconomic disadvantage compared with those who are not? |  |  |  |  |
| How might potential participants’ cultural practices, beliefs and traditions change the way that people experiencing socioeconomic disadvantage perceive the information they are given compared with those who are not? |  |  |  |  |

**Worksheet C.2: Trial data collection factors that might affect how some groups engage with the trial**

|  | **Pockets** | **Prospects** | **Places** | **Other(s)** |
| --- | --- | --- | --- | --- |
| **Who** |  |  |  |  |
| How might the people who collect data limit the participation of people experiencing socioeconomic disadvantage (e.g. role, power dynamics, relationship) compared with those who are not? |  |  |  |  |
| **What** |  |  |  |  |
| How, and in what way, were people experiencing socioeconomic disadvantage involved in selecting the trial outcomes compared with those who are not? |  |  |  |  |
| How might the trial outcomes themselves, or other data being collected (e.g. participant’s background information) limit the participation of people experiencing socioeconomic disadvantage compared with those who are not? |  |  |  |  |
| **When** |  |  |  |  |
| How might when the data is collected (e.g. appointments conflicting with work or childcare responsibilities) and/or the frequency/duration it is collected (e.g. number of follow up appointments, length of questionnaires) limit participation of people experiencing socioeconomic disadvantage compared with those who are not? |  |  |  |  |
| **Where** |  |  |  |  |
| How might the location where trial data are collected limit participation of people experiencing socioeconomic disadvantage compared with those who are not? (e.g. no/limited disposable income for additional public transport, don’t drive, poor transport links, feeling uncomfortable in setting) |  |  |  |  |
| **How?** |  |  |  |  |
| How might data collection methods limit the participation of people experiencing socioeconomic disadvantage compared with those who are not? (e.g. questionnaire format and length, additional clinical tests, mode of collection [e.g. option for various modes such as app, via telephone, paper questionnaire], type of remuneration and impact on government benefits i.e. voucher/cash) |  |  |  |  |

**Worksheet C.3: Factors that might affect the analysis of trial results**

|  | **Pockets** | **Prospects** | **Places** | **Other(s)** |
| --- | --- | --- | --- | --- |
| **Representativeness** |  |  |  |  |
| When considering your approach to recruitment, how well represented might participants experiencing socioeconomic disadvantage be compared with the target population? |  |  |  |  |
| **Retention** |  |  |  |  |
| How might the accuracy and completeness of trial data collected differ between participants experiencing socioeconomic disadvantage compared with those who are not? |  |  |  |  |
| **Intervention benefits** |  |  |  |  |
| How might the benefits of the trial intervention(s) differ between participants experiencing socioeconomic disadvantage compared with those who are not? |  |  |  |  |
| **Intervention harms** |  |  |  |  |
| How might the possible harms of the trial intervention(s) differ between participants experiencing socioeconomic disadvantage compared with those who are not? |  |  |  |  |
| **Subgroup analyses** |  |  |  |  |
| How should variation between socioeconomic groups in the target population be explored? Should there be planned subgroup analyses? |  |  |  |  |
| **Interim analyses** |  |  |  |  |
| How should any interim analysis handle variation between socioeconomic groups in the target population? |  |  |  |  |
| **Stopping Triggers** |  |  |  |  |
| How should any rules to stop the trial early on intervention safety or intervention benefit grounds handle variation between socioeconomic groups in the target population? |  |  |  |  |

**Worksheet C.4: Factors that might affect the reporting and dissemination of trial results**

|  | **Pockets** | **Prospects** | **Places** | **Other(s)** |
| --- | --- | --- | --- | --- |
| **Planning reporting** |  |  |  |  |
| How, and in what way, are people experiencing socioeconomic disadvantage involved in planning the reporting and dissemination of the trial results, compared with those who are not? |  |  |  |  |
| **Dissemination engagement** |  |  |  |  |
| How, and in what way, might your reporting and dissemination of trial results limit engagement of people experiencing socioeconomic disadvantage compared with those who are not? Think about both the mode (i.e., how) and content (i.e., what) by which you are disseminating your results. |  |  |  |  |

**Worksheet D: What measures can I put in place to address the identified factors that might prevent optimal participation of people experiencing socioeconomic disadvantage?**

Use this worksheet to summarise the key factors you have identified that could prevent people experiencing socioeconomic disadvantage from fully participating in the trial, along with measures to mitigate the effect of those factors and their cost. Add extra rows as needed.

Innovation will be needed to generate strategies to address any identified barriers. We recommend seeking guidance from public contributors with relevant lived experiences to generate measures that will help to strengthen the approach. We also encourage researchers to document, publish, and review the strategies they develop whilst using this framework, in order to assist other researchers in developing their own strategies.

| **Factors that may prevent full community participation** | **Proposed measures (several options may be needed)** | **Cost of measures** |
| --- | --- | --- |
|  |  |  |
|  |  |  |
|  |  |  |
|  |  |  |
|  |  |  |
|  |  |  |
|  |  |  |
|  |  |  |
|  |  |  |

**Appendix 1**

**Framework background and next steps**

The National Institute for Health and Care Research (NIHR) initiated the INCLUDE initiative in 2017. The Medical Research Council (MRC) Hubs for Trials Methodology Research Recruitment and Retention Working Group were concurrently starting efforts to improve representation within trials, particularly minority ethnic individuals.

The two groups came together in late 2018 to develop a research grant proposal for work on inclusion in trials. Work on the INCLUDE Ethnicity Framework began in earnest in July 2019, and the complete Framework was launched in October 2020.

In June 2019 the Medical Research Council (MRC) Hubs for Trials Methodology Research became part of the MRC-NIHR Trials Methodology Research Partnership (TMRP). The Trial Conduct TMRP working group established the Inclusivity sub-group, which had its first meeting in July 2020, and based on discussions at this meeting, work began on the INCLUDE Socioeconomic Framework in November 2020. In January 2021, a grant application co-led by Frances Sherratt, Heidi Gardner, and Katie Biggs was awarded from The University of Liverpool Early Career and Returners’ Fund to fund public contributors’ time on the project. A public contributors’ group with six members was assembled in February 2021.

In the future, we would like to evaluate this framework to consider how useful and effective it is at supporting researchers to identify and work to address barriers to making clinical trials inclusive for patients from socioeconomically disadvantaged backgrounds. We are aware of work evaluating the INCLUDE Ethnicity Framework (e.g. [10]), which we expect to build on with our evaluation. Findings from the evaluation will be used to inform the development of current and future frameworks designed to improve inclusivity in research. Furthermore, members of MRC-NIHR Trials Methodology Research Partnership Inclusivity working group are currently in the process of appointing a PhD student who will work on a project exploring the possibility of combining several recently developed frameworks for different under-served groups; their primary focus will be on combining Frameworks covering ethnicity, socioeconomic disadvantage, and people without capacity to give consent. This may grow to include additional under-served groups as new frameworks are developed over the coming years. Doing so will help to address the intersectionality of many under-served characteristics, as well as ensuring trial teams are able to efficiently use their time to design inclusive trials in advance of submitting funding applications.

**Phases of developing the NIHR INCLUDE Socioeconomic Disadvantage Framework and team involvement**

**Phase 1: Defining socio-economic disadvantage and considering what is needed**

| **Name** | **Background/Perspective** |
| --- | --- |
| Katie Biggs | Research Fellow, University of Sheffield |
| Heidi Gardner | Research Fellow, University of Aberdeen. |
| Fran Sherratt | Research Fellow, University of Liverpool. |
| Firoza | Public Contributor |
| John Roberts | Public Contributor |
| Carolyn Graham, | Public Contributor |
| Philip Bell, | Public Contributor |
| Clara Martins de Barros | Public Contributor |
| Bola Aina | Public Contributor |

**Phase 2: Developing an initial draft of the Framework**

| **Name** | **Background/Perspective** |
| --- | --- |
| Katie Biggs | Research Fellow, University of Sheffield |
| Heidi Gardner | Research Fellow, University of Aberdeen. |
| Fran Sherratt | Research Fellow, University of Liverpool. |
| Firoza | Public Contributor |
| John Roberts | Public Contributor |
| Carolyn Graham, | Public Contributor |
| Philip Bell, | Public Contributor |
| Clara Martins de Barros | Public Contributor |
| Bola Aina | Public Contributor |

**Phase 3: Discussing that draft with a wider stakeholder group**

| **Name** | **Background/Perspective** |
| --- | --- |
| Katie Biggs | Research Fellow, University of Sheffield |
| Heidi Gardner | Research Fellow, University of Aberdeen. |
| Fran Sherratt | Research Fellow, University of Liverpool. |
| Firoza | Public Contributor |
| Clara Martins de Barros | Public Contributor |
| Philip Bell | Public Contributor |
| Carolyn Graham | Public Contributor |
| John Roberts | Public Contributor |
| Clare Bambra | Professor, Newcastle University |
| Teresa Crew | Senior Lecturer, Bangor University |
| Hanne Bruhn | Research Fellow, University of Aberdeen |
| Talia Isaacs | Associate Professor, University College London |
| Oonagh Ward, | Head of Research and Innovations Infrastructures, Health Research Board |
| Andrew Farmer, | Director of Health Technology Assessment Programme, National Institute for Health and Care Research (NIHR) |
| Jeremy Taylor | Director for Public Voice, NIHR |
| Michelle O’Shaughnessy | Consultant Nephrologist, University Hospital Galway |
| Frances Shiely | Senior Lecturer, University College Cork |
| Kate Fryer | Research Associate, University of Sheffield |
| Jon Dickson | Senior Clinical Lecturer, University of Sheffield |
| Dan Beever | Study Manager, University of Sheffield |
| Judith Cohen | Director of Hull Health Trials Unit, Hull York Medical School |
| Helen Hancock | Professor, Newcastle University |
| María José Pavez | Project coordinator, Grampian Health & Diversity Network |
| Rebecca Maier | Deputy Lead for Clinical Trials and Engagement, Newcastle University |
| Gosala Gopalakrishnan | Clinical Trials Manager, Imperial College London |

**Phase 4: Modifying the draft based on feedback from stakeholders**

| **Name** | **Background/Perspective** |
| --- | --- |
| Katie Biggs | Research Fellow, University of Sheffield |
| Heidi Gardner | Research Fellow, University of Aberdeen. |
| Fran Sherratt | Research Fellow, University of Liverpool. |

**Phase 5: Stakeholder feedback on the modified draft**

| **Name** | **Background/Perspective** |
| --- | --- |
| Katie Biggs | Research Fellow, University of Sheffield |
| Heidi Gardner | Research Fellow, University of Aberdeen. |
| Fran Sherratt | Research Fellow, University of Liverpool. |
| Firoza | Public Contributor |
| Clara Martins de Barros | Public Contributor |
| Philip Bell | Public Contributor |
| Carolyn Graham | Public Contributor |
| John Roberts | Public Contributor |
| Clare Bambra | Professor, Newcastle University |
| Teresa Crew | Senior Lecturer, Bangor University |
| Hanne Bruhn | Research Fellow, University of Aberdeen |
| Talia Isaacs | Associate Professor, University College London |
| Oonagh Ward, | Head of Research and Innovations Infrastructures, Health Research Board |
| Andrew Farmer, | Director of Health Technology Assessment Programme, National Institute for Health and Care Research (NIHR) |
| Jeremy Taylor | Director for Public Voice, NIHR |
| Michelle O’Shaughnessy | Consultant Nephrologist, University Hospital Galway |
| Frances Shiely | Senior Lecturer, University College Cork |
| Kate Fryer | Research Associate, University of Sheffield |
| Jon Dickson | Senior Clinical Lecturer, University of Sheffield |
| Dan Beever | Study Manager, University of Sheffield |
| Judith Cohen | Director of Hull Health Trials Unit, Hull York Medical School |
| Helen Hancock | Professor, Newcastle University |
| María José Pavez | Project coordinator, Grampian Health & Diversity Network |
| Rebecca Maier | Deputy Lead for Clinical Trials and Engagement, Newcastle University |
| Gosala Gopalakrishnan | Clinical Trials Manager, Imperial College London |
| Shaun Treweek | Professor of Health Services Research, University of Aberdeen. White British researcher, working class background (though now firmly middle-class), first person in family to go to university. |

**Phase 6: Applying the Framework**

| **Name** | **Background/Perspective** |
| --- | --- |
| Ongoing work | Ongoing work |

**Phase 7: Packaging the Framework, examples, and other materials**

| **Name** | **Background/Perspective** |
| --- | --- |
| Katie Biggs | Research Fellow, University of Sheffield |
| Heidi Gardner | Research Fellow, University of Aberdeen. |
| Fran Sherratt | Research Fellow, University of Liverpool. |
| Shaun Treweek | Professor of Health Services Research, University of Aberdeen. |
| Karen Beveridge | Administrative Assistant, University of Aberdeen. |

**References**

1. NIHR. Improving inclusion of under-served groups in clinical research: Guidance from INCLUDE project. 2022 v2.0. <https://www.nihr.ac.uk/documents/improving-inclusion-of-under-served-groups-in-clinical-research-guidance-from-include-project/25435>
2. Oakes JM, Rossi PH. The measurement of SES in health research: current practice and steps toward a new approach. Soc Sci Med. 2003;56(4):769-84.
3. Raina SK. Use of socioeconomic status scales in medicine and public health. J Family Med Prim Care. 2015;4(1):156-.
4. Scottish Government. Consultation on the socio-economic duty: Analysis of responses. 2017.
5. Ministry of Housing CLG. English Indices of Deprivation 2019 2019 [Available from: <https://www.gov.uk/government/statistics/english-indices-of-deprivation-2019>.
6. Clelland D, Hill C. Deprivation, policy and rurality: The limitations and applications of area-based deprivation indices in Scotland. Local Economy. 2019;34(1):33-50.
7. Government S. Child Poverty Strategy for Scotland - Our Approach - 2014-2017 2014 [Available from: <https://www.gov.scot/publications/child-poverty-strategy-scotland-approach-2014-2017/>.
8. Scottish Government. Consultation on a Child Poverty Bill for Scotland: Analysis of Responses. 2016.
9. FitzGerald C, Hurst S. Implicit bias in healthcare professionals: a systematic review. BMC medical ethics. 2017;18(1):19-.
10. Morris, L, Dumville, J, Miah N, Treweek, S, Curtis F, Bower P. Evaluating a tool to improve engagement and recruitment of under-served groups in trials. Trials 2022, 23: 867. <https://doi.org/10.1186/s13063-022-06747-2>

Acknowledgements

This work has involved and been supported by the following:


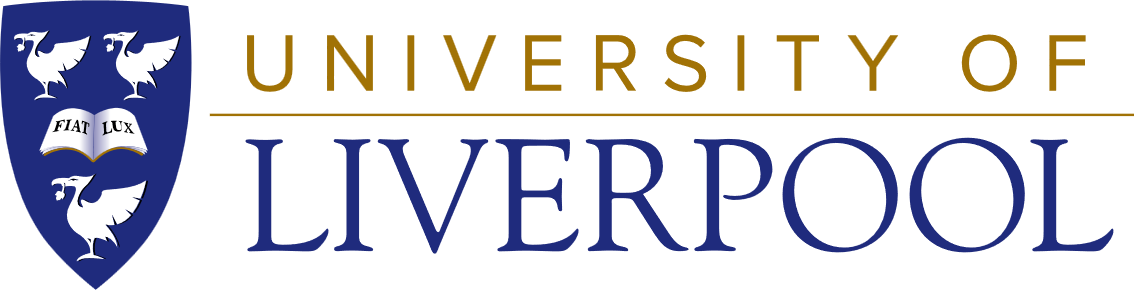


University of Liverpool Early Career

Researchers and Returners Fund


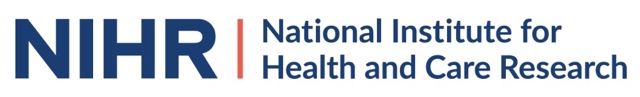


[National Institute of Health and Care Research](https://www.nihr.ac.uk/explore-nihr/academy-programmes/fellowship-programme.htm#one)

[Pre-doctoral Fellowship Programme](https://www.nihr.ac.uk/explore-nihr/academy-programmes/fellowship-programme.htm#one)

**
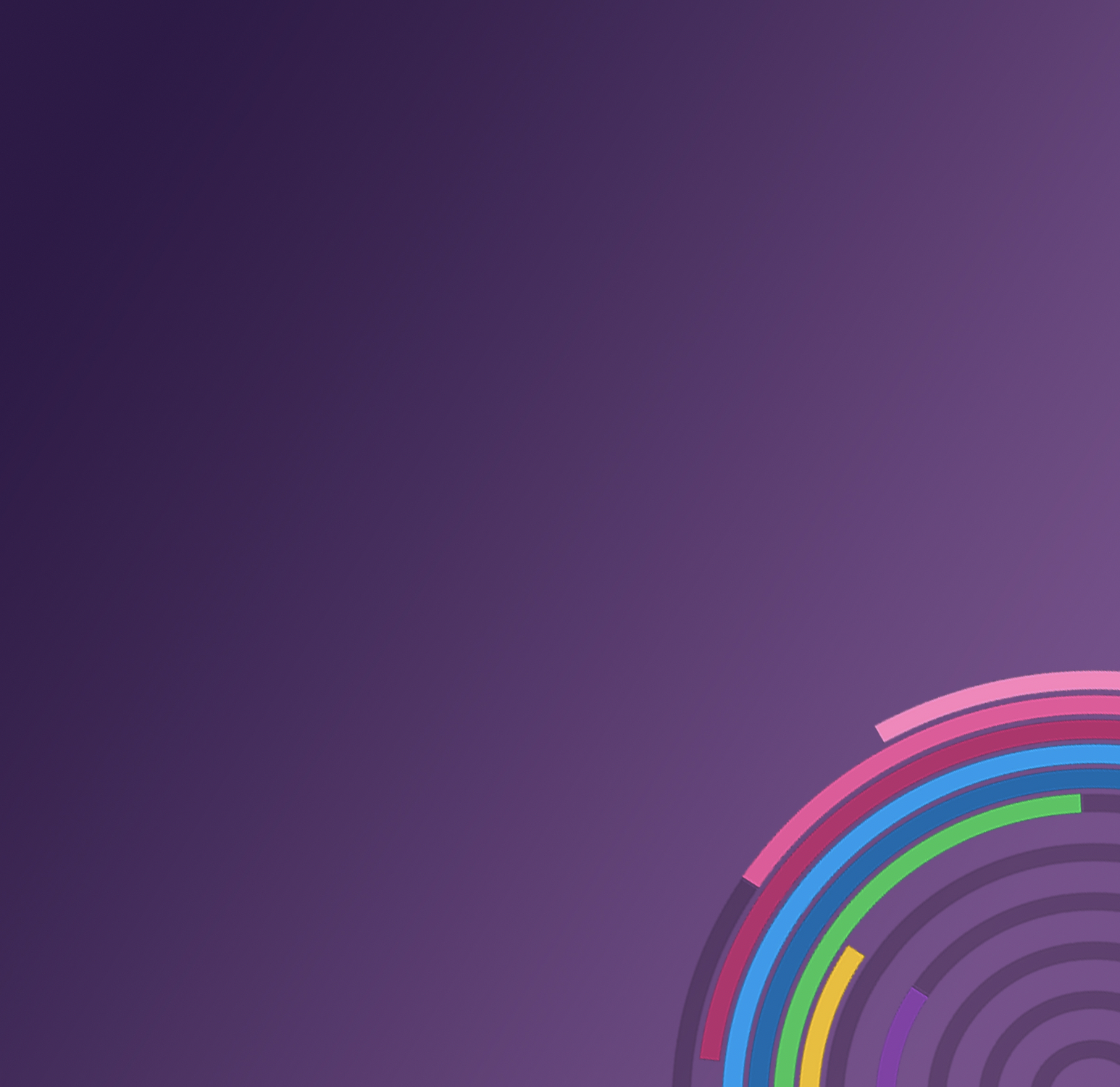
**
