# Supplementary material for: Making trials more inclusive of people experiencing socioeconomic disadvantage: developing the INCLUDE socioeconomic disadvantage framework
Source: Trials. 2026 Jan 14;27:123. doi: 10.1186/s13063-026-09448-2 (PMC12888448; doi:10.1186/s13063-026-09448-2)
Supplement: Supplementary file 7 — Additional file 7. [file 13063_2026_9448_MOESM7_ESM.docx]

**
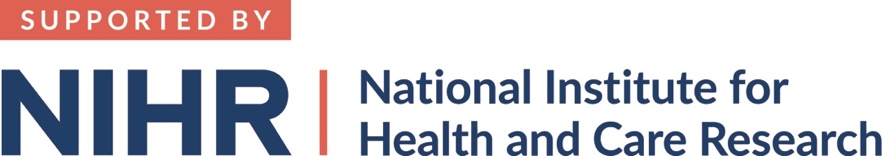

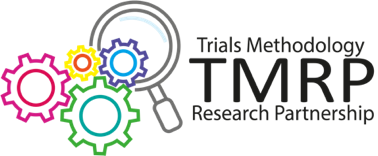

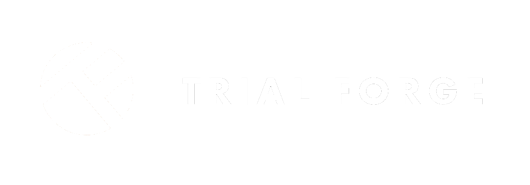

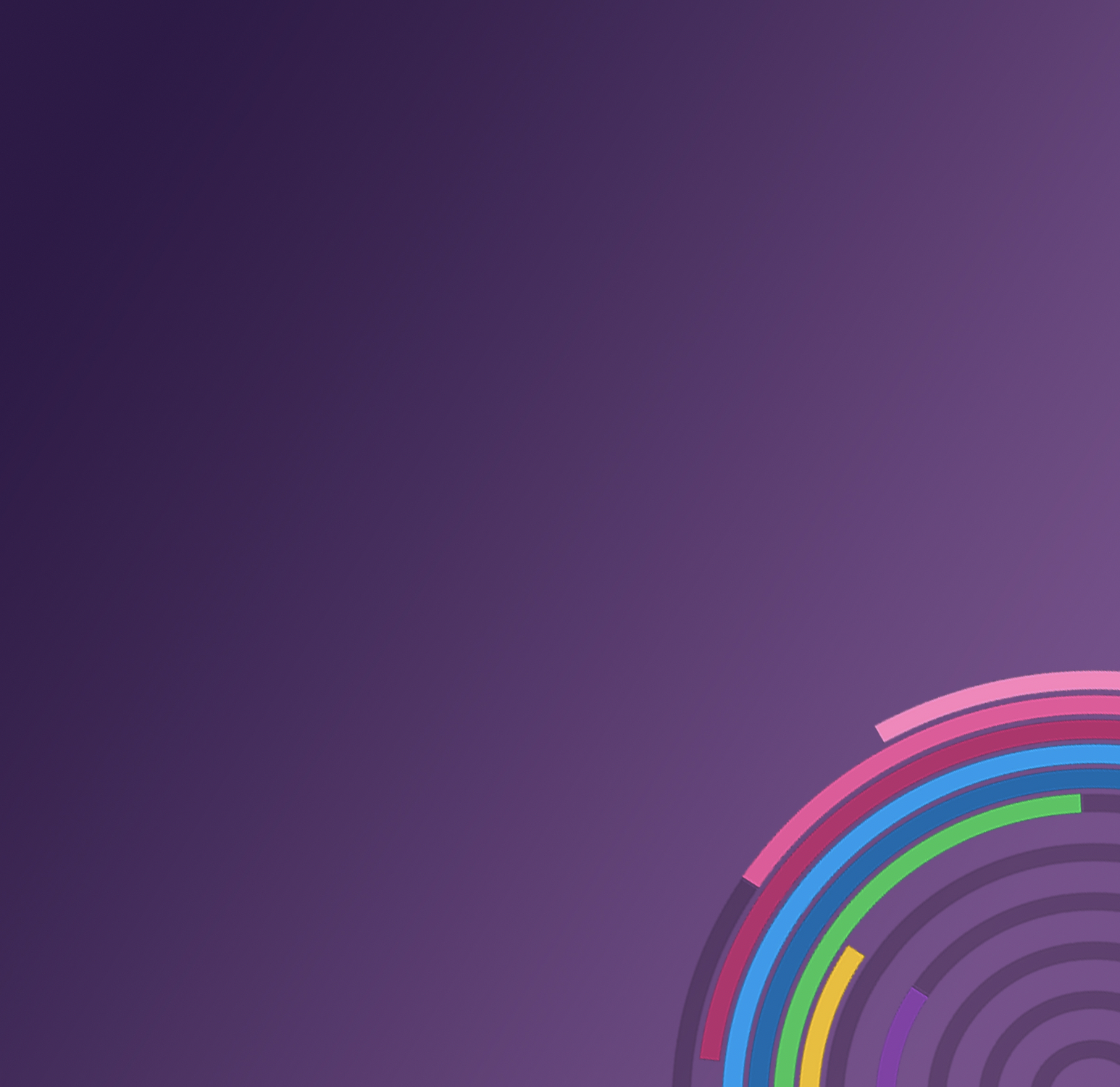
**

Additional file 7 Example completed Framework for REEACT trial

THE NIHR INCLUDE SOCIOECONOMIC DISADVANTAGE FRAMEWORK

NIHR INCLUDE Socioeconomic Disadvantage Framework:

Making clinical research more accessible to individuals experiencing socioeconomic disadvantage

Scope of framework

This framework has been designed to aid researchers, who are designing clinical trials, to consider barriers to including patients from socioeconomically disadvantaged backgrounds in their trial. The framework can also help researchers to develop strategies to attempt to address such barriers in order to improve the design and conduct of clinical research. Although this framework was developed with UK-based clinical trials in mind, aspects may also be relevant to different types of research and research conducted in populations outside of the UK. Whilst this framework focuses on socioeconomic disadvantage, the list of underserved groups in clinical research is extensive (1) and researchers need to be aware of this when identifying barriers to research and developing to strategies to address barriers.

What is socioeconomic disadvantage?

There is no consensus on a definition of socioeconomic status, nor does a widely accepted measure of socioeconomic status exist (2). There are many different domains of socioeconomic status, but it is typically measured as a combination of income, employment and education (3). In general terms, being socioeconomically disadvantaged includes living in less favourable social and economic circumstances than the majority of others in the same society (4).

Socioeconomic disadvantage is complex, multidimensional and can be dynamic; one’s socioeconomic status is not necessarily the same throughout the life course, and events can change socioeconomic status over time. We are aware that the language and terminology used to describe socioeconomic disadvantage can be sensitive. We would welcome any feedback and suggestions for improvement that you may have at: [info@trialforge.org](mailto:info@trialforge.org)

Applying ‘The 3 Ps’

It is important to note that socioeconomic disadvantage is *not* something that you can always see, and we therefore can’t make assumptions about people’s backgrounds or experiences. Rather than attempting to identify people, we encourage researchers to identify the potential barriers to clinical trial participation for those from socioeconomically disadvantaged backgrounds and develop and implement solutions.

Simplifying clinical trial processes, building trusting relationships, working with patients and public contributors from socioeconomically disadvantaged backgrounds, and reducing logistical barriers (e.g. through compensation, Internet access, trial information in alternative formats etc.) will contribute to making clinical trials more accessible and is likely to increase representation of people experiencing socioeconomic disadvantage in trials.

Indexes of socioeconomic disadvantage, which link disadvantage domains to postcodes (e.g. English Indices of Deprivation 2019 (5)), have become increasingly sophisticated. However, such indexes remain contentious due to conceptual and practical issues, with particular concerns about their applicability to rural areas (6). With this in mind we have chosen to develop the NIHR INCLUDE Socioeconomic Disadvantage Framework using categories adapted from the UK Government’s Child Poverty Strategy 2014-2017 (7), in addition to drawing on associated consultation work linked to the strategy (8).

‘The 3 Ps’ are a practical way of understanding socioeconomic disadvantage, providing us with a way of questioning our own activities in terms of trial design, so that we can effectively highlight the specific areas that make a trial inaccessible or unacceptable to people experiencing socioeconomic disadvantage. The 3Ps are not intended to be used as a definitive list of the experiences that socioeconomic disadvantage brings, rather, they are a nudge to ensure that trial teams think broadly when completing the worksheets that follow. We adapted ‘The 3 Ps’ from the Child Poverty Strategy work, which entailed three overarching target outcomes to address poverty, and developed the present framework with input from public contributors who described themselves as being from low-income backgrounds or in receipt of government benefits.

Indicator examples (not exhaustive) of socioeconomic disadvantage are grouped under one of three headings as below (see also Table 1):

1. Pockets – Indicators closely linked with income and economic resource availability
2. Prospects – Indicators closely linked with wellbeing and life chances
3. Place – Indicators closely linked with housing and local environment

**Table 1.** The 3Ps and associated example indicators of socioeconomic disadvantage

| **Pockets** *(Income and economic resource availability)* | **Prospects** *(Expectations and life chances)* | **Place** *(Housing and the local environment)* |
| --- | --- | --- |
| Benefits (e.g. uptake, adequacy, sanctions)  Unemployment  Low income  Childcare  Food poverty / use of food banks  Limited/no access to technological resources  Feeling powerless/ vulnerable due to financial circumstances  Covert situations within relationships (e.g. financial abuse) | Mental health  Household type (e.g. lone parent)  Educational attainment  Literacy  Health literacy  Co-morbidities  Low self-confidence or motivation, which plays into perceived power disparities and mistrust  No/limited access to sources of reliable health/trial information  Acceptance of ‘how it is’  Intersectionality with other underserved and/or vulnerable groups | Housing  Being homeless  Being part of a traveller community  Being in prison  Being an immigrant or refugee  No/limited access to transport systems  No/limited access to community services  Local labour market  Local services (e.g. access to childcare)  Healthcare access and engagement |

We encourage researchers to consider the 3Ps as a minimum when designing a study. Research has shown that other aspects of identity linked to social inequalities intersect with socioeconomic status, which results in people from minority ethnic groups, people experiencing physical and/or learning disabilities, people living with mental ill-health, people from the LGBTQIA+ community, and women, being at a higher risk of experiencing socioeconomic disadvantage. Furthermore, where someone lives can influence inequalities and how they are experienced. We encourage trial teams to think carefully about whether and how socioeconomic status intersects with these experiences, and work to implement facilitators and alleviate barriers across groups.

INCLUDE Key Questions

This NIHR INCLUDE Socioeconomic Disadvantage Framework is designed to encourage trial teams to do everything possible to make their trials relevant to the people that the results are likely to impact (often patients) and those expected to apply them (often healthcare professionals). The four questions below are intended to prompt trial teams to think about who should be involved as participants, and how to facilitate their involvement as much as possible. To identify the barriers and potential solutions to participation, we recommend that trial teams work through the questions below in partnership with patient and public contributors with experience of socioeconomic disadvantage. This is an exercise that should be completed **as a team**, with representation across all of the stakeholders that will ultimately be tasked with designing, delivering, and reporting on the trial.

Note that:

- *‘Intervention*’ means the treatment, initiative or service being evaluated.
- ‘*Comparator*’ means what the intervention is being compared to.
- ‘*Effective*’ means the intervention provides important benefits for people with the disease or condition that is the focus of the trial.

We recommend that trial teams think through the answers to the four Key Questions (below), with the aid of the provided worksheets:

| **Q1. Who should my trial results apply to?** |  |
| --- | --- |
| Which people could benefit from the intervention if it is found to be effective, or benefit from not having it if it is found to be ineffective and/or harmful? Are people from socioeconomically disadvantaged backgrounds routinely omitted from trials in your population or disproportionately affected by the condition or disease? | |
| **Q2. Are people from different socioeconomic backgrounds likely to respond to the intervention in different ways?** | |
| Could socioeconomic factors influence the way the people identified in Question 1 might respond to, or engage with, the intervention(s) being tested? How might the 3 Ps (see Table 1) affect how people will respond to, or engage with the intervention or the way that their health may change as a result of the intervention? | |
| **Q3. Will my trial intervention and/or comparator make it harder for people from different socioeconomic backgrounds to take part in the trial?** | |
| How might the intervention and/or comparator, including how they are delivered, make it harder for some people in the community to take part in the trial? How might the 3 Ps (see Table 1) make it harder for some people to access or accept the intervention or comparator? | |
| **Q4. Will the way I have planned and designed my trial make it harder for people from different socioeconomic backgrounds to take part?** | |
| How might elements of trial design, such as patient and public involvement (i.e. whether it is representative of the patient population), eligibility criteria, or the recruitment and consent process, make it harder for some people in the community to take part? | |

WORKSHEETS

The four worksheets are intended to be used by trial teams, including relevant stakeholders such as patient and public contributors, to ensure that people experiencing socioeconomic disadvantage are considered at the trial design stage.

The worksheets may cover issues that some trial teams already think about. The intention is that the worksheets will help to highlight issues consistently across trials for all trial teams, as well as raising some questions that may not be routinely considered at present.

Identifying people that may be experiencing socioeconomic disadvantage can be harmful, as the presence of implicit biases among health professionals can influence the quality of clinical care patients receive (9). Instead of trying to make judgements about people’s social and economic circumstances, we encourage trials teams to focus on improving the overall accessibility of their trial in general. This will improve how people access trials across the spectrum of socioeconomic status, whilst minimising the risk of arbitrarily and harmfully labelling people.

**We encourage trial teams to consider using the 3Ps; Pockets – income and resource availability, Prospects – expectations and life chances, and Places – housing and the local environment, as a starting point when completing the worksheets. See Table 1 for more information on our definition of socioeconomic disadvantage, and where the 3Ps have been used elsewhere.**

Before completing the worksheets, you should have answered Question 1 of the INCLUDE Key Questions…

| **Q1. Who should my trial results apply to?** |  |
| --- | --- |
| Trial details:   - Population: Diagnoses of depression (confirmed by PHQ9 at screening), recruited via GP - Interventions: 2 online CBT interventions - Comparator: Usual care - Outcomes: PHQ-9, other self-report measures - Study type: Individually randomised controlled, 2-arm, multicentre trial - Recruitment via GPs - Patient interview topics include computer literacy; views about CBT in future without consulting a health practitioner - Interviews with health professionals and managers   We identified people from socioeconomic disadvantaged groups as being important to include as this group experiences health inequalities. We also thought ethnic minority groups would be important to include due to cultural differences in mental health experiences, diagnosis, treatment, and recovery. Depression is a common mental health condition, which can be a result of the environment you live in and your life experiences, including trauma, which is experienced more by people experiencing socioeconomic disadvantage and racism.  The trial should apply to everyone who would meet the definition of depression. The study is conducted through GPs, and for people with a diagnosis of depression, but people are screened for depression on entry, so other people could be invited. If advertised more widely, people who do not, or cannot, access their GPs can be included. The intervention can be delivered in people’s home and in their own time and so could be given to a much wider population. | |

**Worksheet A: Are people from different socioeconomic backgrounds likely to respond to the intervention in different ways? (i.e. Q2 of the INCLUDE Key Questions)**

This worksheet includes questions to guide your thinking about the participation of people experiencing socioeconomic disadvantage when answering Question 2 of the INCLUDE Key Questions.

Could socioeconomic factors influence the way the people identified in question one might respond to, or engage with, the intervention(s) being tested? How might the 3 Ps (i.e. ‘Pockets’ [income and resource availability], ‘Prospects’ [expectations and life chances], and ‘Place’ [housing and the local environment]) affect how people will respond to, or engage with their condition or intervention?

|  | **Socioeconomic factors that might influence the effectiveness of the intervention for some groups** | | | |  |
| --- | --- | --- | --- | --- | --- |
|  | **Pockets** | **Prospects** | **Places** | **Other(s)** | |
| **Health condition** |  |  |  |  | |
| How might the prevalence of the health condition vary between people experiencing socioeconomic disadvantage and those who are not? | Low income can lead to increased stress, which can impact on depression and anxiety. | SES disadvantage is linked to inequalities across healthcare so investigators should always aim to include people who are experiencing SES disadvantage in research. | This trial is recruiting via GPs; Inverse care law shows access to GPS is worse in more deprived areas. | There are issues defining and measuring SES. | |
| How might the severity of the health condition vary between people experiencing socioeconomic disadvantage and those who are not? | The ability to cope with depression and its impacts might be reduced for people who do not have the means to pay for different types of support. | There is intersectionality between low SES and other groups that are disadvantaged such as people with disabilities, mental health conditions, ethnicity, which can contribute to increased severity, complications and impact. | SE disadvantage is linked to other health inequalities which could exacerbate the condition. | Is there data on severity of depression by SES? | |
| How might the presentation of the health condition vary between people experiencing socioeconomic disadvantage and those who are not? (this may include symptoms, type or pattern or rate of disease progression)? | As above.  Feelings of powerlessness or vulnerability, and lack of self-esteem can contribute to depression and be a barrier to seeking help, adherence and recovery. | As above. | Access to GPs and other healthcare can be lower for people experiencing socioeconomic disadvantage. | Is there data on type of depression by SES? | |
| **Perspectives** |  |  |  |  | |
| How might perceptions of the health condition and social stigma around it vary between people experiencing socioeconomic disadvantage and those who are not? | Feelings of powerlessness or vulnerability, and lack of self-esteem can contribute to depression and be a barrier to seeking help, adherence and recovery. | Health literacy may be lower in people experiencing socioeconomic disadvantage, which will affect whether people seek a diagnosis of depression. | Healthcare access may be lower for people experiencing socioeconomic disadvantage. | There may be intersectionality with ethnic minorities, and there may be differences in cultural perceptions of depression. | |
| How might ways of describing the disease vary between people experiencing socioeconomic disadvantage and those who are not? | Depression may be described as a direct consequence of or a feeling linked to financial insecurity, debt, poor housing, unemployment, and the struggle to "get by." | People may describe depression in relation to their circumstances.  Health literacy may be lower in people experiencing socioeconomic disadvantage, so language is important. | There may be different ways of approaching and talking to people about depression and the trial when recruiting outside of GPs as people may not be primed for this type of conversation. | There may be intersectionality with ethnic minorities, and there may be differences in cultural terms for depression. | |
| How might participants’ acceptability of, and adherence to, the intervention(s) vary between people experiencing socioeconomic disadvantage and those who are not? | There may be costs involved in being able to access an online intervention, such as data charges and having a suitable device. | Individuals not feeling educated enough to participate. | Individuals may not have a private space to do the intervention. | There may be intersectionality with ethnic minorities, and there may be differences in cultural perceptions of depression and the treatment. | |
| How or when might healthcare access vary between people experiencing socioeconomic disadvantage and those who are not? | People with a low income might find it harder to take time off work to participate or have less free time outside of work hours.  Limited access to technological resources. | A concrete diagnosis of depression is sometimes difficult, especially for those not engaging in services, such as those who are not registered with a GP.  Issues with health literacy and digital literacy (for access to healthcare apps). | In communities where interaction and trust with GP practices is a barrier to access, someone suggested social prescribers as a route to recruitment, as people can be referred to social prescribers for low mood, however not every practice has a social prescriber. | There was a discussion around the focus of the research question – which was for people registered with a GP – should our questions be wider than people already in the health service? If so, consider how to widen recruitment to the community. | |

**Worksheet B: Will my trial intervention and/or comparator make it harder for people from different socioeconomic backgrounds to take part in the trial? (i.e. Q3 of the INCLUDE Key Questions)**

How might the intervention and/or comparator, including how they are delivered, make it harder for some people in the community to take part in the trial? How might the 3 Ps (i.e. ‘Pockets’ [income and resource availability], ‘Prospects’ [expectations and life chances], and ‘Place’ [housing and the local environment]) make it harder for some people to access or accept the intervention or comparator?

This worksheet provides some questions to guide your thinking about participation of people experiencing socioeconomic disadvantage when answering Question 3 of the INCLUDE Key Questions.

|  | **Intervention and comparator factors that might affect how participants from some socioeconomic groups engage with the intervention and/or comparator*** | | | |  |
| --- | --- | --- | --- | --- | --- |
|  | **Pockets** | **Prospects** | **Places** | **Other(s)** | |
| **Who** |  |  |  |  | |
| How might the person delivering the intervention/comparator limit participation of people experiencing socioeconomic disadvantage compared to those who are not? | Online delivery and telephone support. | It was raised that people did not feel educated enough to take part, or that researchers don’t want people like them as they might not provide good answers. There was discussion of power imbalance, particularly with doctors and it was suggested that people use first names. | The intervention was self-delivered at home, which reduces issues with having to attend visits within certain hours. | Train lay advisors within the community to build trust within various communities, and potentially recruit, deliver the intervention and support participants with data collection. With adequate training, these lay advisors could help perform some of the study-related tasks such as visits. | |
| **What** |  |  |  |  | |
| How might the design/delivery of the intervention/comparator limit participation of people experiencing socioeconomic disadvantage compared to those who are not? | There may be issues paying for the data required, or with increased work hours and childcare meaning less free time to take part. | Limited health literacy can make interaction and self-directed delivery with the intervention difficult.  People reported not feeling educated enough to take part in a self-guided intervention. | People reported not having the space in their home to complete the intervention in private.  Access to GP or other venue to complete the intervention. |  | |
| How, and in what ways, were people experiencing socioeconomic disadvantage involved in selecting or designing the trial intervention/comparator compared with those who are not? | The trial team provided devices where possible as they were aware of access to devices.  It is important to include PPI with a low income. | It is important to include PPI with limited health literacy. | It is important to include PPI with reduced access to healthcare. | The PPI contributors were not detailed in the protocol and we did not try to find this elsewhere.  As this was retrospective, this was not possible to amend the level of involvement, or who was involved. | |
| **When** |  |  |  |  | |
| How might when the intervention/comparator is delivered (e.g. during working hours) and/or the frequency/intensity it is delivered (e.g. number of times it is delivered, over what period, time commitment for each session and overall) limit participation of people experiencing socioeconomic disadvantage compared with those who are not? | It is a self-delivered intervention so does have the flexibility for people to do it as and when they can.  People with a low income may have to work more hours, meaning they have less time to do the intervention. | It is a self-delivered intervention so does have the flexibility for people to do it as and when they can.  People with lower health literacy may find it difficult to motivate themselves to keep doing the intervention if it is difficult to understand. | It may be difficult for people to find a private space at the times they are available to do the intervention. |  | |
| **Where** |  |  |  |  | |
| How might where the intervention/comparator is delivered (e.g. hospital, general practice, local community venues) limit the participation of people experiencing socioeconomic disadvantage compared with those who are not? | It is a self-delivered intervention so there is flexibility in where it can be delivered. There may be issues with the cost of devices and data. | People may not have the space or social support to complete a self-delivered intervention at home. | One comment of relevance was that not everyone is near a major teaching hospital, and in rural, remote areas, public transport is increasingly difficult to use. This is supported by the increase in rural austerity, food insecurity and limited infrastructure in the UK over the past decade, which in turn has had a striking impact in access to healthcare and health research for those who are socioeconomically deprived and living rurally. |  | |
| **How** |  |  |  |  | |
| How might the mode of delivery (e.g. telephone, video-call, face-to-face, in groups) limit the participation of people experiencing socioeconomic disadvantage compared with those who are not? | Limited access to devices and data for a self-delivered intervention. | Self-delivered interventions could be more difficult for people with reduced health or digital literacy. | People who do not have a private space in their home could find it difficult to complete the intervention at home. | Lack of social support might affect adherence to the intervention. | |

*These factors are taken from TIDieR ([http://www.equator-network.org/reporting-guidelines/tidier/](about:blank)).

**Worksheet C: Will the way I have planned and designed my trial make it harder for people from different socioeconomic backgrounds to take part in the trial? (i.e. Q4 of the INCLUDE Key Questions)**

How might elements of trial design, such as patient and public involvement, eligibility criteria, or the recruitment and consent process, make it harder for some people in the community to take part? Factors that are known to contribute to socioeconomic disadvantage can be categorised as the ‘3Ps’; Pockets (income and resource availability), Prospects (expectations and life chances), and Places (housing and local environment). Please see Table 1 for examples.

This four-part worksheet provides some questions to guide your thinking about participation of people experiencing socioeconomic disadvantage when answering Question 4 of the INCLUDE Key Questions. Worksheet C is divided into four sub-worksheets:

C.1: Trial eligibility and information

C.2: Data collection

C.3: Data analysis

C.4: Reporting and dissemination

**Worksheet C.1: Trial eligibility and accessibility factors that might affect how some groups engage with the trial**

|  | **Pockets** | **Prospects** | **Places** | **Other(s)** |
| --- | --- | --- | --- | --- |
| **Eligibility** |  |  |  |  |
| How might eligibility criteria exclude people experiencing socioeconomic disadvantage for reasons other than their clinical eligibility for the trial (e.g. availability of medical history, language requirements, location, gender, age, discussing pregnancy, internet/mobile telephone access) compared with those who are not? | In this trial they had resources to supply people with a device if needed, so people were not excluded based on not having the right equipment. | In relation to literacy, health literacy and low education - The importance of addressing “what is research” before asking people to take part was discussed. It was raised in reference to building trust with potential participants, (and helping their families understand in the context of a trial in a care home). | People with limited access to GP may not have been able to get a depression diagnosis even if they have depression. As eligibility is assessed by a screening tool, this could be used on people without a formal diagnosis. |  |
| **Trial information** |  |  |  |  |
| How might the way(s) (and by whom) potential participants are made aware of the trial (e.g. posters in a clinic, letterheaded paper in brown envelope that has negative associations for participant [e.g. debt agency], documents written in plain English, who approaches the participant about the trial) limit the participation of people experiencing socioeconomic disadvantage compared to those who are not? | It is important to make any costs of taking part clear to participants and explain if there is any reimbursement or help with data costs that could be incurred. This is important to include in the layered information, and, for example, in the Easy Read versions. | In all trial redesign meetings people suggested informative videos and simplified consent information. ‘Easy Read’ documents were suggested, use of pictures and segmenting information, starting with what they need to know about taking part. | The trial should be widely advertised, not just in GP surgeries, but also via local community groups and community venues. |  |
| How might the mode and format of the information that tells potential participants about the trial (e.g. participant information leaflet, online, video) limit the participation of people experiencing socioeconomic disadvantage compared with those who are not? | People should be made aware in the information that they will not be out of pocket (if that is the case!) | All information needs to be in plain language using simple terms and clear messaging to aid understanding for those with lower literacy and lower health literacy. | Recruitment is via GPs and therefore access to GPs is important. The trial could be advertised in community venues to increase awareness. |  |
| How might potential participants’ cultural practices, beliefs and traditions change the way that people experiencing socioeconomic disadvantage perceive the information they are given compared with those who are not? | Perceived benefits and costs of participating and priorities around healthcare may differ among cultures. | Cultural beliefs may influence the perception of depression and of different treatments and research activities. | Cultural norms can influence what is considered legitimate forms of information. If the information is provided in a place that there is mistrust or historical trauma it may not be trusted. | There may be intersectionality with ethnic minorities, and there may be differences in cultural terms for depression. |

**Worksheet C.2: Trial data collection factors that might affect how some groups engage with the trial**

|  | **Pockets** | **Prospects** | **Places** | **Other(s)** |
| --- | --- | --- | --- | --- |
| **Who** |  |  |  |  |
| How might the people who collect data limit the participation of people experiencing socioeconomic disadvantage (e.g. role, power dynamics, relationship) compared with those who are not? | Internet/mobile telephone access is necessary for remote data collection in this trial. | Low self-confidence or motivation, which plays into perceived power disparities and mistrust. | Some people may not have the privacy and space in their home to complete the outcomes properly. | We need to collect the right data and monitor this throughout the trial. Try to find out people’s reasons for dropping out, interview people from underserved groups that withdraw where possible.  Power dynamics with recruiting staff. There was discussion of power imbalance, particularly with doctors and it was suggested that people use first names (which nurses tend to do more than doctors). |
| **What** |  |  |  |  |
| How, and in what way, were people experiencing socioeconomic disadvantage involved in selecting the trial outcomes compared with those who are not? | The PPI contributors were not detailed in the protocol and we did not try to find this elsewhere.  PPI contributors must be reimbursed for their input. | Health literacy is an important consideration when choosing outcomes, and for resourcing the support participants might need. | PPI contributors must be given the appropriate support to take part either online, or making venues accessible. | As this was retrospective, this was not possible to amend the level of involvement, or who was involved. |
| How might the trial outcomes themselves, or other data being collected (e.g. participant’s background information) limit the participation of people experiencing socioeconomic disadvantage compared with those who are not? | People might drop out if they no longer have time, or money to take part. | People might drop out as they do not like the intervention or do not want to complete the outcomes. This could be increased in this group due to lower health literacy. | Some people may not have the privacy and space in their home to complete the outcomes properly. |  |
| **When** |  |  |  |  |
| How might when the data is collected (e.g. appointments conflicting with work or childcare responsibilities) and/or the frequency/duration it is collected (e.g. number of follow up appointments, length of questionnaires) limit participation of people experiencing socioeconomic disadvantage compared with those who are not? | People with a low income may have to work more hours, meaning they have less time to do the follow-up. | Low health literacy could lead to self-completed questionnaires taking longer to complete and be more of a burden for these participants. | The questionnaires were self-completed, with options to complete via post, or over the phone, and did not need a visit, which should help people to complete them in a place suitable to them. |  |
| **Where** |  |  |  |  |
| How might the location where trial data are collected limit participation of people experiencing socioeconomic disadvantage compared with those who are not? (e.g. no/limited disposable income for additional public transport, don’t drive, poor transport links, feeling uncomfortable in setting) | The data collection being possible at home will remove barriers associated with visits that might incur costs.  There may be costs associated with online data completion, e.g. data costs, suitable devices. | Low self-confidence or motivation leading to more ‘Did Not Attends’ (DNAs).  Remote collection has issue around digital access and digital literacy. | The data collection being possible at home will remove barriers associated with access to GPs/clinics/other healthcare venues.  Private space in their home for completion could be limited. |  |
| **How?** |  |  |  |  |
| How might data collection methods limit the participation of people experiencing socioeconomic disadvantage compared with those who are not? (e.g. questionnaire format and length, additional clinical tests, mode of collection [e.g. option for various modes such as app, via telephone, paper questionnaire], type of remuneration and impact on government benefits i.e. voucher/cash) | There may be costs associated with online data completion. | Consider including additional support for participants around data collection, such as telephone calls or home visits. | The data collection being possible at home will remove barriers associated with access to GPs/clinics/other healthcare venues. |  |

**Worksheet C.3: Factors that might affect the analysis of trial results**

|  | **Pockets** | **Prospects** | **Places** | **Other(s)** |
| --- | --- | --- | --- | --- |
| **Representativeness** |  |  |  |  |
| When considering your approach to recruitment, how well represented might participants experiencing socioeconomic disadvantage be compared with the target population? | To assess this a measure of income is necessary. | To assess this a measure of education is necessary. | To assess this a measure of location is necessary. | Data collection around socioeconomic variables is often poor and inconsistent. |
| **Retention** |  |  |  |  |
| How might the accuracy and completeness of trial data collected differ between participants experiencing socioeconomic disadvantage compared with those who are not? | To assess this a measure of income is necessary.  People might drop out if they no longer have time, or money to take part. | To assess this a measure of education is necessary.  People might drop out of the find the language and processes difficult to engage with (literacy/digital literacy)  Intersectionality – affected by things outside of the trial. | To assess this a measure of location is necessary.  Issues around housing instability for the duration of the trial. |  |
| **Intervention benefits** |  |  |  |  |
| How might the benefits of the trial intervention(s) differ between participants experiencing socioeconomic disadvantage compared with those who are not? | To assess this a measure of income is necessary. | It is a self-delivered intervention that may not be as effective for people with low literacy or health literacy. | To assess this a measure of location is necessary. |  |
| **Intervention harms** |  |  |  |  |
| How might the possible harms of the trial intervention(s) differ between participants experiencing socioeconomic disadvantage compared with those who are not? | Too busy, or too worried about other things to notice harms, or differences in mood that other people might.  People could incur additional data costs that have a bigger impact on people on a low income. | Lower self-esteem due to limitations around literacy.  Acceptance of ‘how it is’  Self-esteem could be affected if people find it difficult to do the self-directed intervention. | Limited social support could inhibit awareness and knowledge of harms.  Referral to GPs for support (where harm is identified) could be more difficult in deprived areas where there is poorer access to GPs. |  |
| **Subgroup analyses** |  |  |  |  |
| How should variation between socioeconomic groups in the target population be explored? Should there be planned subgroup analyses? | Sub-group analysis around income was not reported. Unclear if the relevant data was collected.  This would have been under powered but could have been exploratory. | Sub-group analysis around education was not reported. Unclear if the relevant data was collected.  This would have been under powered but could have been exploratory. | Sub-group analysis around location was not reported. Unclear if the relevant data was collected.  This would have been under powered but could have been exploratory. | It might be useful to look at socioeconomic measures with sub-group analysis for some exploration of the issue. |
| **Interim analyses** |  |  |  |  |
| How should any interim analysis handle variation between socioeconomic groups in the target population? | Not applicable – no interim analysis reported. | | | |
| **Stopping Triggers** |  |  |  |  |
| How should any rules to stop the trial early on intervention safety or intervention benefit grounds handle variation between socioeconomic groups in the target population? | Not applicable – no stopping rules reported. | | | |

**Worksheet C.4: Factors that might affect the reporting and dissemination of trial results**

|  | **Pockets** | **Prospects** | **Places** | **Other(s)** |
| --- | --- | --- | --- | --- |
| **Planning reporting** |  |  |  |  |
| How, and in what way, are people experiencing socioeconomic disadvantage involved in planning the reporting and dissemination of the trial results, compared with those who are not? | People with low income should be included in the PPI contributors involved in dissemination. | People with limited health literacy should be included in the PPI contributors involved in dissemination. | People with limited access to health care settings and providers should be included in the PPI contributors involved in dissemination. |  |
| **Dissemination engagement** |  |  |  |  |
| How, and in what way, might your reporting and dissemination of trial results limit engagement of people experiencing socioeconomic disadvantage compared with those who are not? Think about both the mode (i.e., how) and content (i.e., what) by which you are disseminating your results. | Attendance at results meetings could be difficult for people if not funded, or if they cannot get time off work.  Reliance on digital channels may limit who sees the results due to data and device use. | Literacy considerations. | Access to the places that results are disseminated.  Disseminating via post and email may be problematic if people move often or change email addresses. | One-way dissemination can reinforce power dynamics. |

**Worksheet D: What measures can I put in place to address the identified factors that might prevent optimal participation of people experiencing socioeconomic disadvantage?**

Use this worksheet to summarise the key factors you have identified that could prevent people experiencing socioeconomic disadvantage from fully participating in the trial, along with measures to mitigate the effect of those factors and their cost. Add extra rows as needed.

Innovation will be needed to generate strategies to address any identified barriers. We recommend seeking guidance from public contributors with relevant lived experiences to generate measures that will help to strengthen the approach. We also encourage researchers to document, publish, and review the strategies they develop whilst using this framework, in order to assist other researchers in developing their own strategies.

| **Factors that may prevent full community participation** | **Proposed measures (several options may be needed)** | **Cost of measures** |
| --- | --- | --- |
| Literacy for understanding trial information, intervention content and outcomes | Language should be a simple as possible for the trial and the intervention.  Work with appropriate PPI.  Layered information.  Videos. | PPI costs for involvement.  Cost for video production, additional PPI. |
| Costs to taking part | Trial to provide any equipment needed, data and devices for remote delivery and transport. | Costs for devices for duration of intervention delivery.  Costs for data.  Costs for transport. |
| Too busy to complete the follow-up | Focus on primary outcomes.  Flexibility in how and when it can be completed.  Routine data collection. | Costs to access routine data – staff time for applications and contract, fees, time to undertake. |
| Power dynamics | Training for recruiting, intervention delivery and data collection staff. | Additional training time for site staff and trial manager/ research assistant. |
| Digital literacy necessary for intervention | Working with relevant populations prior to the trial to check digital literacy.  Include training and additional support. | Need time during set-up to undertake this work, and to be able to make necessary changes.  Cost for additional staff time.  Cost for involvement of organisations. |
| Potential for differential dropout | Try to find out people’s reasons for dropping out.  Interview people from underserved groups that withdraw where possible. | Cost for additional qualitative research with a focus on non-recruited people, and under-served groups. |
|  |  |  |
|  |  |  |

**Appendix 1**

**Framework background and next steps**

The National Institute for Health and Care Research (NIHR) initiated the INCLUDE initiative in 2017. The Medical Research Council (MRC) Hubs for Trials Methodology Research Recruitment and Retention Working Group were concurrently starting efforts to improve representation within trials, particularly minority ethnic individuals.

The two groups came together in late 2018 to develop a research grant proposal for work on inclusion in trials. Work on the INCLUDE Ethnicity Framework began in earnest in July 2019, and the complete Framework was launched in October 2020.

In June 2019 the Medical Research Council (MRC) Hubs for Trials Methodology Research became part of the MRC-NIHR Trials Methodology Research Partnership (TMRP). The Trial Conduct TMRP working group established the Inclusivity sub-group, which had its first meeting in July 2020, and based on discussions at this meeting, work began on the INCLUDE Socioeconomic Framework in November 2020. In January 2021, a grant application co-led by Frances Sherratt, Heidi Gardner, and Katie Biggs was awarded from The University of Liverpool Early Career and Returners’ Fund to fund public contributors’ time on the project. A public contributors’ group with six members was assembled in February 2021.

In the future, we would like to evaluate this framework to consider how useful and effective it is at supporting researchers to identify and work to address barriers to making clinical trials inclusive for patients from socioeconomically disadvantaged backgrounds. We are aware of work evaluating the INCLUDE Ethnicity Framework (e.g. [10]), which we expect to build on with our evaluation. Findings from the evaluation will be used to inform the development of current and future frameworks designed to improve inclusivity in research. Furthermore, members of MRC-NIHR Trials Methodology Research Partnership Inclusivity working group are currently in the process of appointing a PhD student who will work on a project exploring the possibility of combining several recently developed frameworks for different under-served groups; their primary focus will be on combining Frameworks covering ethnicity, socioeconomic disadvantage, and people without capacity to give consent. This may grow to include additional under-served groups as new frameworks are developed over the coming years. Doing so will help to address the intersectionality of many under-served characteristics, as well as ensuring trial teams are able to efficiently use their time to design inclusive trials in advance of submitting funding applications.

**Phases of developing the NIHR INCLUDE Socioeconomic Disadvantage Framework and team involvement**

**Phase 1: Defining socio-economic disadvantage and considering what is needed**

| **Name** | **Background/Perspective** |
| --- | --- |
| Katie Biggs | Research Fellow, University of Sheffield |
| Heidi Gardner | Research Fellow, University of Aberdeen. |
| Fran Sherratt | Research Fellow, University of Liverpool. |
| Firoza | Public Contributor |
| John Roberts | Public Contributor |
| Carolyn Graham, | Public Contributor |
| Philip Bell, | Public Contributor |
| Clara Martins de Barros | Public Contributor |
| Bola Aina | Public Contributor |

**Phase 2: Developing an initial draft of the Framework**

| **Name** | **Background/Perspective** |
| --- | --- |
| Katie Biggs | Research Fellow, University of Sheffield |
| Heidi Gardner | Research Fellow, University of Aberdeen. |
| Fran Sherratt | Research Fellow, University of Liverpool. |
| Firoza | Public Contributor |
| John Roberts | Public Contributor |
| Carolyn Graham, | Public Contributor |
| Philip Bell, | Public Contributor |
| Clara Martins de Barros | Public Contributor |
| Bola Aina | Public Contributor |

**Phase 3: Discussing that draft with a wider stakeholder group**

| **Name** | **Background/Perspective** |
| --- | --- |
| Katie Biggs | Research Fellow, University of Sheffield |
| Heidi Gardner | Research Fellow, University of Aberdeen. |
| Fran Sherratt | Research Fellow, University of Liverpool. |
| Firoza | Public Contributor |
| Clara Martins de Barros | Public Contributor |
| Philip Bell | Public Contributor |
| Carolyn Graham | Public Contributor |
| John Roberts | Public Contributor |
| Clare Bambra | Professor, Newcastle University |
| Teresa Crew | Senior Lecturer, Bangor University |
| Hanne Bruhn | Research Fellow, University of Aberdeen |
| Talia Isaacs | Associate Professor, University College London |
| Oonagh Ward, | Head of Research and Innovations Infrastructures, Health Research Board |
| Andrew Farmer, | Director of Health Technology Assessment Programme, National Institute for Health and Care Research (NIHR) |
| Jeremy Taylor | Director for Public Voice, NIHR |
| Michelle O’Shaughnessy | Consultant Nephrologist, University Hospital Galway |
| Frances Shiely | Senior Lecturer, University College Cork |
| Kate Fryer | Research Associate, University of Sheffield |
| Jon Dickson | Senior Clinical Lecturer, University of Sheffield |
| Dan Beever | Study Manager, University of Sheffield |
| Judith Cohen | Director of Hull Health Trials Unit, Hull York Medical School |
| Helen Hancock | Professor, Newcastle University |
| María José Pavez | Project coordinator, Grampian Health & Diversity Network |
| Rebecca Maier | Deputy Lead for Clinical Trials and Engagement, Newcastle University |
| Gosala Gopalakrishnan | Clinical Trials Manager, Imperial College London |

**Phase 4: Modifying the draft based on feedback from stakeholders**

| **Name** | **Background/Perspective** |
| --- | --- |
| Katie Biggs | Research Fellow, University of Sheffield |
| Heidi Gardner | Research Fellow, University of Aberdeen. |
| Fran Sherratt | Research Fellow, University of Liverpool. |

**Phase 5: Stakeholder feedback on the modified draft**

| **Name** | **Background/Perspective** |
| --- | --- |
| Katie Biggs | Research Fellow, University of Sheffield |
| Heidi Gardner | Research Fellow, University of Aberdeen. |
| Fran Sherratt | Research Fellow, University of Liverpool. |
| Firoza | Public Contributor |
| Clara Martins de Barros | Public Contributor |
| Philip Bell | Public Contributor |
| Carolyn Graham | Public Contributor |
| John Roberts | Public Contributor |
| Clare Bambra | Professor, Newcastle University |
| Teresa Crew | Senior Lecturer, Bangor University |
| Hanne Bruhn | Research Fellow, University of Aberdeen |
| Talia Isaacs | Associate Professor, University College London |
| Oonagh Ward, | Head of Research and Innovations Infrastructures, Health Research Board |
| Andrew Farmer, | Director of Health Technology Assessment Programme, National Institute for Health and Care Research (NIHR) |
| Jeremy Taylor | Director for Public Voice, NIHR |
| Michelle O’Shaughnessy | Consultant Nephrologist, University Hospital Galway |
| Frances Shiely | Senior Lecturer, University College Cork |
| Kate Fryer | Research Associate, University of Sheffield |
| Jon Dickson | Senior Clinical Lecturer, University of Sheffield |
| Dan Beever | Study Manager, University of Sheffield |
| Judith Cohen | Director of Hull Health Trials Unit, Hull York Medical School |
| Helen Hancock | Professor, Newcastle University |
| María José Pavez | Project coordinator, Grampian Health & Diversity Network |
| Rebecca Maier | Deputy Lead for Clinical Trials and Engagement, Newcastle University |
| Gosala Gopalakrishnan | Clinical Trials Manager, Imperial College London |
| Shaun Treweek | Professor of Health Services Research, University of Aberdeen. White British researcher, working class background (though now firmly middle-class), first person in family to go to university. |

**Phase 6: Applying the Framework**

| **Name** | **Background/Perspective** |
| --- | --- |
| Ongoing work | Ongoing work |

**Phase 7: Packaging the Framework, examples, and other materials**

| **Name** | **Background/Perspective** |
| --- | --- |
| Katie Biggs | Research Fellow, University of Sheffield |
| Heidi Gardner | Research Fellow, University of Aberdeen. |
| Fran Sherratt | Research Fellow, University of Liverpool. |
| Shaun Treweek | Professor of Health Services Research, University of Aberdeen. |
| Karen Beveridge | Administrative Assistant, University of Aberdeen. |

**References**

1. NIHR. Improving inclusion of under-served groups in clinical research: Guidance from INCLUDE project. 2022 v2.0. <https://www.nihr.ac.uk/documents/improving-inclusion-of-under-served-groups-in-clinical-research-guidance-from-include-project/25435>
2. Oakes JM, Rossi PH. The measurement of SES in health research: current practice and steps toward a new approach. Soc Sci Med. 2003;56(4):769-84.
3. Raina SK. Use of socioeconomic status scales in medicine and public health. J Family Med Prim Care. 2015;4(1):156-.
4. Scottish Government. Consultation on the socio-economic duty: Analysis of responses. 2017.
5. Ministry of Housing CLG. English Indices of Deprivation 2019 2019 [Available from: <https://www.gov.uk/government/statistics/english-indices-of-deprivation-2019>.
6. Clelland D, Hill C. Deprivation, policy and rurality: The limitations and applications of area-based deprivation indices in Scotland. Local Economy. 2019;34(1):33-50.
7. Government S. Child Poverty Strategy for Scotland - Our Approach - 2014-2017 2014 [Available from: <https://www.gov.scot/publications/child-poverty-strategy-scotland-approach-2014-2017/>.
8. Scottish Government. Consultation on a Child Poverty Bill for Scotland: Analysis of Responses. 2016.
9. FitzGerald C, Hurst S. Implicit bias in healthcare professionals: a systematic review. BMC medical ethics. 2017;18(1):19-.
10. Morris, L, Dumville, J, Miah N, Treweek, S, Curtis F, Bower P. Evaluating a tool to improve engagement and recruitment of under-served groups in trials. Trials 2022, 23: 867. <https://doi.org/10.1186/s13063-022-06747-2>

Acknowledgements

This work has involved and been supported by the following:


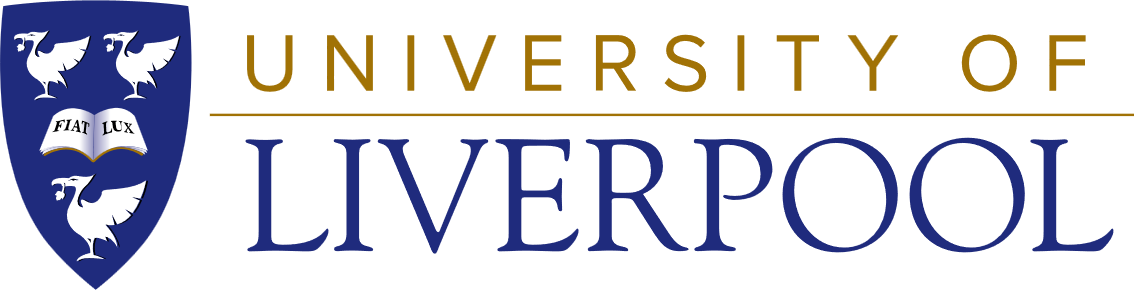


University of Liverpool Early Career

Researchers and Returners Fund


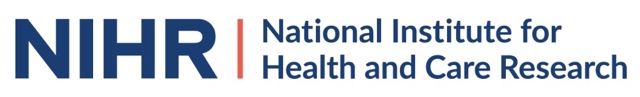


[National Institute of Health and Care Research](https://www.nihr.ac.uk/explore-nihr/academy-programmes/fellowship-programme.htm#one)

[Pre-doctoral Fellowship Programme](https://www.nihr.ac.uk/explore-nihr/academy-programmes/fellowship-programme.htm#one)

**
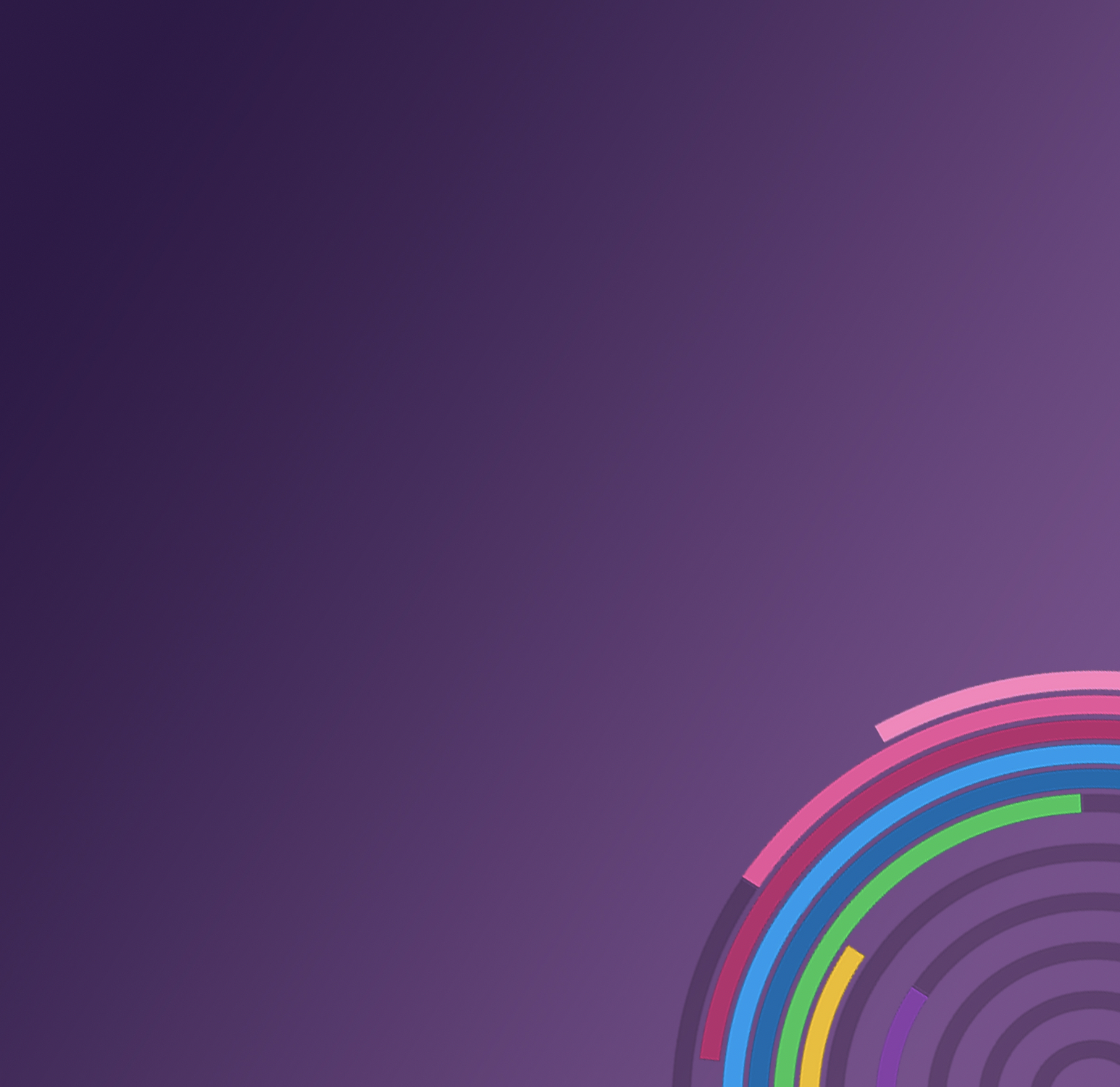
**
